# Supplementary material for: Human de novo mutation rates from a four-generation pedigree reference
Source: Nature. 2025 Apr 23;643(8071):427–36. doi: 10.1038/s41586-025-08922-2 (PMC12240836; doi:10.1038/s41586-025-08922-2)
Supplement: Supplementary file 1 — Supplementary Notes 1–10, Supplementary Figs. 1–52 and Supplementary References. [file 41586_2025_8922_MOESM1_ESM.pdf]

nature portfolio

https://doi.org/10.1038/s41586-025-08922-2

Supplementary information

Human de novo mutation rates from a four-generation pedigree reference

In the format provided by the authors and unedited

SUPPLEMENTARY INFORMATION

for “Human *de novo* mutation rates from a four-generation pedigree reference”

Table of Contents

SUPPLEMENTARY INFORMATION... 1

SUPPLEMENTARY NOTES 4

Note S1. Evaluation of cell-line-specific artifacts in previous studies 4

Note S2. Evaluation of known copy number variant (CNV) regions in phased genome assemblies 4

Note S3. Mobile element insertion (MEI) analysis.. 5

Note S4. Quantifying false positives and negatives in alignment-based *de novo* SNVs.......... 6

Note S5. Haplotype analysis of flanking SNVs to validate recurrent tandem repeat allele transmission 8

Note S6. Details on Chromosome Y analysis 8

Note S7. Evaluation of mutation spectra in unique and repeated regions 10

Note S8. Assembly quality terminology used in this study 11

Note S9. Evaluation of *de novo* SNV mutations..12

NoteS10. Evaluation of SV DNMs 14

SUPPLEMENTARY TABLES...17

SUPPLEMENTARY FIGURES...17

Figure S1: Evaluation of assembly contiguity...17

Figure S2: Evaluation of gaps in scaffolded Verkko assemblies. 18

Figure S3: Telomere completeness in phased genome assemblies. 19

Figure S4: Centromere completeness in phased genome assemblies..................................19

Figure S5: Evaluation of assembly quality. 20

Figure S6: Evaluation of misoriented regions with Strand-seq. 20

Figure S7: Evaluation of assembly phasing accuracy. 21

Figure S8: Chimeric contig in Verkko assembly...22

Figure S9: Flagger summary of possible problematic regions in phased assemblies.............23

Figure S10: Flagger evaluation of NA12879. 24
Figure S11: Ploidy summary of aligned Verkko genome assemblies to T2T-CHM13 reference. 25
Figure S12: Gene completeness assessment using compleasm. 26
Figure S13. The agreement among different SV callers in the curated truth set. 27
Figure S14: Non-reference mobile element insertion (MEI) analysis......................................28
Figure S15: Strand-seq-based inversion callset..29
Figure S16: Strand-seq inversion callset with respect to T2T-CHM13. 30
Figure S17: Inverted bases per haploid genome and per generation. 31
Figure S18: Summary of Strand-seq inversion genotypes. 32
Figure S19: Rare inversion at 15q25.2-25.3. 33
Figure S20: Rare, inverted duplication at 16q11.2. 34
Figure S21: Recombination breakpoint overlap. 35
Figure S22: Recombination breakpoint resolution of G3. 36
Figure S23: Recombination breakpoint refinement using phased genome assemblies. 37
Figure S24: Sharp and wide transition at recombination breakpoints. 38
Figure S25: Putative allelic gene conversion events (T2T-CHM13). 39
Figure S26: Male and female recombination breakpoints per sample and generation. 40
Figure S27: Recombination breakpoint hotspots (T2T-CHM13). 41
Figure S28: Summary of observed recombinant and nonrecombinant parental alleles. 42
Figure S29: Maternal and paternal recombination counts and their ratios per chromosome. .43
Figure S30: Biased distribution of recombination breakpoints (T2T-CHM13). 44
Figure S31: Recombination breakpoints and parental age..45
Figure S32: Evaluation of single-nucleotide DNMs using Element data. 46
Figure S33: Germline and postzygotic mutation spectrum and rates. 47
Figure S34: “Stutter” profiles at homozygous homopolymer loci using various sequencing technologies on sample G3-NA12879. 48
Figure S35: Read evidence from orthogonal technologies at a single homopolymer locus. ...49
Figure S36: Patterns of TR DNMs. 50
Figure S37: Summary of detected *de novo* SVs (n=41). 51
Figure S38: Predicting a donor site of *de novo* SVA insertion. 52
Figure S39: Predicting a donor site of *de novo* SVA insertion. 53
Figure S40: Example of Strand-seq libraries...54
Figure S41: Strand-seq data summary for G1-G3 samples. 55

Figure S42: Evaluation of cell-line-specific artifacts. 56
Figure S43: Assembly-based genotyping of known CNV regions. 57
Figure S44: MEI analysis summary. 58
Figure S45: Phased haplotypes and allele counts. 60
Figure S46: Transmission of flanking SNVs at recurrent TR locus. 61
Figure S47: Phylogenetic relationships of long-read Y assemblies and pedigree Y chromosomes. 63
Figure S48: Comparison of G1-NA12889 and T2T-CHM13 Y chromosome sequences. 64
Figure S49: Comparison of chrY assemblies. 65
Figure S50: Assembled chrX and chrY pseudoautosomal regions (PARs) across three generations. 66
Figure S51: Mutation spectra in unique and repeated regions. 68
Figure S52: Examples of false *de novo* insertions in phased genome assemblies. 69
REFERENCES 70

SUPPLEMENTARY NOTES

Note S1. Evaluation of cell-line-specific artifacts in previous studies

To evaluate the extent of single-nucleotide variants (SNVs) arising from cell line artifacts, we compared our callset with previous studies (Conrad et al. 2011; Eberle et al. 2017). For example, Eberle and colleagues cataloged ~1,869 *de novo* SNVs per individual (Eberle et al. 2017) indicating that cell line *de novo* events were ~23× more common than true *de novo* events. Although the data from Eberle et al. is not easily accessible, we were able to reevaluate those from Conrad and colleagues. In that study, there are a total of 3,236 mutations reported with respect to NCBI36 (hg18). Of those, we were able to liftOver 3,038 sites to GRCh38 coordinates. Among these, there are 48 germline *de novo* mutations (DNMs) and 888 predicted cell-line-specific DNMs. The rest are inherited variants and false positives (**Supplementary Fig. 42a**). Of the 48 predicted germline DNMs, 45 are also reported in the platinum DNM callset (see Results section *'De novo* SNVs and small indels', **Supplementary Table 10**). Only three germline DNMs are not seen in our callset (**Supplementary Fig. 42b**). There are 38 DNMs reported only in our manuscript and, of those, 10 were reported by Conrad and colleagues as 'False positive call' or 'no call'. So, 28 (no overlap with Conrad et al. 2011) are truly unique sites in our callset (**Supplementary Fig. 42c**) with 10 likely missed by Conrad and colleagues. Importantly, none of our reported DNMs were marked as cell line artifacts by Conrad et al. (2011). Further, we evaluated sequence properties of reported cell-line-specific DNMs (n=888, "cell line DNMs"). Specifically, we estimated the frequency of transition (Ti) and transversion (Tv) for various categories and found that the Ti/Tv ratio for true germline events was more than double (2.43 vs. 0.95) that of those originating from cell line artifacts.

Note: In order to distinguish true germline DNMs from somatic or cell line DNMs, Conrad et al. (2011) used two orthogonal validation approaches. The first one was a nested PCR amplification of putative DNMs followed by Illumina sequencing of pooled PCR products. The second experiment was based on hybridization capture of putative DNMs using Agilent SureSelect technology followed by SOLID sequencing (Conrad et al. 2011).

Note S2. Evaluation of known copy number variant (CNV) regions in phased genome assemblies

Here, we set to evaluate assembly-based genotyping of previously defined CNV clusters across the 2,504 1KG samples with respect to GRCh37 (Sudmant et al. 2015). First, we lifted over CNV coordinates to T2T-CHM13. We successfully lifted 3,073/3,263 CNVs, using the liftOver tool (Kent et al. 2002). We then attempted to extract the sequence of each CNV region from phased genome assemblies along with 50 kbp flanking sequence on each side. For regions (n=3,047) where both G2 parents were completely assembled

in a single contig, we evaluated inheritance of each given region in G3 samples (n=8). We used the size of each region across haplotypes as a proxy of the copy number. We then compared each child's haplotype to both parents and assigned it to the most likely parental haplotype based on size. In total, we assessed 3,047 CNV clusters out of which 2,772 (~91%) could be unambiguously assessed and show clear haplotype inheritance from G2 to at least four and more G3 samples. A small proportion of CNV regions (~9%) have more than half of the child's genotypes missing due to small differences between inherited alleles (**Supplementary Fig. 43a-b**). A large proportion of these can be explained by the marked assembly errors by Flagger (Liao et al. 2023) (~75%), while the rest of the inconsistencies are likely caused by the sensitivity of our algorithm prototype. We note that our algorithm assigns child alleles to the most similar parental alleles based on the calculation of percentage difference between observed alleles in a child and all possible inherited parental alleles. As a consequence, smaller structural variants (SVs) are penalized more since even a small size change has a large effect on reported percentage difference. We show an example of a fully genotyped region in **Supplementary Fig. 43c** where the size of inherited alleles in children match the size of inherited parental alleles, as expected.

Note S3. Mobile element insertion (MEI) analysis

Using xTea (v 0.1.9) (Chu et al. 2021) to analyze the PacBio long reads, we identified non-reference MEI events. We classified all non-reference Alu*,* LINE-1, and SVA insertions. In G1-G3, we found 2,161 full-length Alu insertions (**Supplementary Fig. 14a**), 398 LINE-1 insertions, and 151 SVA insertions (**Supplementary Fig. 14b**). We identified 112 LINE-1 insertions that were either full-length or near full-length (at least 5500 bp).

To examine the subfamilies of the non-reference MEIs, the sequence for each Alu element greater than 240 bp in length was aligned using MUSCLE (v.3.8.31) (Edgar 2004). Genetic similarities among all possible pairs of sequences were calculated, and principal components were obtained by eigendecomposition of the similarity matrix. The relationship between known Alu element subfamilies and the non-reference Alu element insertion sequences is shown in **Supplementary Figure 44a**. Most of the non-reference Alu insertions cluster closely around known AluYa and AluYb subfamily sequences. This process was repeated for full-length LINE-1 insertions (**Supplementary Fig. 44b**) and SVA insertions (**Supplementary Fig. 44c**). As expected, most non-reference LINE-1 insertions cluster around the L1HS subfamily, and most SVA insertions cluster around the SVAE and SVAF subfamilies. Using BLAT (James Kent 2002), we identified LINE-1 insertions in the reference genome that were most likely to be source elements for each of our non-reference, full-length LINE-1 insertions. For 101 of the 112 insertions, we were able to identify the most likely source element in the reference genome; 79 of these non-reference insertions can be traced back to 20 LINE-1s in the T2T-CHM13 reference

genome. The source elements responsible for more than one non-reference insertion and the location of the non-reference insertions are shown in **Supplementary Fig. 44d**. A LINE-1 insertion on Chromosome 17 is the most likely source element for 12 of these non-reference insertions, and another on Chromosome 16 is the most likely source element for nine non-reference insertions. These findings support the hypothesis that there are only a small number of active LINE-1 loci in each individual. Using BEDTools (Quinlan and Hall 2010), we intersected the non-reference MEIs with the 5'- and 3'-UTRs (untranslated regions), introns, and exons of known protein-coding genes in T2T-CHM13. For Alu elements, LINE-1, and SVA, most of the insertions fall in intergenic regions. Of those that do overlap genic regions, the majority of the insertions intersect introns, with a small number that fall into 5'- or 3'-UTRs. One Alu element retrotransposed into an exon of *PRAMEF4*, a gene involved in cell proliferation, apoptosis, and transcription (**Supplementary Fig. 44e**).

Note S4. Quantifying false positives and negatives in alignment-based *de novo* SNVs

Postzygotic SNVs. As a final filter for false positive postzygotic mutations (PZMs), we used tagging single-nucleotide polymorphisms (tSNPs) to construct the haplotype on which a PZM arose. We first selected tSNPs unique to a parent to determine if a mutation arose on paternal or maternal DNA, and then we further refined our set of tSNPs, eliminating any tSNP that was not heterozygous in the parent. For samples in G3, who have sequenced grandparents in our dataset, we further refined our tSNPs, until we had a set that was unique to the grandparent from whom the haplotype was inherited. Across 36 PZMs in G2 individuals, we assigned all but two to a unique parental haplotype. Across 93 PZMs in G3 individuals, we assigned 88 to unique grandparental haplotypes. For each sample in the direct lineage, we reexamined HiFi data, including every read aligned to a PZM coordinate, regardless of mapping or base quality. If a read matched the tSNP alleles surrounding the PZM coordinate, we determined it was on the same inherited haplotype. We counted the number of reads with the PZM alternate allele from both the inherited and other haplotype present in a sample, as well as on any unphased reads (**Supplementary Fig. 45a**). Finally, we determined that a PZM was an inherited event if a parent and grandparent had any reads with the alternate allele on the inherited haplotype, or if a parent or grandparent had more than one read with the alternate allele on the inherited haplotype. It is worth noting that we did not see any examples of the alternate allele on a different haplotype. In total, we found six PZMs that failed our filters, including three events from one G2 individual, NA12877.

In addition to looking at direct ancestors, we were able to evaluate whether the alternate allele was transmitted to children for 62 PZMs across four samples (G2 n=2; G3 n=2). We determined that a PZM was transmitted if the alternate allele was present on at least

one HiFi read in a child, for a total of 40 PZMs transmitted 120 times. For each transmitted variant, we expected to see the alternate allele on every read attributed to the inherited haplotype, which we saw in 87.5% of transmissions (n=105/120) (**Supplementary Fig. 45b**). However, there were 15 transmissions over 5 PZMs that were not present on every read from the parent.

We also examined a variant's allele balance (AB) across HiFi, Illumina, and ONT (if available) reads in a transmitted child. There were 13 PZMs transmitted 29 times to children with AB consistent across all data types and significantly different from 0.5. However, 7 of those PZMs are transmitted to at least one additional child with AB of 0.5, leaving only 6 PZMs that deviate from expectation. We determined a PZM was a false positive event, likely caused by a recurrent sequencing error, if for every transmission, it was both incompletely linked to the inherited haplotype and had AB different from 0.5 across all sequencing platforms. In total, there were four PZMs that appear to be false positive events, including another two PZMs from NA12877.

We excluded all 10 PZMs that failed to validate either in ancestors or descendants, resulting in a final callset of 119 PZMs. This final set includes 55 PZMs from samples with sequenced children, with a 60% transmission rate.

Between both validation strategies, there were another three PZMs that pass filtration thresholds but have inconsistent transmission profiles across children. These three PZMs may represent likely false positives in our dataset; given that we analyzed 55 PZMs for transmission, we can calculate a false positive rate (FPR) of 5.1%. Compared to the previous study of this family (Sasani et al. 2019), there are no previously identified PZMs that we did not recover, yielding a false negative rate (FNR) of 0%. As such, we can revise our estimate of the PZM rate (mu) to be mu*(1-FPR)/(1-FNR), or 2.23×10^{-9} * (1-0.051)/(1-0.0) = 1.94×10^{-9}.

Germline SNVs. For germline SNVs, we used Element data for the final round of validation. We determined that a DNM was false positive if it was not supported in one of the four sequencing technologies (HiFi, ONT, Illumina, Element) and it had AB < 0.1 in at least one other sequencing technology. For example, a variant with AB of 0.39 in HiFi, 0.05 in ONT, 0.2 in Illumina, and 0 in Element was considered a false positive event. In total, we observed eight such false positives out of 626 DNMs, for an FPR of 1.28%. Like for PZMs, we compared our callset to that of Sasani et al. (2019). After validating their variant calls with our pipeline, there were only four previously discovered DNMs that were absent from our callset. We can calculate our FNR as (false negative)/(true positive + false negative), giving an FNR of 0.64%. We can revise our estimate of the DNM rate (mu) to be mu*(1-FPR)/(1-FNR), or 1.17×10^{-8} * (1-0.0128)/(1-0.0064) =1.16×10^{-8}.

We can combine these germline and postzygotic rates for an overall *de novo* SNV rate of 1.354×10^{-8} mutations per base pair per generation.

Note S5. Haplotype analysis of flanking SNVs to validate recurrent tandem repeat allele transmission

To demonstrate that the flanking informative SNVs are consistent with the genotyped tandem repeat (TR) allele sizes at chr8:2376919-2377075 (T2T-CHM13) in the context of haplotypes across multiple samples, we extracted SNVs from a 4.5 kbp region upstream and downstream of this TR in all reads. Only SNVs with an allele frequency above 0.2 were kept, and their positions were normalized across all samples, categorizing each SNV in each read as observed, unobserved, or not spanned. Per haplotype we then condensed the SNVs in the read data into a single representative vector (**Supplementary Fig. 46**).

Condensing the haplotypes involves computing posterior probabilities for each SNV on a given haplotype using a simple Bayesian model, where the presence of allele 1 is modeled as a binomial distribution with a Beta prior ($\alpha = 0.5$, $\beta = 0.5$). The posterior mean is calculated based on the observed counts of allele 1 and allele 0 across all reads, while accounting for uncovered positions where reads do not span the SNV of interest. We then generate a binary representative vector for each haplotype by thresholding the posterior mean at 0.5, assigning a value of 1 if the posterior mean suggests the presence of allele 1 with a probability of 50% or higher, and 0 otherwise.

Note S6. Details on Chromosome Y analysis

The highly contiguous Y assemblies generated here allowed us to investigate all classes of DNMs for the first time across the entire male-specific Y regions (MSY, i.e., excluding pseudoautosomal regions (PARs)), including the longest heterochromatic block in the human genome, the Yq12 subregion (**Fig. 5a**). Three Y lineages prevalent in populations with European ancestry (Jobling and Tyler-Smith 2003) are carried by the males of the 1463 pedigree: R1b1a-Z302 (n=9, G1-G4), R1b1a-Z326 (n=4, G3-G4), and I1a3a-S2078 (n=1, G1) (**Supplementary Fig. 47, Supplementary Table 12**).

Here, we focused on the nine-member male pedigree carrying the R1b1a-Z302 Y haplogroup (**Fig. 5a, Supplementary Table 12**). DNMs were identified from highly contiguous Verkko Y assemblies generated here for six out of nine males (G1-G3), with the longest Y contigs ranging from 23.1 to 51.2 Mbp. For three males (G1-NA12889, G2-NA12877 and G3-NA12884), representing three generations, the entire MSY (~48.8 Mbp) was contiguously assembled, with assembly breaks only in the PAR(s) (**Supplementary Table 12**). As the T2T-CHM13 Chromosome Y reference (J1a-L816 Y lineage (Rhie et

al. 2023)) differs extensively from the R1b1a-Z302 Y chromosomes, especially in the repetitive regions (**Supplementary Fig. 48**) (Hallast et al. 2023), we decided to use the G1-NA12889 Chromosome Y assembly as a reference for DNM detection. The high quality of the G1-NA12889 Y assembly was confirmed by a comparison to an evolutionarily closely related Y from HG00731 (R1b1a-Z225 Y lineage, time to most recent common ancestor (TMRCA) estimate 5,700 years ago, 95% highest posterior density (HPD) interval = 4,800–6,700 years ago) and to the Y assemblies of his male descendants (**Fig. 5a, Supplementary Figs. 47 and 49**). Additionally, the G1-NA12889 Y assembly is highly similar in size and sequence composition to published assemblies from the R1b lineage Y chromosomes (**Supplementary Table 12**) (Hallast et al. 2023). The accessible *de novo* assembly-based MSY (i.e., excluding PARs and regions flagged as potential misassemblies by either Flagger or NucFreq) for variant calling for the six G1-G3 males ranged from 46.47–48.82 Mbp (mean 48.31 Mbp, median 48.60 Mbp) (**Supplementary Table 13**).

In total, we identified 48 *de novo* SNVs in the MSY across the five G2-G3 males. The majority (45/48) of identified SNVs were located in the Yq12 heterochromatic region, while two were in the Y euchromatic regions and one in the pericentromeric region (**Fig. 5b, Supplementary Table 13**). The average *de novo* SNV rate for euchromatic regions (X-degenerate, X-transposed and ampliconic) across the approximately 22.2 Mbp of accessible length was 1.81×10^{-8} mutations per base per generation (95% CI: 0 – 4.89×10^{-8}, on average 0.4 SNVs/Y transmission), comparable to the *de novo* SNV rate estimates for the same regions from Icelandic patrilineages using Illumina data (2.87×10^{-8} mutations per base per generation, 95% CI: 2.68 – 3.08×10^{-8}) (Helgason et al. 2015). The *de novo* SNV rate estimate for the accessible ~23.4 Mbp of Yq12 (3.86×10^{-7} mutations per base per generation, 95% CI: 3.02 – 4.71×10^{-7}) is >20× higher compared to the euchromatic regions, an average of nine SNVs/Y transmission (**Supplementary Table 13**). However, it is worth noting that a substantial proportion of the SNVs in the highly repetitive Yq12 region might arise from mechanisms like interlocus gene conversion. In fact, 13/45 (29%) had 100% identical matches elsewhere in the respective individual's Y assembly (segments of ±200 bp of flanking sequences around the SNVs were used and the number of identical hits ranged from 2–1000, mean 149, median 3). However, this increased to 25/45 or 56% of Yq12 SNVs if flanking regions of ±25 bp around the SNVs were checked. Approximately 23.2/23.8 Mbp of the Yq12 region in the G1-NA12889 Y was annotated as composing of *DYZ1/Hsat3A6* and *DYZ2/Hsat1B* repeats (12.87 Mbp of *DYZ1* and 10.30 Mbp of *DYZ2* repeats), with their length ratio of 0.56/0.44. The SNVs identified in the Yq12 region follow a similar ratio of 0.52/0.48 (23/44 and 21/44) occurring in *DYZ1/DYZ2* repeats, respectively. The Ti/Tv ratio across the 45 SNVs identified in the Yq12 is low, 0.73.

The average overall *de novo* SNV rate for the ~48.3 Mbp accessible MSY was 1.99×10^{-7} mutations per base per generation (95% CI: 1.59 – 2.39×10^{-7}) or 1.45×10^{-7} (95% CI: 1.12 – 1.79×10^{-7}) if the 13 SNVs in Yq12 likely resulting from gene conversions were excluded (**Supplementary Table 13**).

A total of nine *de novo* indels (<50 bp, homopolymers excluded) were identified from the five males, ranging from 1-3 indels/sample (mean 1.8 events/Y transmission) (**Fig. 5b**). Overall, 8/9 were STRs (5/8 were 4 bp and 3/8 were 5 bp repeat expansions or contractions) while 1/9 was a 1 bp deletion (not in a homopolymer region); 5/9 indels were located in euchromatic regions (all 5 were validated by the Element data), while 4/9 were in the Yq12 regions where short-read data cannot be mapped unambiguously, overall translating to a mutation rate of 3.76×10^{-8} mutations per base per generation in the MSY (95% CI: 8.61×10^{-9} – 6.66×10^{-8}) (**Supplementary Table 13**).

We also identified five *de novo* SVs (≥50 bp), ranging from 2,416 to 4,839 bp in size, an average of one SV per Y transmission (**Fig. 5b**). All identified SVs were located in the Yq12 and result in insertions or deletions of one or two entire *DYZ2* repeat subunits with an average mutation rate of 2.08×10^{-8} mutations per base per generation (95% CI: 0 – 5.20×10^{-8}) (**Supplementary Table 13**). While it has only very recently become possible to investigate the genetic variation at the Yq12 region at base-pair resolution, a similar rate of approximately one SV at this region per approximately 40 Mbp scanned per meiosis was estimated to occur using restriction digestion followed by fractionation by pulse field gel electrophoresis by Neal Mathias (D.Phil. thesis, Mathias, N., 1993, “Y chromosome DNA polymorphisms and human evolution”, University of Oxford).

Due to assembly breaks in the PARs, these regions were not used for DNM detection. However, we aimed at locating the recombination breakpoints at the PARs, which is required for proper disjunction of X and Y chromosomes in male meiosis. No recombination events were identified from the Verkko assemblies in the PAR2s of the five R1b1a-Z302 Y males (one G2 and four G3), while one likely recombination event was present in the PAR1 of G3-NA12884 (**Supplementary Fig. 50**).

Note S7. Evaluation of mutation spectra in unique and repeated regions

To compare the mutation spectra, we subset our DNMs into two groups by intersecting them with different genomic annotations. The first group is composed of the 76 SNVs in SDs (n=52 DNMs, n=24 PZMs over 227.3 Mbp). To make the second group, we took our initial set of *de novo* SNVs and removed all SNVs in SDs, centromeres, acrocentric p-arms, simple repeats, and transposable elements (Alus, LINEs, and SINEs), leaving us with a total of 376 SNVs (n=314 DNMs, n=62 PZMs over 1.65 Gbp) mapping to truly unique regions of the genome. When we use chi-square tests to compare the single-base

substitution spectrum between SNVs in SDs and SNVs in unique regions, no class of mutation rises to statistical significance. The largest difference we observe, however, is a depletion of *de novo* CpG>TpG mutations in SDs, but it is not statistically significant (chi-squared test, p=0.143). We do, however, observe a significant decrease in the *de novo* Ti/Tv ratio in SDs compared to all DNM calls (chi-squared test, p=0.0109) and compared to unique DNM calls (chi-squared test, p=0.012), which is consistent with a recent population genetic-based analysis (Vollger et al. 2023). This decrease in Ti/Tv ratio is not observed for LINE repeats (**Supplementary Fig. 51**).

Note S8. Assembly quality terminology used in this study

AuN - an estimate of assembly contiguity similar to "average" contig length measured by the contig N50 value—the contig length such that at least 50% of the assembly is assembled in contigs of this and longer length. AuN further takes into account a continuous distribution of contig lengths while N50 is single point in this distribution. As a result, AuN is less susceptible to outliers in contig length and represents a more reliable metric. More details about assembly contiguity measures can be found here: https://lh3.github.io/2020/04/08/a-new-metric-on-assembly-contiguity.

Assembly quality value (QV) - QV is an assembly quality metric that reflects the number of k-mers present only in the assembly and not in short Illumina reads for a given sample. The proportion of assembly-only k-mers over the total number of k-mers in the assembly represents the error rate. This error rate is reported as a log-scaled probability of error for the consensus base calls (Phred quality score). For instance, Q30 corresponds to 99.90% assembly accuracy, Q40 to 99.99%, etc.

Misorientation - defined as a region in the assembly whose orientation is in reverse instead of direct orientation. Such errors can be easily seen using Strand-seq data, which has the capability to preserve the orientation of single-stranded DNA (Sanders et al. 2016).

Hamming error rate - represents total number of haplotype-phasing differences between a sample and a reference phasing.

Switch error rate - represents the number of haplotype-phasing switches between a sample and a reference phasing.

Assembly collapse - region in the *de novo* assembly where the assembly algorithm was not able to resolve the correct copy number of paralogous sequences.

Assembly misjoin - point in the *de novo* genome assembly where sequences from distinct parts of the genome are joined together.

Note S9. Evaluation of *de novo* SNV mutations

Autosomal SNVs: All autosomal *de novo* SNVs identified using alignment methods were validated using the same strategy regardless of genomic region, with the exception of SNVs in TRs, for which we applied a minimum AB of 0.05. To validate a DNM call, we examined read data from three orthogonal sequencing technologies: HiFi, ONT, and Illumina. For a given sequencing technology, we examined reads spanning a variant from every sample in the pedigree. Omitting any reads that fell below our mapping quality threshold (59 in HiFi and ONT, 0 in Illumina) or base-quality threshold (10 at the site of the mutation), we counted the number of reads that had the alternate allele, weighting alleles with a base quality >20 (high quality) more than reads with base quality <20 (low quality). Considering each sequencing technology individually, we determined that a variant was truly *de novo* if it was present in a child and absent from its parents, and inherited if a parent had reads with the alternate allele. For HiFi and Illumina, parents were required to have zero high-quality reads with the alternate allele, or up to one low-quality read. Because ONT has a higher error rate, we allowed a parent to have one high-quality read with the alternate allele, or up to two low-quality reads. We combined validations across technologies, determining that a variant was inherited if it looked inherited in any technology. We also excluded any variants that were observed in at least one high-quality HiFi read in any sample of the pedigree not directly descended from the *de novo* sample. True *de novo* events were required to be supported across at least two technologies (n=16 supported only by HiFi and Illumina; n=9 supported only by HiFi and ONT), but the majority of our final SNV callset (n=720/745) were supported by all three. After characterizing germline and postzygotic variants, we further validated PZMs by building haplotypes and tracing their inheritance, as described in **Supplementary Note 4**.

STRs/VNTRs (<50 bp): To assess candidate STR and VNTR DNMs, we took the following approach. Beyond the basic filtering criteria reported in the **Methods**, which eliminated likely false positive DNMs, we validated a subset of DNMs based on **a**) transmission to a subsequent generation, **b**) *de novo* allele consistency with an orthogonal sequencing technology, and **c**) manual inspection using IGV and our own plotting utilities (available at the GitHub repository associated with the manuscript). For criterion (**a**), we asked if candidate DNMs observed in two of the G3 individuals (NA12879 and NA12886) were observed in those individuals' G4 children. If at least one of the G4 children inherited a TR allele matching the *de novo* allele observed in the focal G3 individual, we considered the DNM to be validated by transmission. Importantly, we could only apply validation criterion (**a**) to two of the eight G3 individuals. For criterion (**b**), we asked if candidate STR DNMs with allele lengths ≤120 bp were supported by sequencing evidence from an orthogonal technology (Element AVITI). Details of our orthogonal validation approach can be found in the **Methods** ("Measuring concordance with orthogonal sequencing technology"). For the final criterion (**c**), we manually inspected

candidate VNTR DNMs using both IGV and our own plotting utilities; the latter were used to examine the distribution of CIGAR (insertion and deletion) operations in sequencing reads aligned to candidate DNM loci in all members of a given trio. Candidate VNTR DNMs that were flagged as false positives after manual inspection were removed from the final callset.

Centromeric SNVs: In order to validate centromeric SNVs, we started with a raw SNV callset of 1,789 variant calls generated by parsing CIGAR strings of child-to-parent alignments using the SVbyEye function 'cigar2ranges' (Porubsky et al. 2024). For each variant, we inferred the reference and alternate alleles by examining its location both in the assembly of the child, and in the parent who transmitted the centromere to the child. We aligned both parent and child HiFi and ONT reads to the child's assembly and counted the number of reads with either the reference or alternate allele. Evaluating each technology separately, we considered a variant to be true in HiFi if the child had at least one read (with any mapping quality and base quality) with the alternate allele, and the parent had no reads with the alternate allele. In ONT, we allowed the parent to have up to one read with the alternate allele, because ONT data are more error prone. Once we had evaluated a variant with each type of read data, we combined the results, determining that a variant was true if it had support in both platforms, false positive if it was not supported, and inherited if a parent appeared to have the alternate allele in either HiFi or ONT read data.

Chromosome Y SNVs/indels: The SNVs and indels across the MSY (i.e., excluding the PARs) were called using the G1-NA12889 Y assembly as a reference and all types of variants were then called from the G2 and G3 Y assemblies using Dipcall (Li et al. 2018) (**Methods**). Across the one G2 and four G3 males, Dipcall called a total of 5,609 candidate SNVs and 5,083 indels. The vast majority of the candidate variants (4,927 SNVs and 3,683 indels) were called from G3-NA12886, which has the lowest quality assembly, while for the other samples the number of candidate calls ranged from 102 to 274 (mean of 170 per Y assembly) for SNVs and from 299 to 470 (mean of 350 per Y assembly) for indels, respectively. In order to obtain a confident set of SNVs and indels, the following steps were taken: **i**) The *de novo* assemblies were assessed for misassemblies using Flagger (Liao et al. 2023) and NucFreq (Vollger et al. 2019). Any DNMs overlapping with flagged regions by both or either of the tools were filtered out. **ii**) For SNVs, the final filtered calls were supported by 100% of HiFi reads (i.e., no reads supported the reference allele in offspring or alternative allele in the father) and ONT reads mapped to both the reference and each individual assembly were checked for support. For indels (≤50 bp), homopolymer tracts were excluded from the analysis, while the rest of the calls were validated using the read data (HiFi, ONT, Illumina) as follows: Individual reads mapped to the reference (G1-NA12889 Y assembly) and covering the indel call plus 150 bp of flanking sequence were extracted from all samples using subseq

(https://github.com/EichlerLab/subseq), followed by alignment using MAFFT (Katoh and Standley 2013). All alignments were manually checked and any calls where the HiFi data had two or more reads supporting a reference allele and one or more reads supporting an alternate allele were removed. All final SNV and indel calls were additionally supported (if unique mapping to the region was possible) by both Illumina and Element read data mapped to the reference. **iii**) For all applicable SNVs and indels (i.e., if male offspring was available for the male where the call was made), 100% concordance with the expected transmission through generations was confirmed. Specifically, all *de novo* SNVs (n=10) and indels (n=3) identified in G2-NA12877 were present in the assemblies of his four sons (G3: NA12882, NA12883, NA12884 and NA12886) and supported by the PacBio HiFi read data of his three grandsons (G4: 200101, 200102 and 200105). Similarly, SNVs (n=10) and indels (n=3) identified in G3-NA12886 were supported by the PacBio HiFi read data of his three sons (G4: 200101, 200102 and 200105). **iv**) Additionally, *de novo* SNVs and indels were independently called from the reads mapped to a reference. For this, the HiFi, ONT and Illumina data were mapped to the G1-NA12889 Chromosome Y assembly followed by variant calling using GATK (Poplin et al. 2017) in haploid mode on the aligned HiFi data. Each male was directly compared to his father, selecting variants unique to the son. The SNVs and indels were then validated by examining the father's HiFi, ONT, and Illumina data, excluding any variants present in the parental reads. The final read-based callset was 100% concordant with those called from the assemblies.

NoteS10. Evaluation of SV DNMs

Centromeric SVs: We initially identified 29 candidate SVs in the centromeres (7 between G1 and G2, and 22 between G2 and G3), based on whole-contig alignments between parent and child—note not using a standard human genome reference. We manually checked each of these by aligning the raw ONT and PacBio HiFi reads to the relevant assemblies and assessing for the presence of the SV. We found that 11/29 of these candidate SVs were not supported by the underlying raw reads—FPR of >37% for assembly-based calls. In almost all 11 cases, we found that a small portion of the raw reads also contained the SV, indicating that there was a subpopulation of cells with the SV and a subpopulation without the SV—thus a potentially somatic event. During the genome assembly process, the structure that was most supported by the reads was included in the final assembly, which meant that, in some cases, a false *de novo* SV was included. We were careful in our analysis to check every SV we detected for long-read sequencing support, and the final set of 18 *de novo* centromeric SVs passed this quality control. A subset of these (n=8), where transmission could be assessed, were all confirmed to be transmitted to the next generation. We include a list of all true *de novo* centromeric SVs and their validation metrics in the **Supplementary Table 10**.

VNTR SVs: In the case of *de novo* SVs, we followed a similar procedure. We first collected candidate *de novo* SVs reported based on phased genome assemblies and read-based variant calling. In the case of read-based SV calls from Sawfish, we searched for support in phased genome assemblies by constructing a multiple sequence alignment (MSA) of each deemed *de novo* SV. Out of a total of 658 *de novo* SV candidates, we marked 12 variants as likely valid (FPR >98%). All of these were marked as various VNTRs and were subsequently vetted by the TRGT callset. We evaluated candidate SV DNMs at TR loci by visually inspecting the distribution of CIGAR operations in HiFi reads aligned to each locus (for more information about the process we used to extract CIGAR information from high-quality reads, see **Methods** section "Measuring concordance with orthogonal sequencing technology"). For each member of a trio, we plotted the net number of CIGAR operations in every read aligned to a VNTR locus harboring a candidate DNM. We included examples of the resulting images in the Platinum-Pedigree-Consortium GitHub repository associated with the manuscript (https://github.com/Platinum-Pedigree-Consortium/ppc-trs/tree/main/hq_sv_images). By examining the distributions of CIGAR operations in each member of the trio, we evaluated whether the child's sequencing reads contained evidence of a *de novo* allele length absent from both parents.

Chromosome Y SVs: The SVs across the MSY were called from G2 and G3 Y assemblies using Dipcall with the G1-NA12889 Y assembly as a reference (**Methods**). Across the one G2 and four G3 males, Dipcall called a total of 70 candidate SVs. Similar to the SNVs and indels, a higher number of raw SV calls were made from G3-NA12886 Y assembly (n=30), while 16 candidate SVs were called from G2-NA12877 and 7 to 10 SVs from the remaining three samples (G3-NA12882, G3-NA12883 and G3-NA12884). Any *de novo* variants overlapping with flagged regions by Flagger and/or NucFreq were filtered out. For all SV calls, HiFi read depth for reference and alternative alleles was visualized and SVs in regions showing high levels of read depth variation coinciding with clusters of SNVs with >10% of reads supporting an alternative allele removed. Additionally, HiFi and ONT reads that mapped to both the reference and individual assemblies were checked for support. For all applicable SVs (i.e., if male offspring was available), 100% concordance with the expected transmission through generations was confirmed. Specifically, all *de novo* SVs (n=3) identified in G2-NA12877 were present in the assemblies of his four sons (G3: NA12882, NA12883, NA12884 and NA12886) and supported by the PacBio HiFi read data of his three grandsons (G4: 200101, 200102 and 200105). Similarly, the *de novo* SV identified in G3-NA12886 was supported by the PacBio HiFi read data of his three sons (G4: 200101, 200102 and 200105).

Other SVs: All predicted *de novo* SVs outside of centromeres and VNTRs were evaluated by Verkko as well as hifiasm (UL) assemblies. We did this by extracting a sequence around the SV by adding two times the size of the SV on each side. We extracted the

sequence from a G3 individual and corresponding G2 parents. Next, we constructed the MSA and visually checked if the predicted SV is visible in both Verkko and hifiasm (UL) assemblies. For assembly-based *de novo* SV candidates, we considered the PGGB (Garrison et al. 2024) (pangenome SV callset) and PAV (Audano et al. 2019) callsets with a reported initial number of 84 and 75 *de novo* candidates, respectively. Of these, all but one assembly-based *de novo* candidate were rejected. Thus, >99% were false positives. The only *de novo* variant reported by phased assemblies is the SVA insertion described in this paper. In the case of read-based *de novo* SV candidates, we considered the Sawfish callset of the initial number of 658 of putative *de novo* SVs (see VNTR section above for more details). With respect to GRCh38, we also considered other read-based *de novo* SV candidates reported by Sniffles (Smolka et al. 2024) (n=77) and PBSV (n=45). However, none of these passed our assembly-based validation. We note that our strict requirement of having all *de novo* SVs fully assembled might lead to false negative calls and, as such, our reported numbers likely represent a lower bound. The complete list of detected *de novo* SVs is reported in **Supplementary Table 10**.

We observed systematic errors in both hifiasm and Verkko assemblies. Most of these were false insertions, which in the case of hifiasm were often composed of a long homopolymer stretch (**Supplementary Fig. 52**). We, however, note that hifiasm and Verkko are still in active development and some of these errors are being addressed in newer software versions.

SUPPLEMENTARY TABLES

All supplementary tables are available as individual .xlsx files.

SUPPLEMENTARY FIGURES

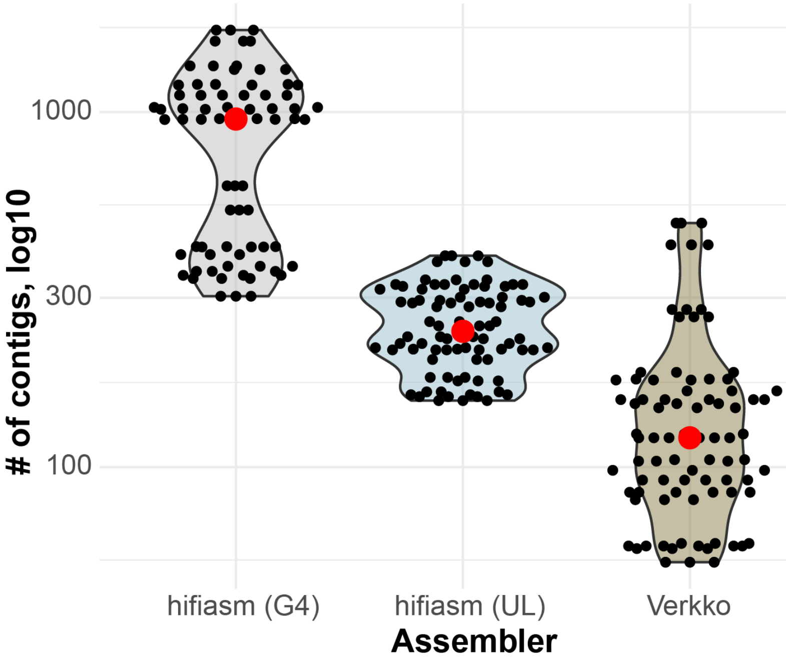

Figure S1: Evaluation of assembly contiguity.
Distribution of the total contig counts assembled using Verkko (brown), hifiasm (UL) (light blue), and assemblies of G4 samples using hifiasm (light gray). Red dots define the median value.

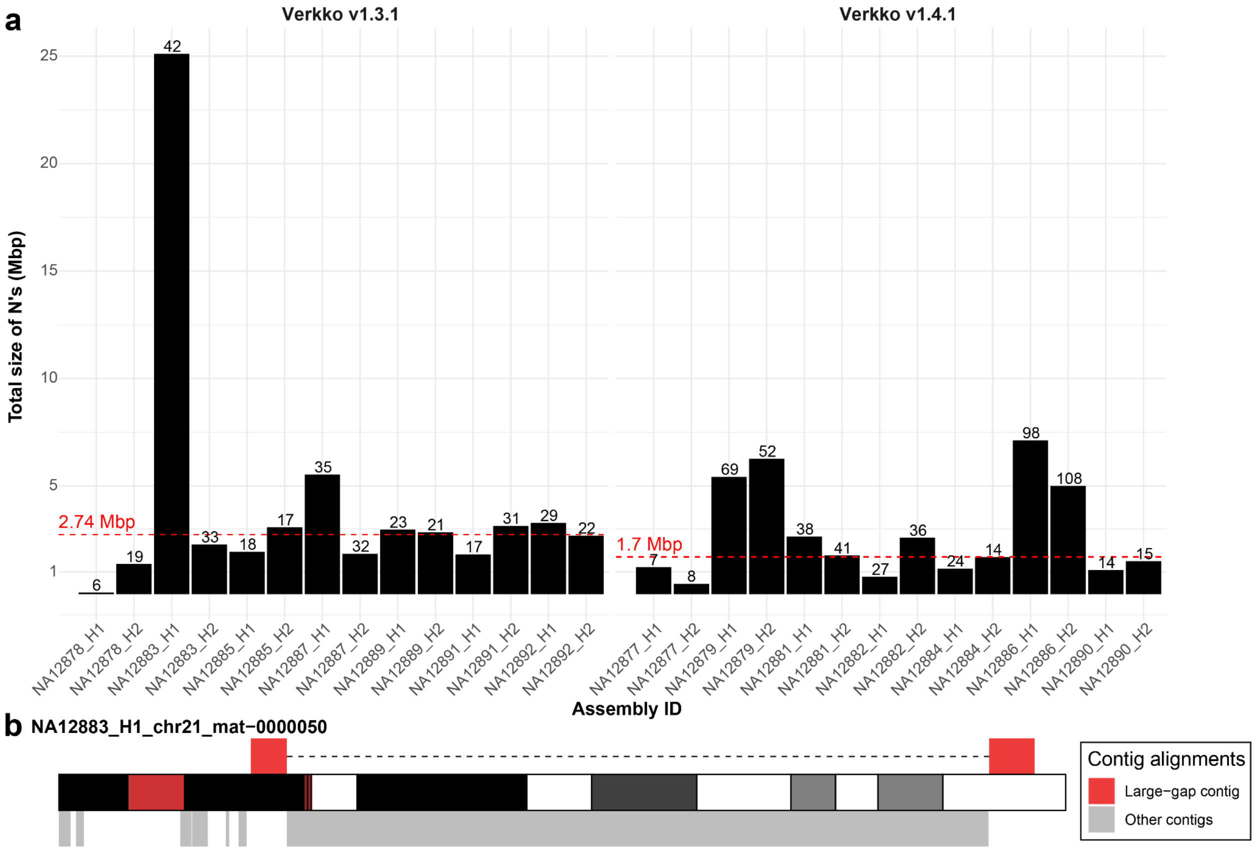

Figure S2: Evaluation of gaps in scaffolded Verkko assemblies.

Verkko-scaffolded contigs contain a total of 896 gaps corresponding to an estimated gap size of 2.4 Mbp per assembled human genome haplotype of G1-G3 samples.

a) A barplot showing total length of N's detected in scaffolds reported by Verkko. On top of each bar is the number of separate strings of N's detected in each phased Verkko assembly. The median length of all N's strings in each assembly is shown as a horizontal red dashed line separately for Verkko versions 1.3.1 and 1.4.1. **b**) Explanation for an observed outlier of excess on N's in NA12883_H1 assembly caused by a single contig that maps at opposite ends of Chromosome 21 where space in between was filled by string of N's by Verkko. This is likely a bug in the Verkko (v1.3.1) pipeline as this contig was supposed to be divided into two different contigs.

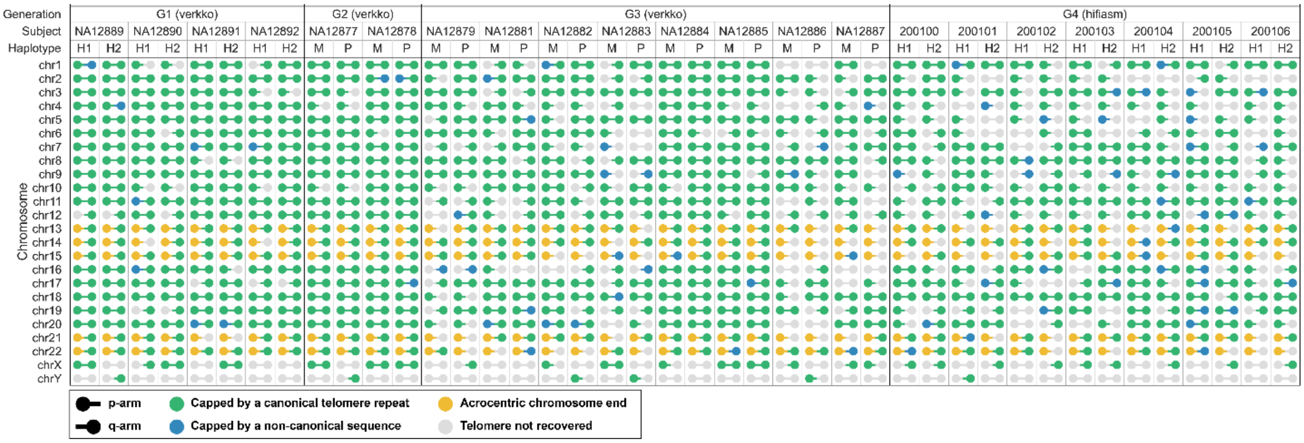

Figure S3: Telomere completeness in phased genome assemblies.
Evaluation of telomere recovery across G1-G4 assemblies and all chromosomes. *p* and *q* arms are marked separately. See **Extended Data Figure 1d** for the visualization of the chromosomes that are spanned by a single T2T contig in Verkko assemblies for G1-G3; out of all hifiasm assemblies for G4, only chr9 of 200101 haplotype 1 is verifiably contiguous due to the absence of ultra-long ONT reads that have not been generated for the G4 samples.
Note: As expected, acrocentric chromosomes (13, 14, 15, 21 and 22) and chromosomes with secondary constrictions (chromosomes 1qh, 9qh and 16qh) composed of multiple megabase pairs of human satellite sequences (HSAT 1-3) were almost never completely assembled

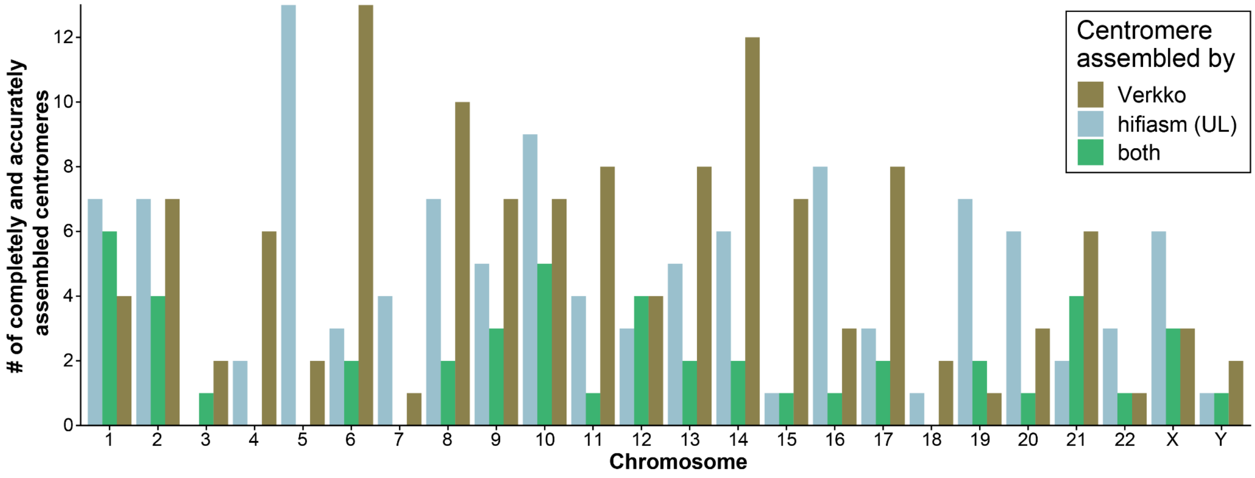

Figure S4: Centromere completeness in phased genome assemblies.
A barplot showing the number of completely and accurately assembled centromeres by Verkko (brown), hifiasm (UL) (light blue), or both assemblers (green). This plot highlights certain chromosomes (e.g., 5, 6, 17 and 19) as being preferentially assembled by different assembly algorithms.
Note: Verkko, for example, assembled 175/644 centromeres (27.2%) accurately, while hifiasm (UL) assembled 161/644 centromeres (25.0%) accurately. Only 48/644 centromeres (7.5%) were completely and accurately assembled by both Verkko and hifiasm (UL). Thus, by merging complete centromeres generated by both assemblers, we create a nonredundant list of 288 completely and accurately assembled centromeres.

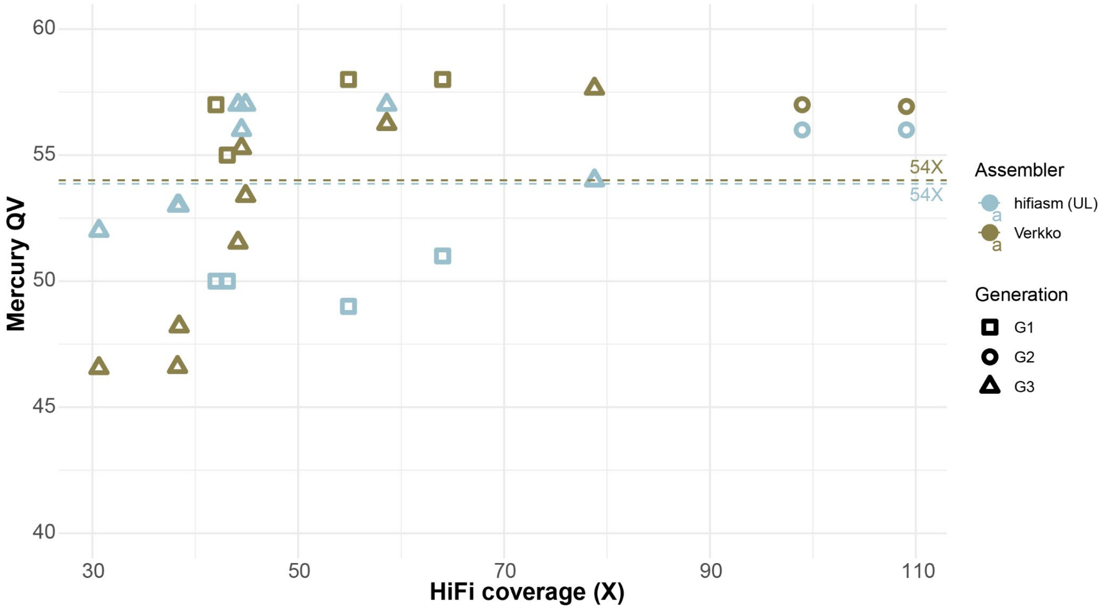

Figure S5: Evaluation of assembly quality.

Distribution of assembly quality values (QV) for assemblies of G2 and G3 using either Verkko (brown) or hifiasm (UL) (light blue) assembler. Mean QV across haploid assemblies for a given assembler is shown as horizontal dashed line.

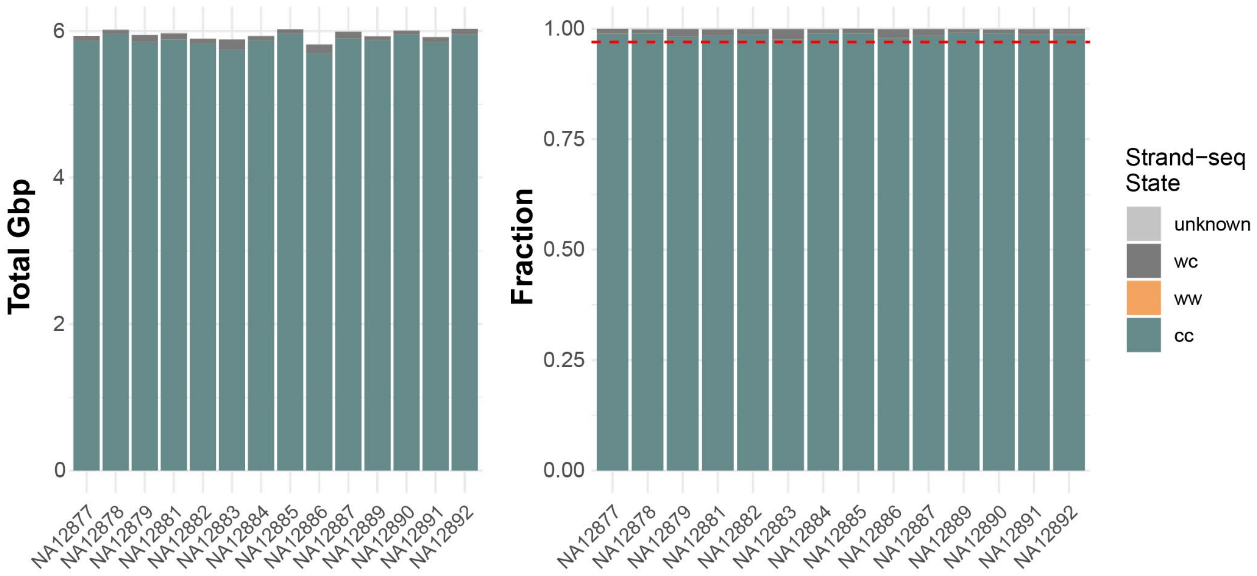

Figure S6: Evaluation of misoriented regions with Strand-seq.

Left: Total number of base pairs (per diploid genome) genotyped as ‘cc’ which means they agree with Strand-seq reference orientation. On the other hand, regions genotyped as ‘ww’ point to possible misorientations. Regions genotyped as ‘wc’ are either caused by the presence of heterozygous inversions or low mappability regions for short Strand-seq reads. ‘Unknown’ region could not be reliably genotyped, most likely due to spurious mapping of short Strand-seq reads.

Right: The same as the left plot but shown as a fraction of bases of each Strand-seq state. Red horizontal dashed line marks 0.97 value.

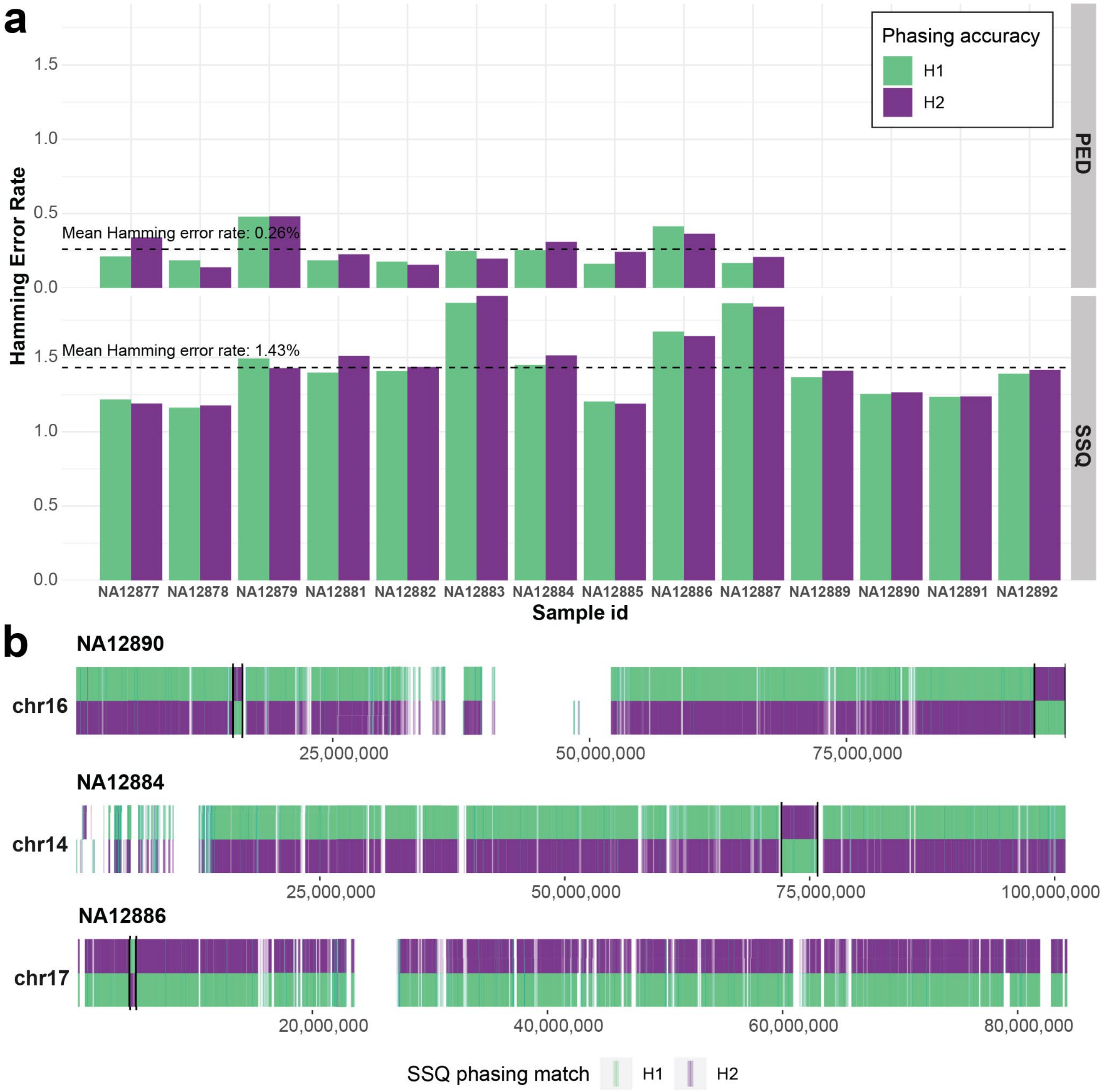

Figure S7: Evaluation of assembly phasing accuracy.

a) A barplot showing the level of phasing disagreement of phased genome assemblies with respect to pedigree-based phasing (PED) and Strand-seq (SSQ) phasing. Phased variants are compared with respect to the GRCh38 reference. Hamming error rate is reported separately for haplotype1 (H1 - green) and haplotype2 (H2 - purple) for each sample from G2-G3. **b**) A distribution of heterozygous single-nucleotide variants (SNVs) along three chromosomes (14, 16 and 17) where large-scale haplotype switch errors were detected in Verkko assemblies. Here, variants detected with respect to the T2T-CHM13 reference are compared between assembly-based SNV callset (obtained from PAV) and Strand-seq (SSQ)-based phasing. Each variant is colored green if it matches haplotype 1 (H1) or purple if it matches haplotype 2 (H2) of the Strand-seq-based phasing. Extended phasing switch errors are visible as regions where H1 changes to match H2 or vice versa (**Supplementary Table 4**). We note that we fixed these four haplotype switch errors in our assembly-based variant callsets to avoid biases in subsequent analysis.

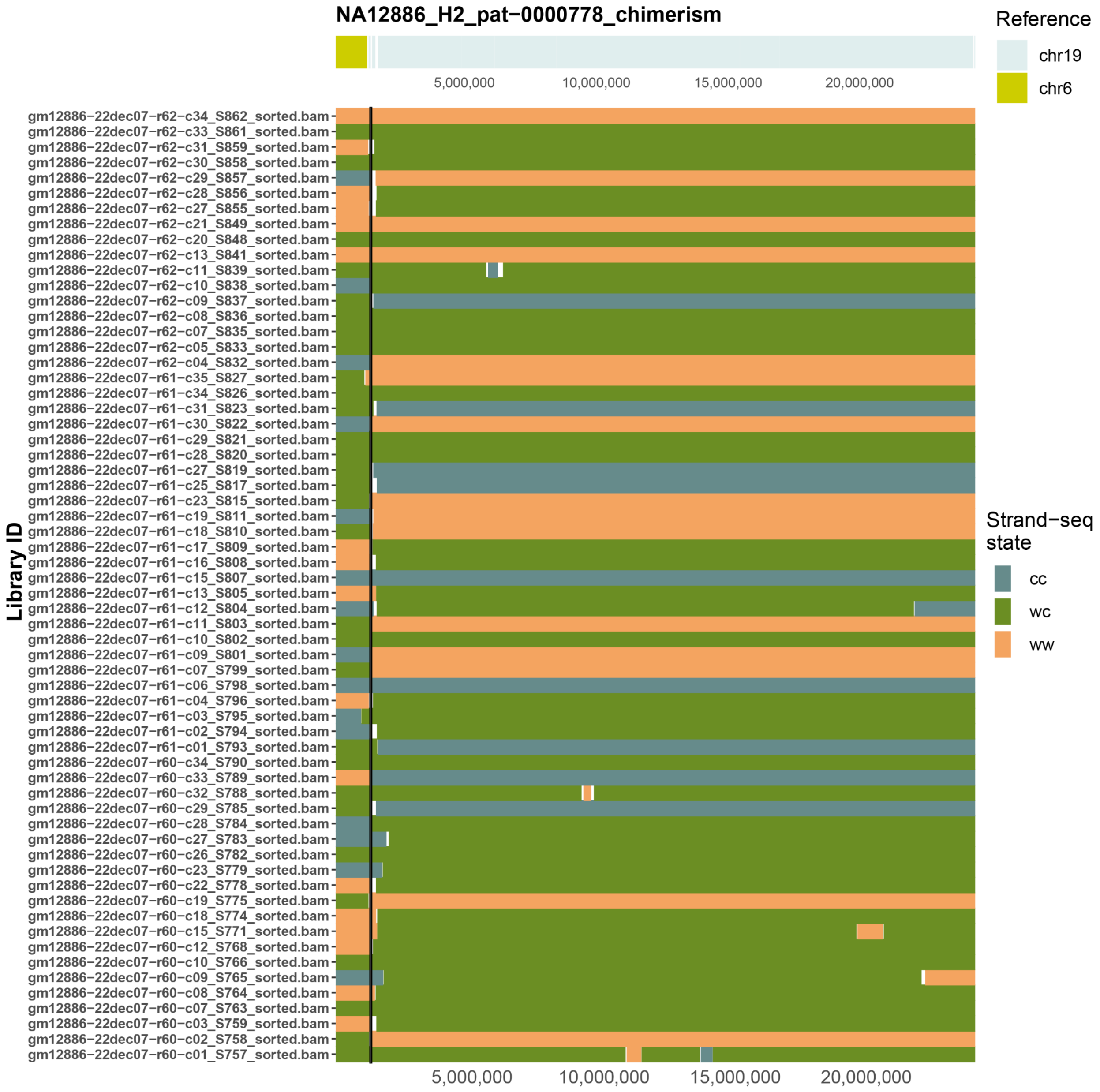

Figure S8: Chimeric contig in Verkko assembly.

Top: Partial mapping of NA12886 paternal contig 'pat-0000778' to Chromosomes 6 and 19 of the T2T-CHM13 reference.

Bottom: Horizontal colored bars show Strand-seq strand states for individual single-cell libraries. Each region is genotyped as WC: Watson-Crick (green); WW: Watson-Watson (orange); CC: Crick-Crick (blue). Observed recurrent strand-state change is indicative of a genome misassembly. Recurrent strand-state change in a paternal contig in sample NA12886 points to contig chimerism as parts of the contig can be aligned to both Chromosomes 6 and 19. The point of recurrent strand-state change supporting misassembly is highlighted by the vertical line. All chimeric contigs detected in Verkko or hifiasm assemblies are reported in **Supplementary Table 4**.

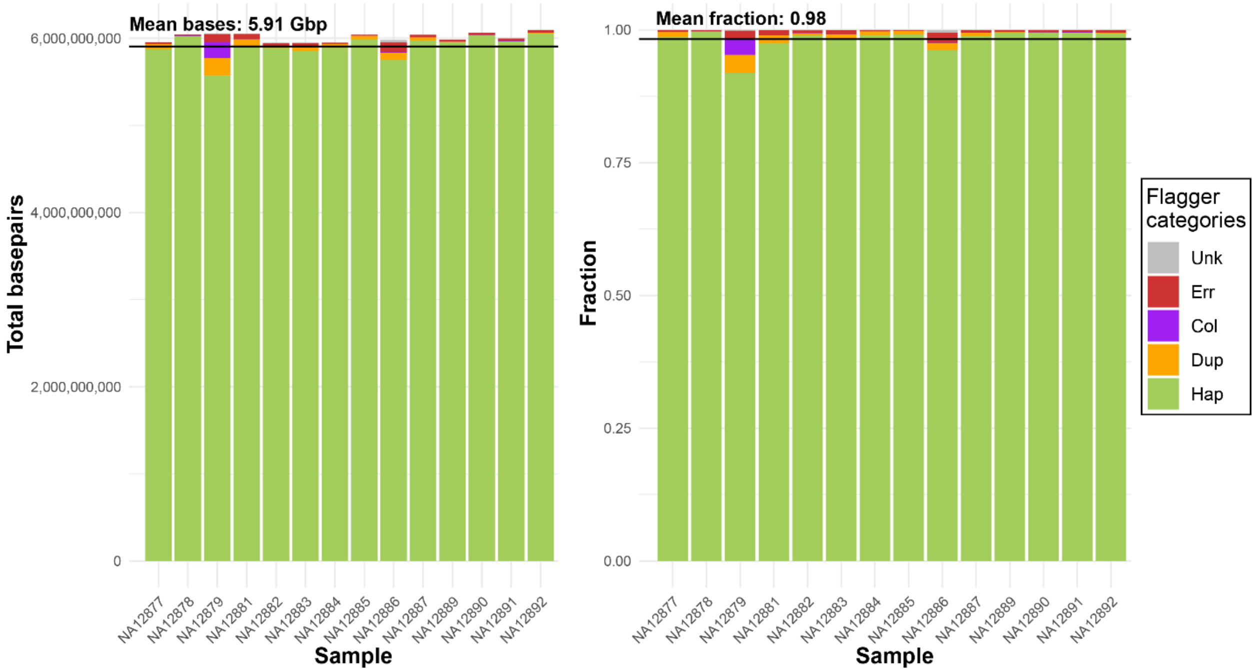

Figure S9: Flagger summary of possible problematic regions in phased assemblies.

Flagger categorizes the genome assembly into segments based on the mapping of HiFi (high-fidelity) reads back to the assembly. A short definition of these categories follows: **Err** (Erroneous, red) A low-read coverage segment that could be either a misjoin or a region that needs polishing. **Dup** (Duplicated, orange) Likely a false duplication of another region in the genome. **Hap** (Haploid, green) A genomic segment that is correctly assembled and has the expected read coverage. **Col** (Collapsed, purple) Two or more highly similar haplotypes are collapsed into this block. **Unk** (Unknown, gray) A segment that could not be confidently assigned.

Note: Flagger reports on average >98% (5.91 Gbp) of each phased assembly being assembled at the correct copy number with one outlier sample (G3-NA12879) with an excess of potential collapses. However, this observation is not supported by the alternate validation tool NucFreq, suggesting that this particular sample may be subject to a less uniform sequence coverage (**Supplementary Fig. 10**).

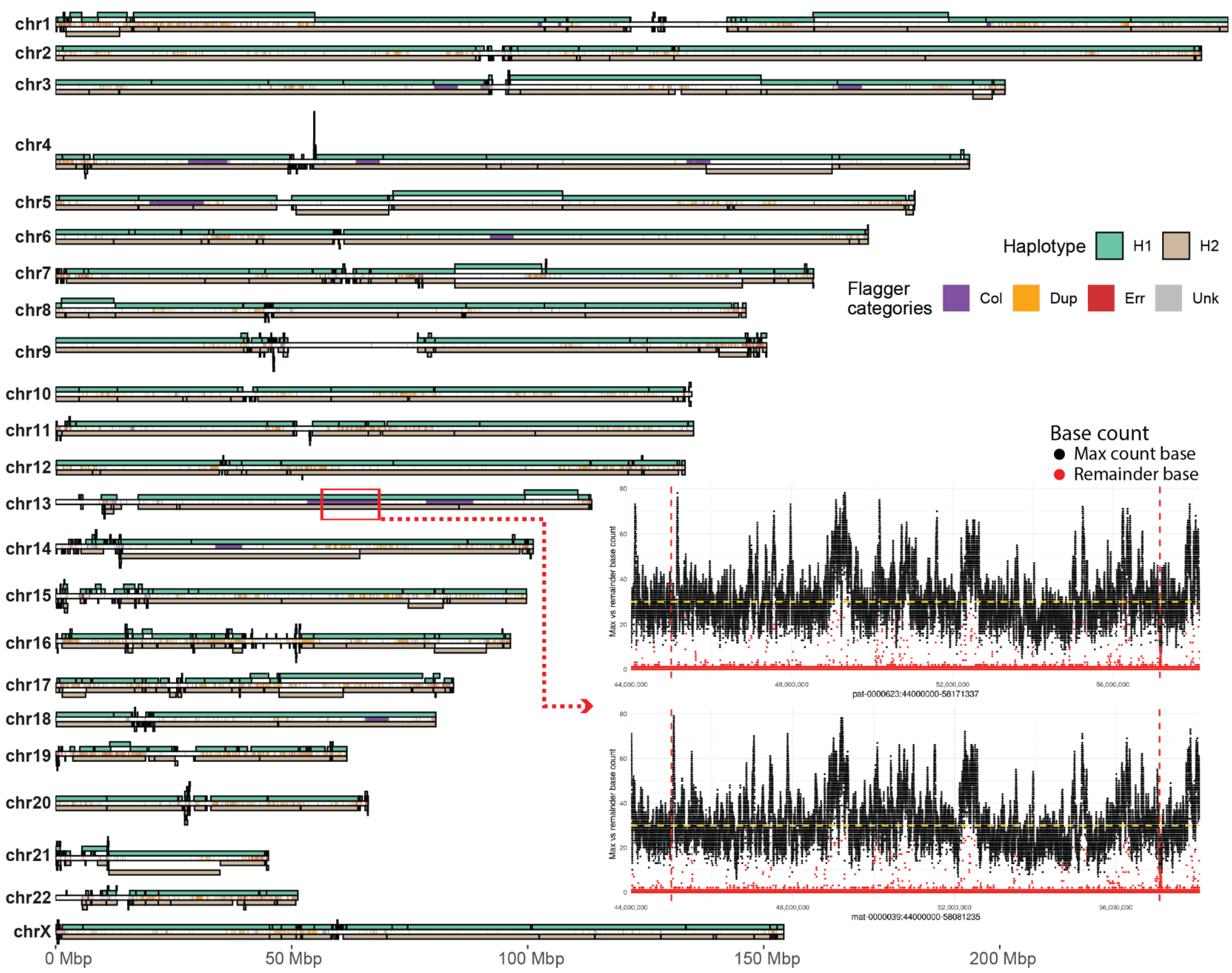

Figure S10: Flagger evaluation of NA12879.

An ideogram showing alignments of phased assembly to the T2T-CHM13 reference with haplotype 1 contigs (H1 - light green) being shown above the chromosome midline and haplotype 2 (H2 - light brown) contigs below. Positions of regions reported by Flagger as collapsed ('Col' - purple), duplicated ('Dup' - orange) or erroneous ('Err' - red) (see **Supplementary Fig. 9** legend for more details) are shown at the midline of each chromosome.

Inset: A NucFreq visualization of ~12 Mbp region (red rectangle highlight in top plot) reported as collapsed by Flagger for both paternal and maternal haplotype assembly. The horizontal yellow dashed line shows median coverage and vertical red lines show region boundaries reported by Flagger. Black dots show the count of the most abundant base ('max') at a given position while red dots show the count of the second most frequent base ('remainder'). Low frequencies of the second most frequent base along the whole region suggest that a given region is correctly assembled and observed coverage fluctuations inherent to the HiFi data analyzed here.

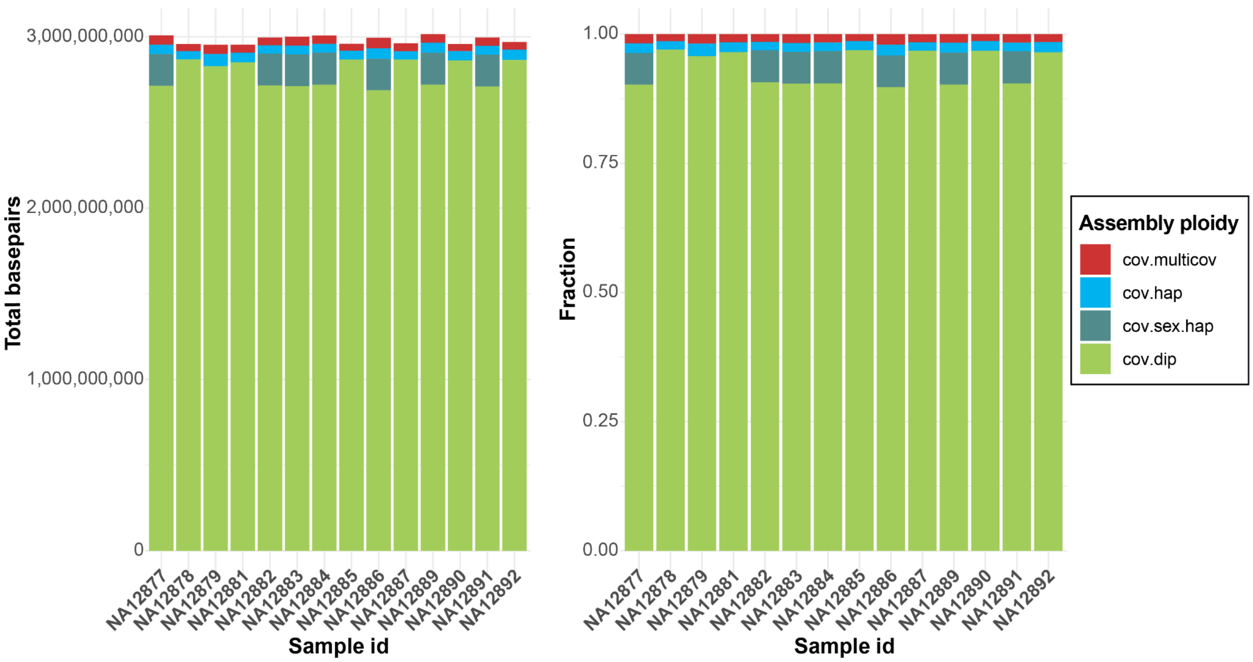

Figure S11: Ploidy summary of aligned Verkko genome assemblies to T2T-CHM13 reference.

A stacked barplot showing the total number of base pairs (left) and proportion of a number of base pairs (right) reported as having exactly single alignment per haploid assembly, thus being a stable diploid region (‘cov.dip’ - green). In males, sex chromosomes are expected to be single copy, thus having a single alignment (‘cov.sex.hap’ - teal). Regions that have reported more than a single alignment per haploid assembly are marked as multi-coverage regions (‘cov.multicov’ - red) while those that are missing an alignment in either haplotype are marked as haploid (‘cov.hap’ - light blue). We report that on average ~97% (2.88 Gbp) of each phased assembly aligns to the reference at expected diploid copy number.

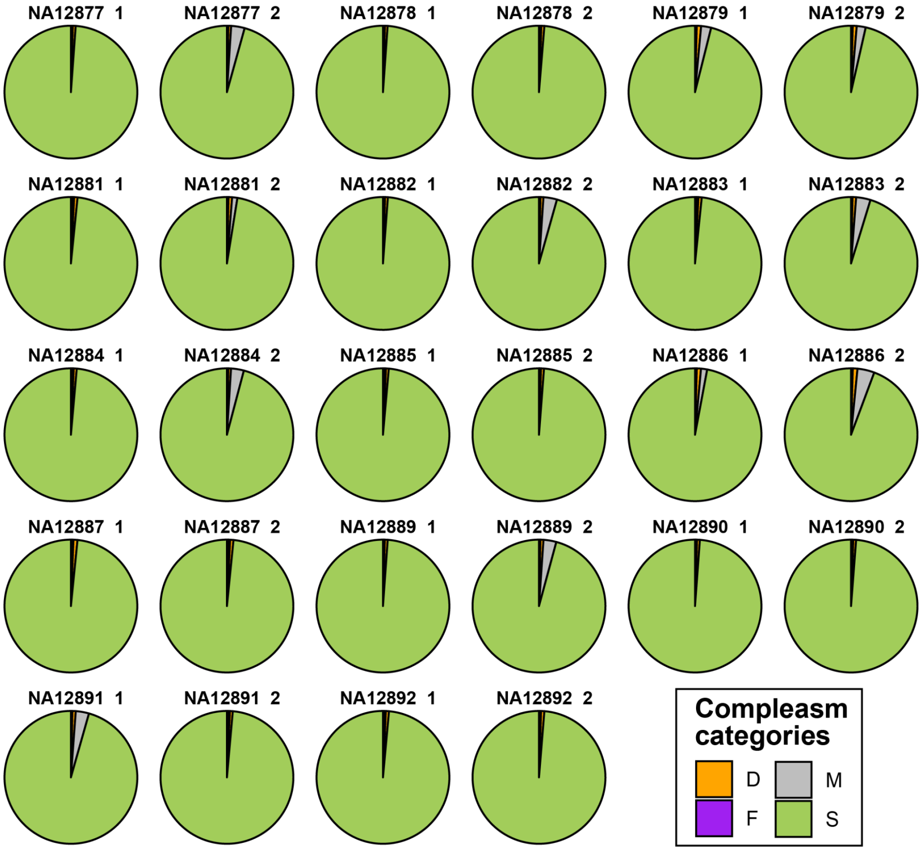

Figure S12: Gene completeness assessment using compleasm.

Each pie chart shows a fraction of correctly assembled single-copy genes for each phased assembly (1 - haplotype1, 2 - haplotype 2). See below for an explanation of each compleasm (Huang and Li 2023) (miniBusco) category:

- S (Single-Copy Complete Genes): The BUSCO genes that can be entirely aligned in the assembly, with only one copy present.
- D (Duplicated Complete Genes): The BUSCO genes that can be completely aligned in the assembly, with more than one copy present.
- F (Fragmented Genes, subclass 1): The BUSCO genes where only a portion of the gene is present in the assembly, and the rest of the gene cannot be aligned.
- M (Missing Genes): The BUSCO genes with no alignment present in the assembly.

Overall, we report >98% completeness of single-copy genes.

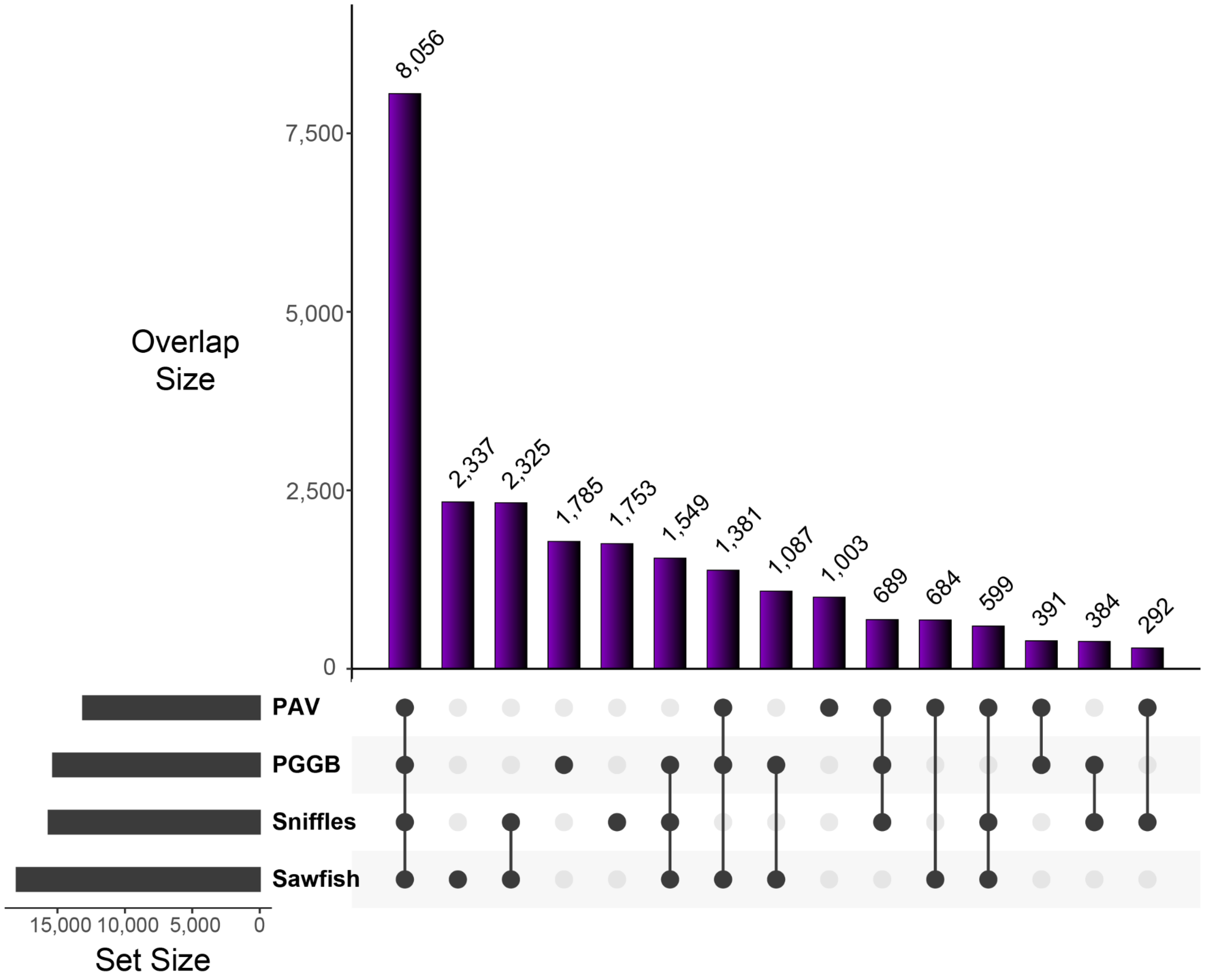

Figure S13. The agreement among different SV callers in the curated truth set.
The UpSet plot depicts the intersection of pedigree consistent structural variant (SV) callsets, excluding tandem repeats. The bars on the right indicate the size of each SV callset, while the central bar chart displays the counts of overlapping sets. On average, each SV call is supported by 2.5 (average) of the four methods, and 61% of SV calls are supported by more than one method. The agreement between methods varied by type: deletions had the highest overlap supported by 2.9 (average) callers, insertions supported by 2.3 (average) callers, and inversions supported by 1.1 (average) callers.

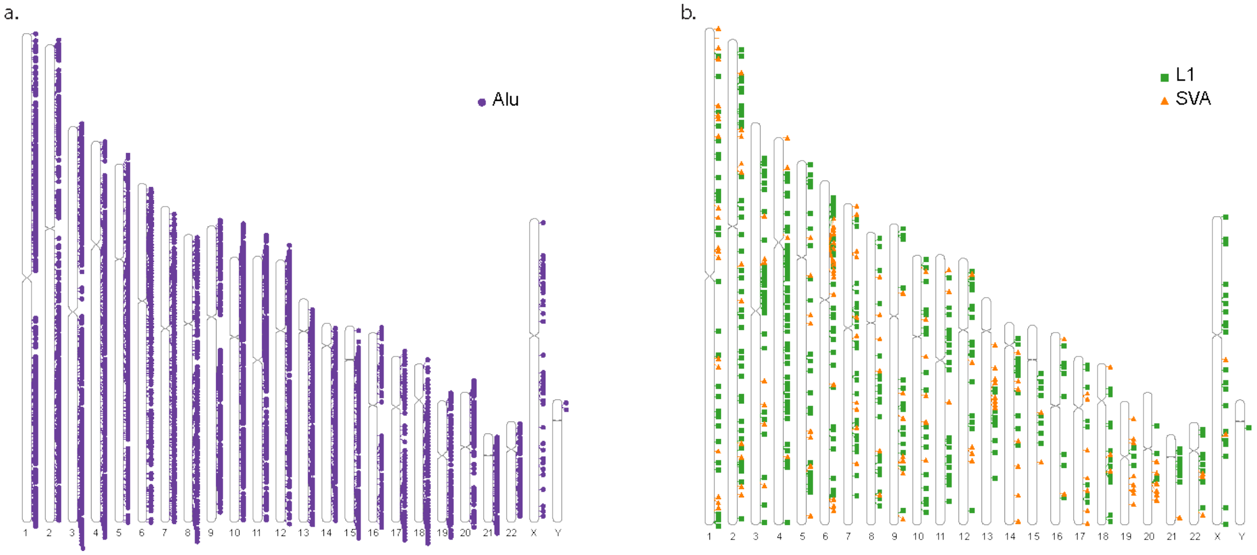

Figure S14: Non-reference mobile element insertion (MEI) analysis.
Genome-wide distribution of MEI SVs. We identify 2,161 Alu insertions, 398 LINE-1 insertions, and 149 SINE-VNTR-Alu (SVA) retrotransposon insertions. Only Alu elements >260 bp are shown here. We identify 112 LINE-1 insertions of either full-length or near full-length (at least 5,500 bp) and 123 SVA insertions of at least 2,000 bp (**Supplementary Table 6**). **a**) Ideogram of non-reference Alu element insertions in T2T-CHM13. **b**) Ideogram of non-reference LINE-1 and SVA insertions in T2T-CHM13. These plots include both full-length and shorter MEI events.

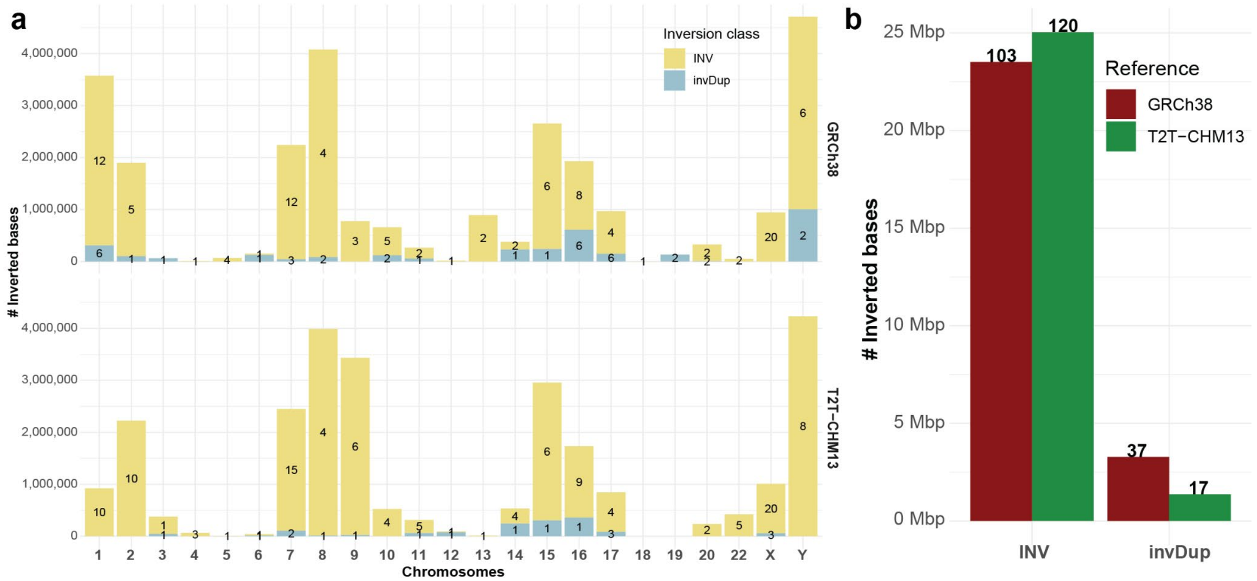

Figure S15: Strand-seq-based inversion callset.

a) A barplot showing the total number of inverted bases with respect to a reference genome (top - GRCh38, bottom - T2T-CHM13). The number in the middle of each stacked bar reports the count of nonredundant inversions contributing to the total count of inverted bases. Inversion calls are stratified based on their class (simple inversions - INV, inverted duplications - invDup). **b**) A barplot showing the total number of simple inversions (INV) and inverted duplications (invDup) with respect to a reference genome (GRCh38 - red, T2T-CHM13 - green).

Note: Median inversion (n=120) and inverted duplication (n=17) size with respect to T2T-CHM13 is ~53 kbp and ~41 kbp, respectively.

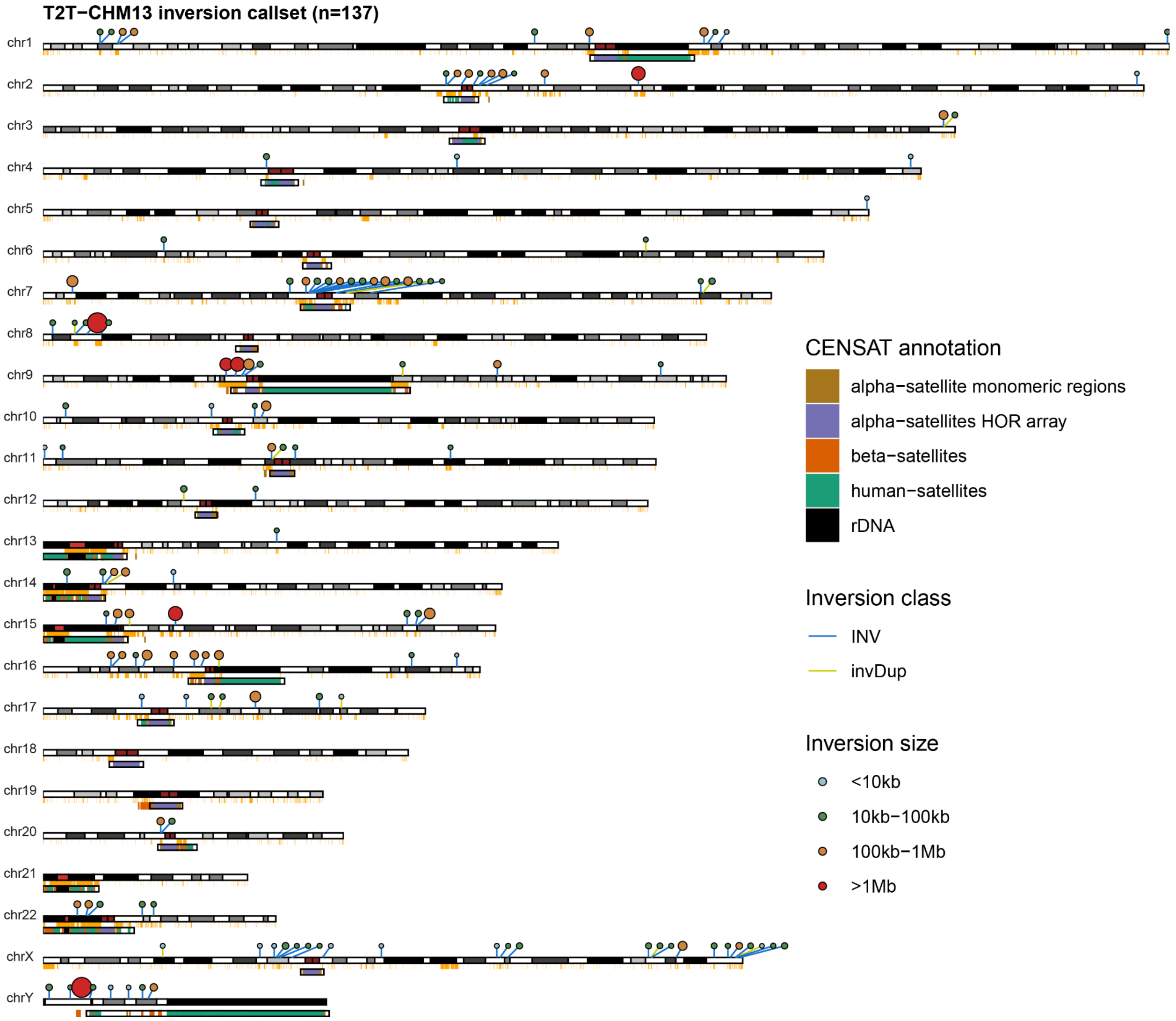

Figure S16: Strand-seq inversion callset with respect to T2T-CHM13.
An ideogram showing the distribution of simple inversions (INV - blue link, n=120) and inverted duplications (invDup - yellow link, n=17) with respect to the T2T-CHM13 reference. The size and color of each dot reflects the inversion size category. Below each chromosome ideogram there is a segmental duplication (SD) annotation (orange) and centromeric satellite annotation (CENSAT).

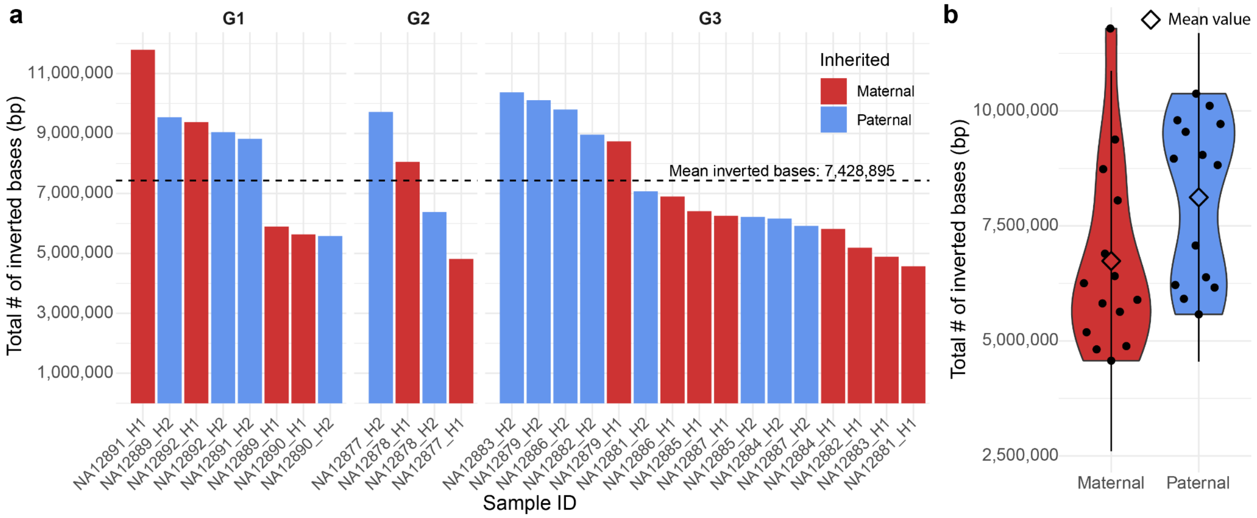

Figure S17: Inverted bases per haploid genome and per generation.
a) A barplot showing the total number of inverted bases per maternal (H1 - maternal; red) and paternal (H2 - paternal; blue) haplotype across G1-G3 samples. The dashed line shows the mean number of inverted bases across all samples and haplotypes. **b**) A violin plot showing the distribution of inverted bases per maternal (H1 - maternal; red) and paternal (H2 - paternal; blue) homolog. The diamond point indicates the mean value for each distribution. There are on average 6.7 Mbp and 8.1 Mbp of inverted bases per maternal and paternal homologs, respectively.

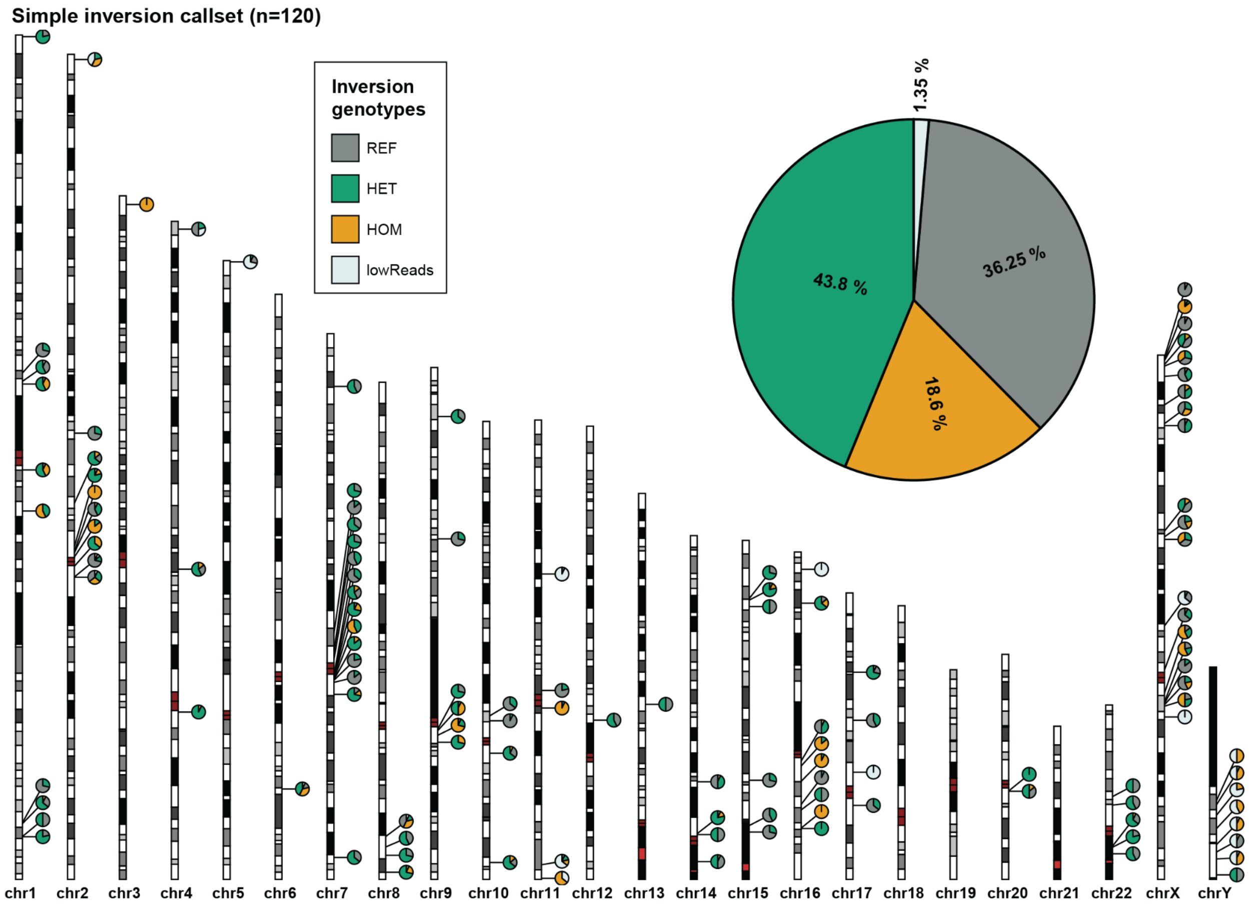

Figure S18: Summary of Strand-seq inversion genotypes.

An ideogram showing the inversion genotype ('REF' - reference orientation, 'HET' - heterozygous inversion, 'HOM' - homozygous inversion, and 'lowReads' - not enough Strand-seq reads to genotype) proportions across G1-G3 for simple inversions (n=120). Each pie chart represents a single inverted region. The large inset pie chart shows the overall proportion of homozygous and heterozygous inversions in this family.

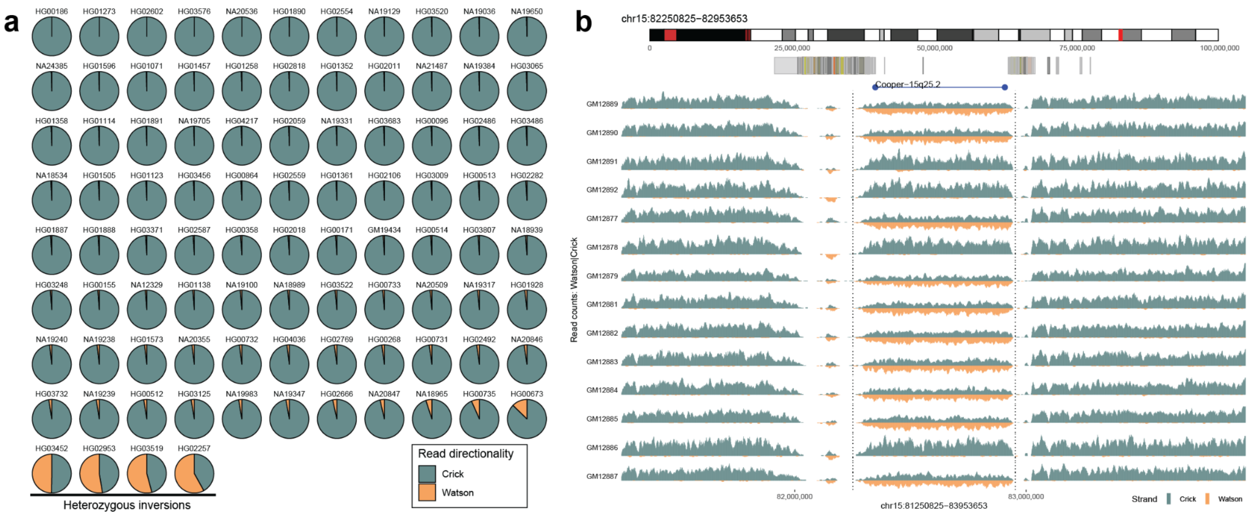

Figure S19: Rare inversion at 15q25.2-25.3.

a) A multiple pie chart plot showing the proportions of Crick (plus read - teal) and Watson (minus reads - orange) aligned to the region of interest across 1000 Genomes Project samples (n=92). **b**) The read-coverage profiles of Strand-seq data over the region of interest summarized as binned (bin size: 10 kbp, step size: 1 kbp) read counts represented as bars above (teal; Crick read counts) and below (orange; Watson read counts) the midline. Dotted lines highlight the inverted region with respect to T2T-CHM13. In this region, equal coverage of Watson and Crick counts represents a heterozygous inversion as only one homologue is inverted with respect to the reference while reads aligned only in Watson orientation represent a homozygous inversion. Above is the chromosome ideogram with the region of interest highlighted in red followed by SD annotation and diseases-associated copy number variant region for Cooper syndrome (Cooper et al. 2011).

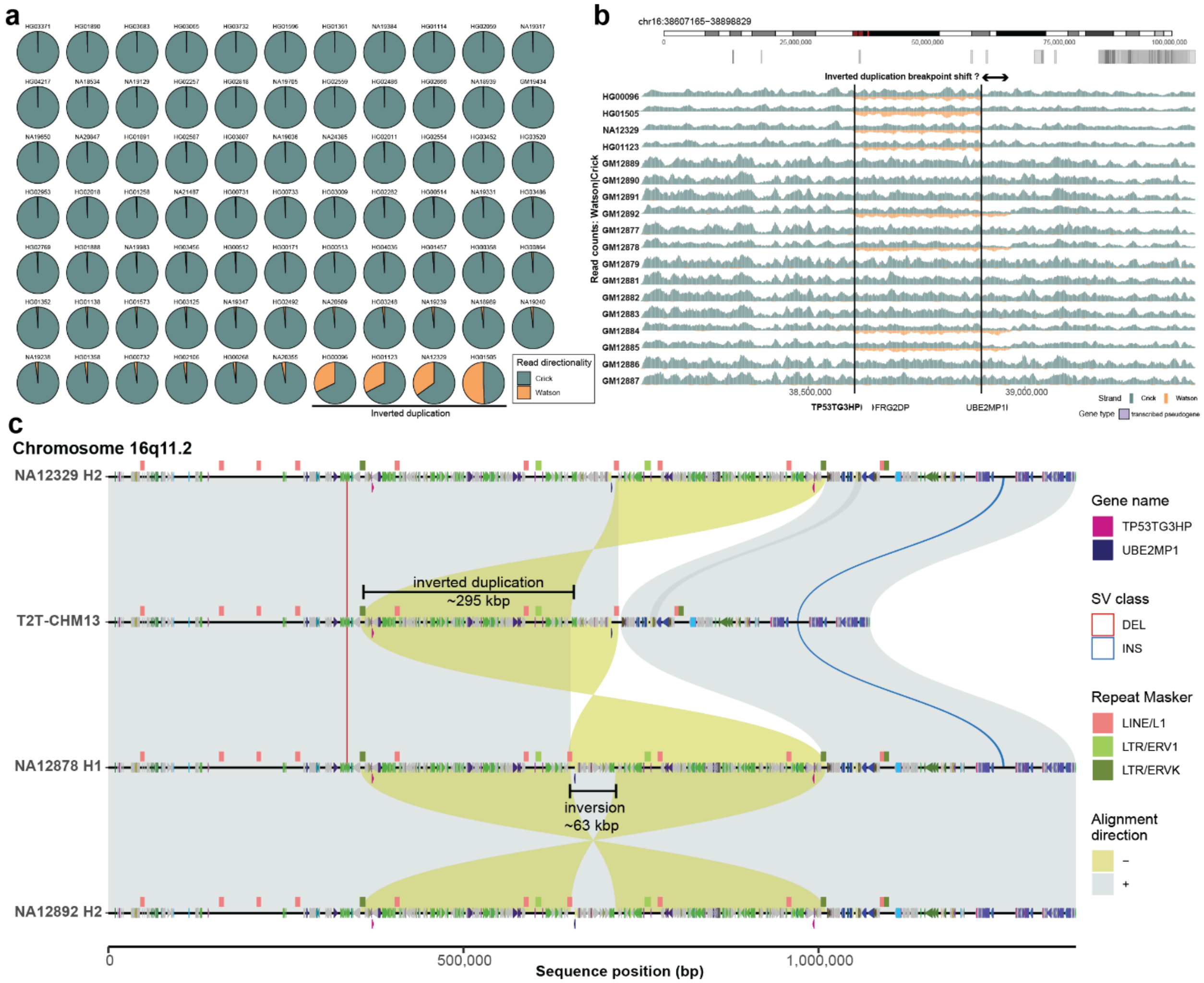

Figure S20: Rare, inverted duplication at 16q11.2.

a) A multiple pie chart plot showing the proportions of Crick (plus read - teal) and Watson (minus reads - orange) aligned to the region of interest across 1000 Genomes Project samples (n=92). **b**) The read-coverage profiles of Strand-seq data over the region of interest (T2T-CHM13; chr16:38250451-39322284) summarized as binned (bin size: 10 kbp, step size: 1 kbp) read counts represented as bars above (teal; Crick read counts) and below (orange; Watson read counts) the midline. Vertical solid lines highlight the duplicated and inverted regions with respect to T2T-CHM13 supported by roughly equal coverage of Watson and Crick read counts with respect to the reference without this inverted duplication. Above is the chromosome ideogram with the region of interest highlighted in red followed by SD annotation. **c**) A visualization of syntenic relationships between three fully assembled haplotypes (NA12329-H2, G2-NA12878-H1, and G1-12892-H2) and the T2T-CHM13 reference based on minimap2 alignments. Direct (+, forward) alignments are shown in gray and reverse (-) alignments in yellow. On top of each haplotype we show the DupMasker (Jiang et al. 2008) annotation as colored arrowheads pointing forward or backward for direct and reverse oriented segments, respectively. Also, there is a RepeatMasker annotation. Structural variants (SVs; ≥50 bp) are shown as red (DEL - deletion) and blue (INS - insertion) lines between aligned haplotypes.

Note: We find that the region (~63 kbp) between this inverted duplication is specifically inverted in this family, which changes the orientation of *UBE2MP1*—a pseudogene whose expression was recently linked to negative outcomes in hepatocellular carcinoma patients (Hao et al. 2022).

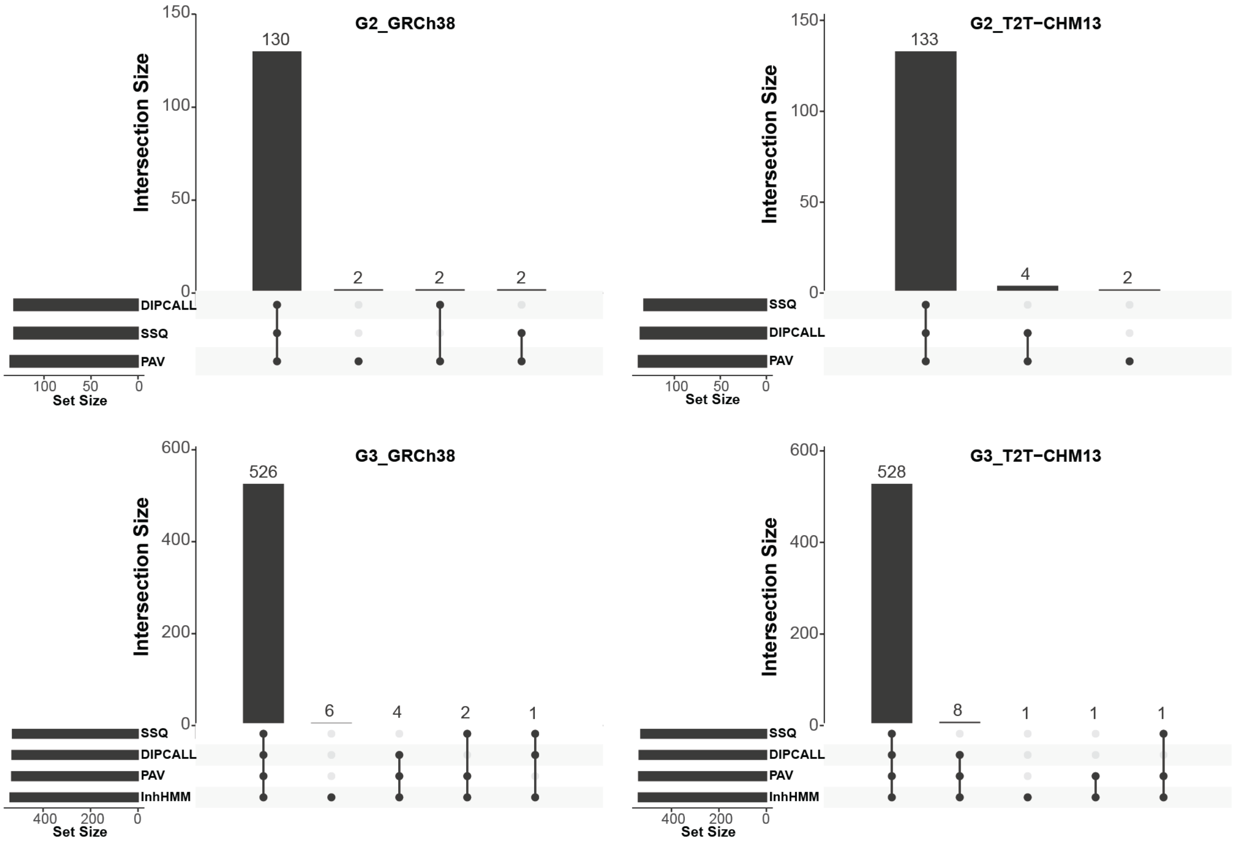

Figure S21: Recombination breakpoint overlap.

Each UpSet plot summarizes an overlap between the reference recombination map with respect to supporting orthogonal datasets. In the case of G3, the reference recombination map is based on inheritance vectors (InhHMM) while supporting datasets are based on maps defined with the help of phased genome assemblies (PAV, Dipcall) and Strand-seq (SSQ). In the case of G2, the reference recombination map is based on phased genome assemblies (specifically based on PAV) while supporting datasets are based on maps defined with help of phased genome assemblies (Dipcall) and Strand-seq (SSQ).

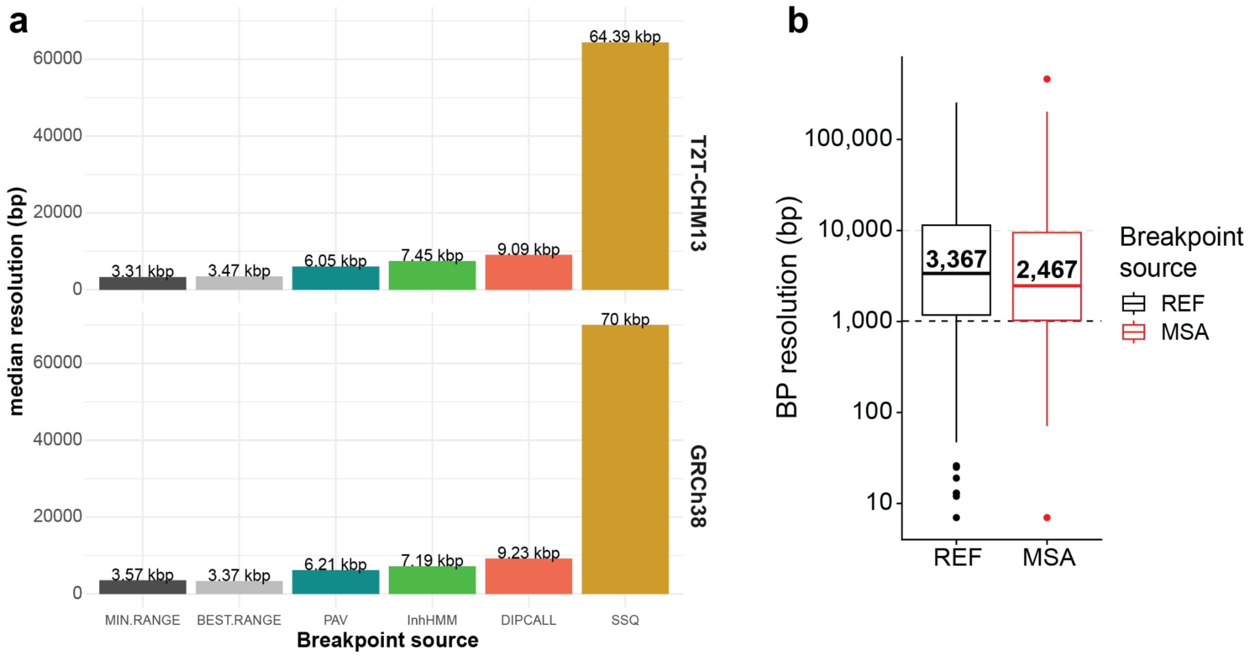

Figure S22: Recombination breakpoint resolution of G3.

a) A barplot showing a median breakpoint resolution separately for recombination maps reported by different orthogonal datasets (assembly-based: PAV and Dipcall; inheritance-vector-based: InhHMM and Strand-seq-based: SSQ). There is also a so-called 'best.range'—the narrowest breakpoint range among all datasets that overlap the reference breakpoint. Lastly, we report the median value for so-called 'min.range'—the range with the highest coverage among all datasets. **b**) Summary of refined recombination breakpoints (n=487) using phased genome assemblies and multiple sequence alignment (MSA) between sequence extracted from the parental and inherited homolog in the child as opposed to breakpoints defined with respect to a single reference (REF: T2T-CHM13). Boxes represent interquartile range (IQR), including median line; whiskers extend to 25% − 1.5 × IQR and 75% + 1.5 × IQR; outliers are shown as dots.

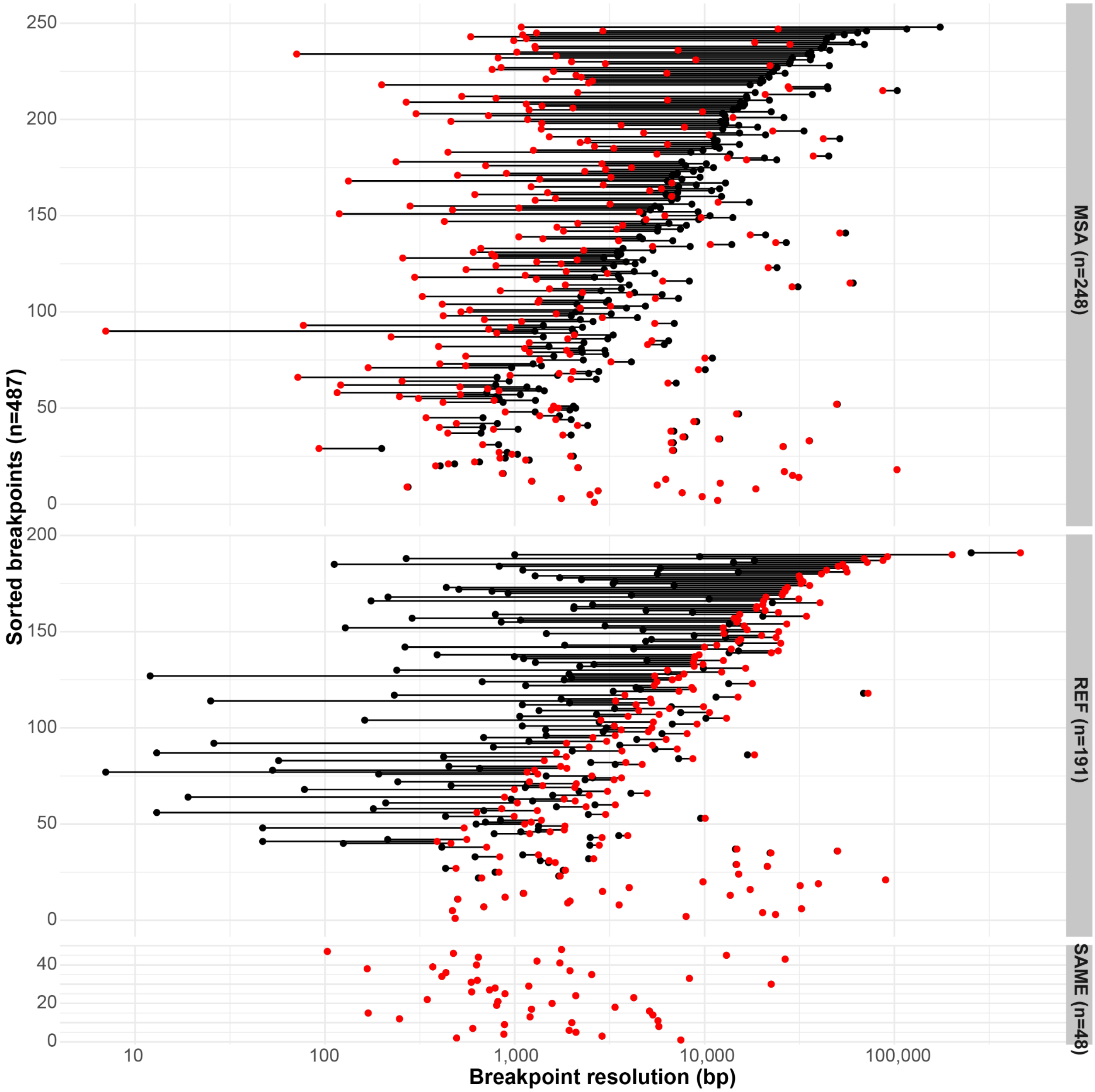

Figure S23: Recombination breakpoint refinement using phased genome assemblies.

Size distribution of refined recombination breakpoints (n=487) from the total of 539 recombination breakpoints in G3 (with respect to T2T-CHM13). Resolution of original reference breakpoints are marked by black points while the refined breakpoints are marked by red points. Original and refined breakpoints are connected by horizontal lines. Breakpoints with improved resolution after multiple sequence alignment (MSA) analysis are on top (MSA, n=248). Breakpoints in the expanded recombination region after MSA analysis are shown in the middle (REF, n=191). Last, breakpoints with largely unchanged resolution are shown at the bottom (SAME, n=48).

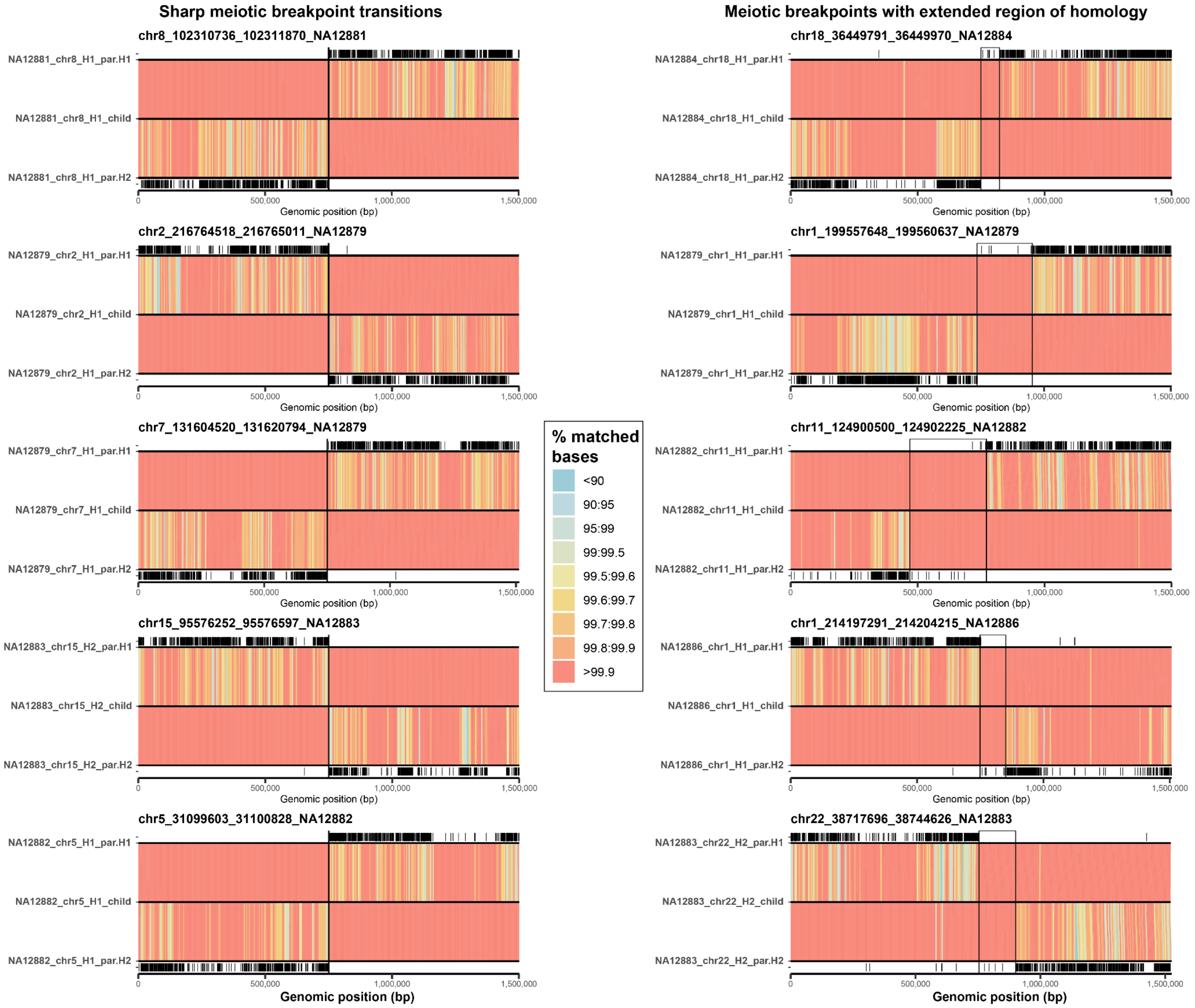

Figure S24: Sharp and wide transition at recombination breakpoints.

Alignments between the parental and child haplotypes are binned into 5 kbp long bins and colored based on the percentage of matched bases. Black tick marks show the positions of mismatches between parental and child haplotypes. We present five examples of sharp recombination breakpoint transitions (left) as well as meiotic breakpoints with extended regions of homology (right). Extended regions of homology at the recombination breakpoints are highlighted by black rectangles.

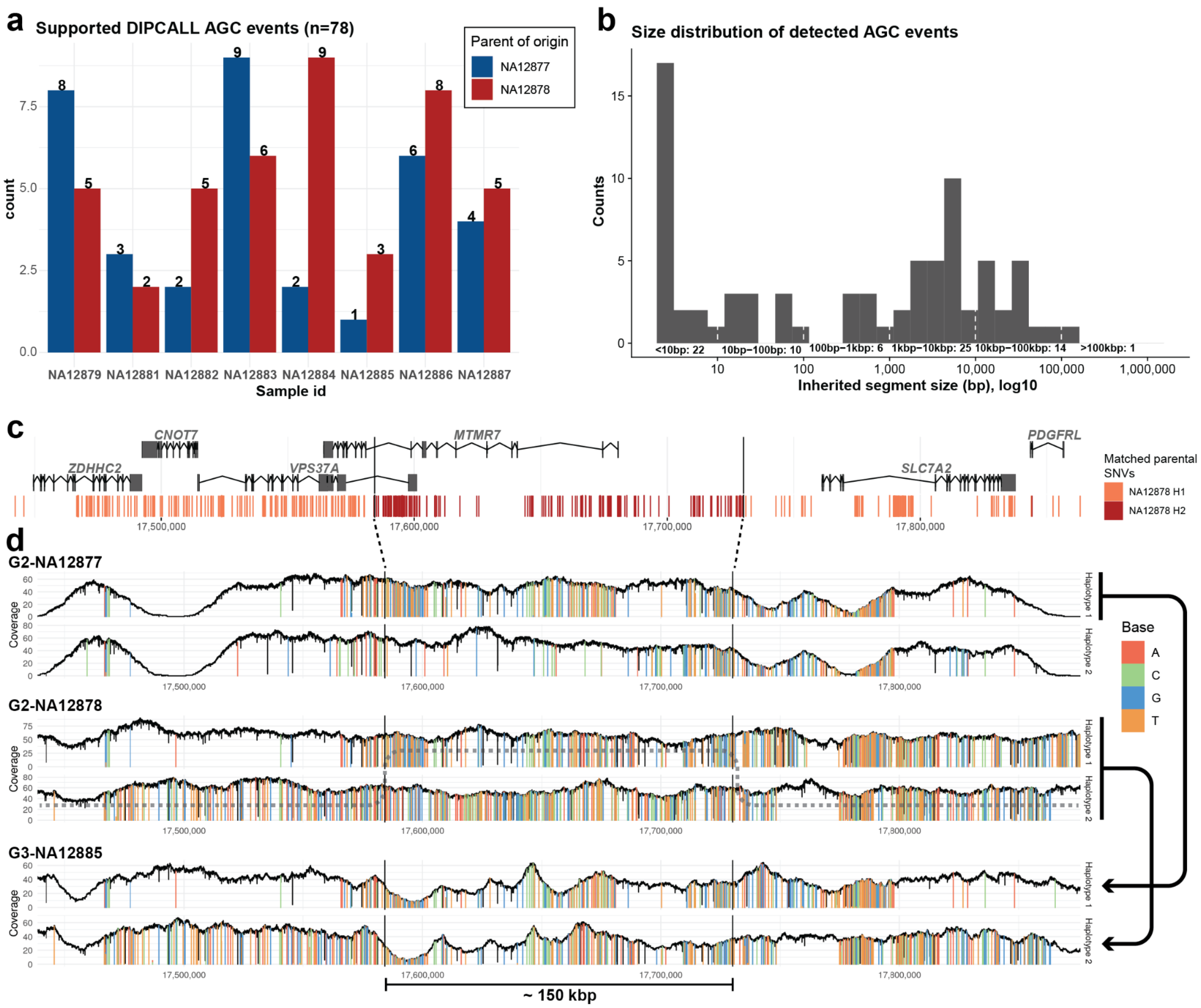

Figure S25: Putative allelic gene conversion events (T2T-CHM13).

a) A barplot showing the total number of allelic gene conversion (AGC) events detected in G2 and G3 samples stratified by parental identity of inherited homologs (red - maternal, blue - paternal). **b**) Size distribution of observed AGC events from panel a. **c**) Overview of protein-coding genes overlapping an example AGC region on Chromosome 8 (chr8:17584336-17730132). Below there are visualized SNVs from sample G3-NA12885 colored based on their match to the inherited parental (G2-NA12878) haplotype 1 (light red) or haplotype 2 (dark red). **d**) A mismatch pattern of phased HiFi reads with respect to the T2T-CHM13 reference for both haplotypes from parent 1 (G2-NA12877), parent 2 (G2-NA12878), and a G3 sample (NA12885). Arrows on the right side of the plot show what homolog from the parent was inherited based on the matching pattern of observed mismatches. The child's haplotype two is composed of a mixture of alleles from NA12878, which is in line with allelic gene conversion or eventually two double-strand breaks and resolved as cross-over.

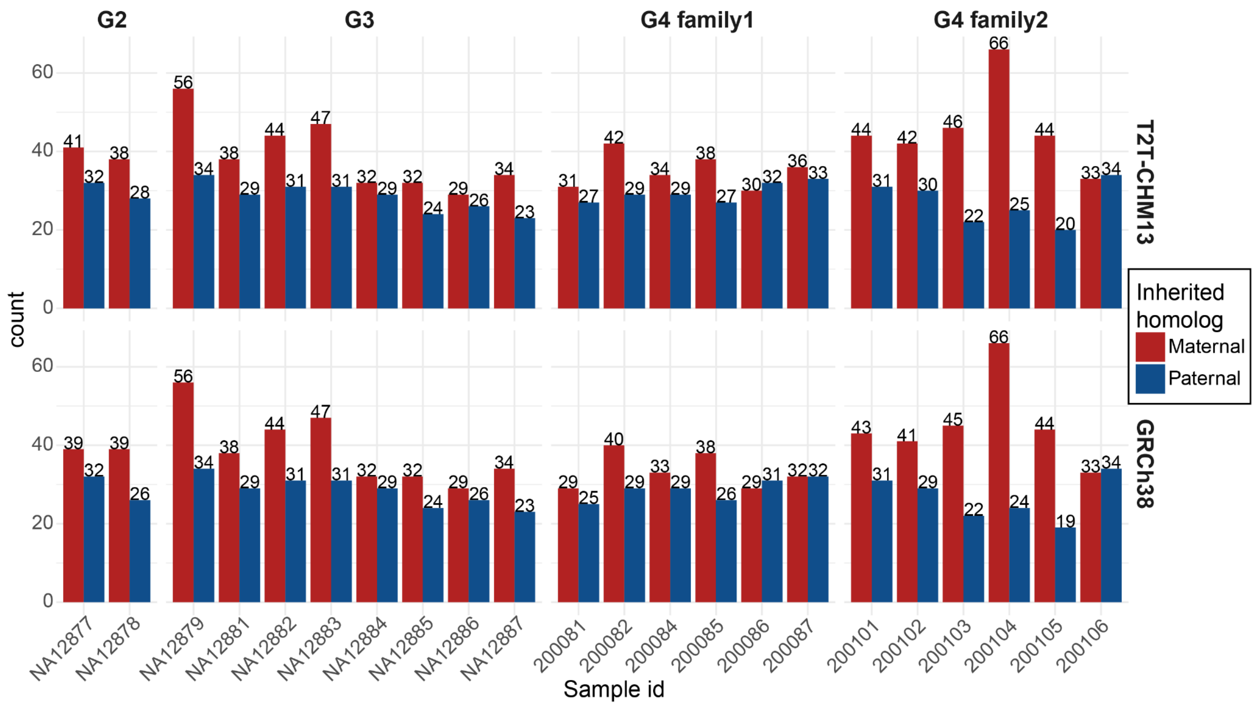

Figure S26: Male and female recombination breakpoints per sample and generation.

A barplot showing the total number of recombination breakpoints detected in each sample (G2-G4) colored by inherited homologs (red colors - maternal, blue colors - paternal). There are a total of 1,503 and 1,479 breakpoints with respect to T2T-CHM13 (top) and GRCh38 (bottom) reference genome, respectively (**Supplementary Table 8**).

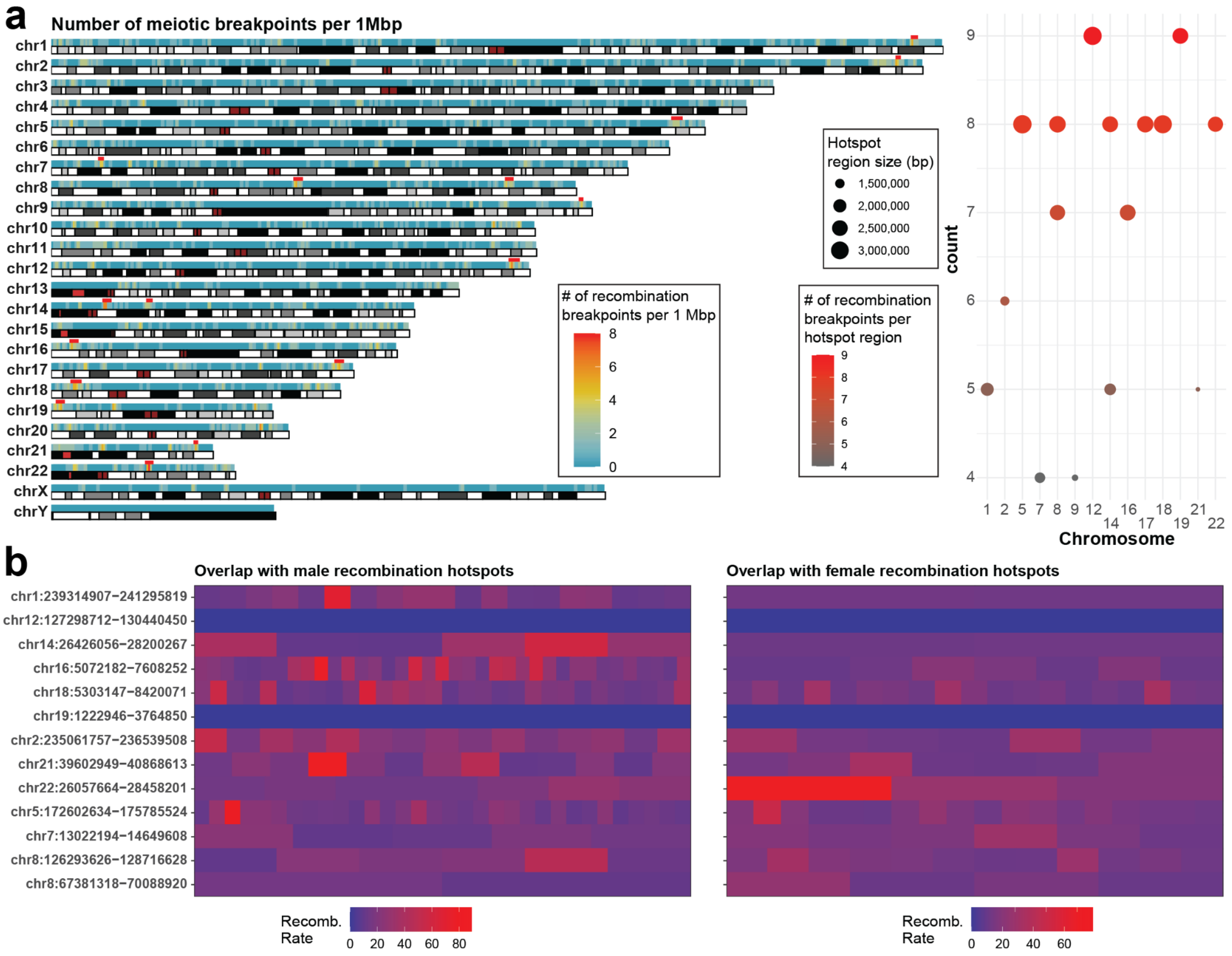

Figure S27: Recombination breakpoint hotspots (T2T-CHM13).

a) An ideogram showing the binned counts of all recombination breakpoints in G2-G4 (n=1,503) summarized in 1 Mbp long bins sliding by 500 kbp as a heatmap on top of each chromosomal ideogram. Recombination hotspots were predicted using primatR (https://github.com/daewoooo/primatR) function 'hotspotter' with the following parameters: gr = {GRanges object of all recombination breakpoints}, bw = 1000000, pval = 0.0005 (**Supplementary Table 8**). These are highlighted on top of each chromosomal heatmap as red rectangles (n=16). The far-right inset shows a summary of putative recombination hotspots per chromosome (x-axis) where the color of each point represents the number of meiotic breakpoints in a given region while the size of each point represents the size of the region. **Note:** Maternal and paternal meiotic breakpoints were not separated in this analysis but rather analyzed as a single set of recombination breakpoints. **b**) A comparison of the overlap of the 13 predicted autosomal recombination hotspots reported for this pedigree with male and female meiotic recombination hotspots from the deCODE project (Kong et al. 2010). Shades of red and blue rectangle depict hot and cold recombination clusters from the deCODE project, respectively.

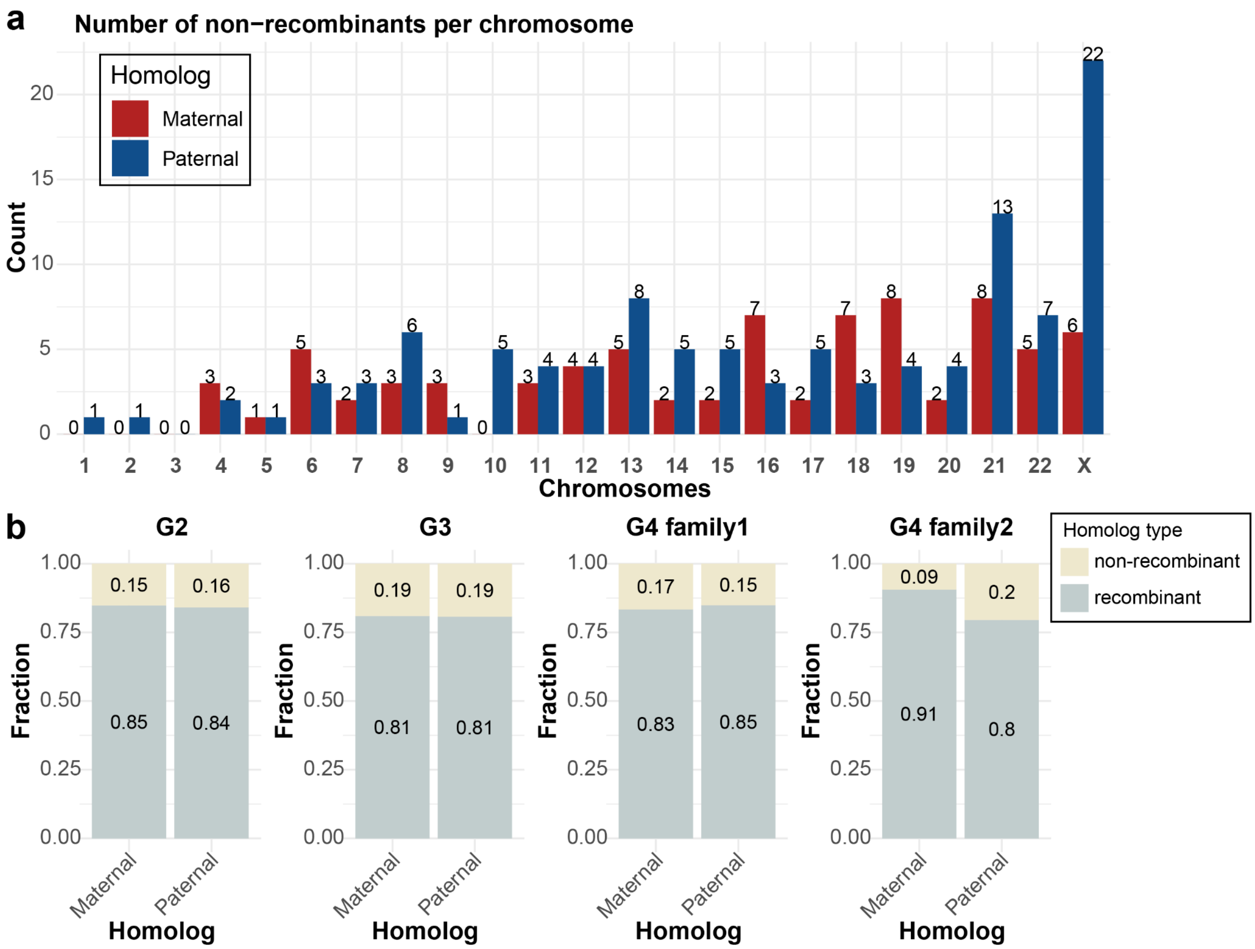

Figure S28: Summary of observed recombinant and nonrecombinant parental alleles.

a) Barplot showing the counts of nonrecombinant homologs per chromosome and across the complete recombination map of G2-G4. **b**) Fraction of recombinant and nonrecombinant homologs across G2-G4 individuals. Two G4 subfamilies are marked (family1 and family2).

Note: Male Chromosome X is not considered here.

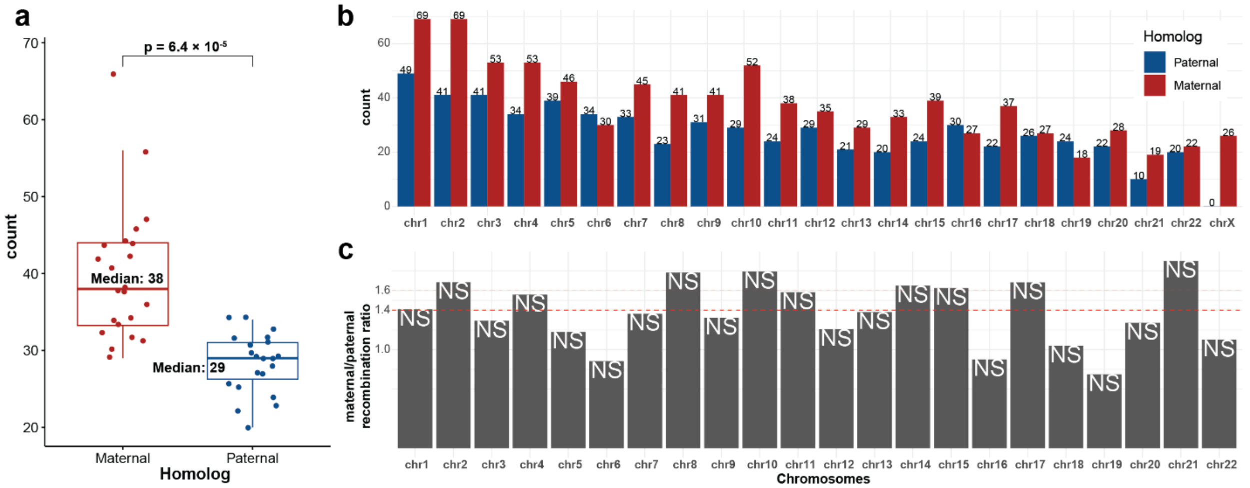

Figure S29: Maternal and paternal recombination counts and their ratios per chromosome.

a) Distribution of total maternal and paternal recombination breakpoints for each G2-G4 sample (n=22). The p-value (Wilcoxon signed-rank test) is shown on top. Boxes represent IQR, including median line; whiskers extend to 25% − 1.5 × IQR and 75% + 1.5 × IQR. **b**) A barplot showing a total number of maternal (red) and paternal (blue) recombination breakpoints across G2-G4 samples (n=22). **c**) A barplot showing the maternal versus paternal breakpoint ratios (based on count from top panel). Significance was evaluated using z-score calculation followed by p-value transformation expecting two-tailed distribution.

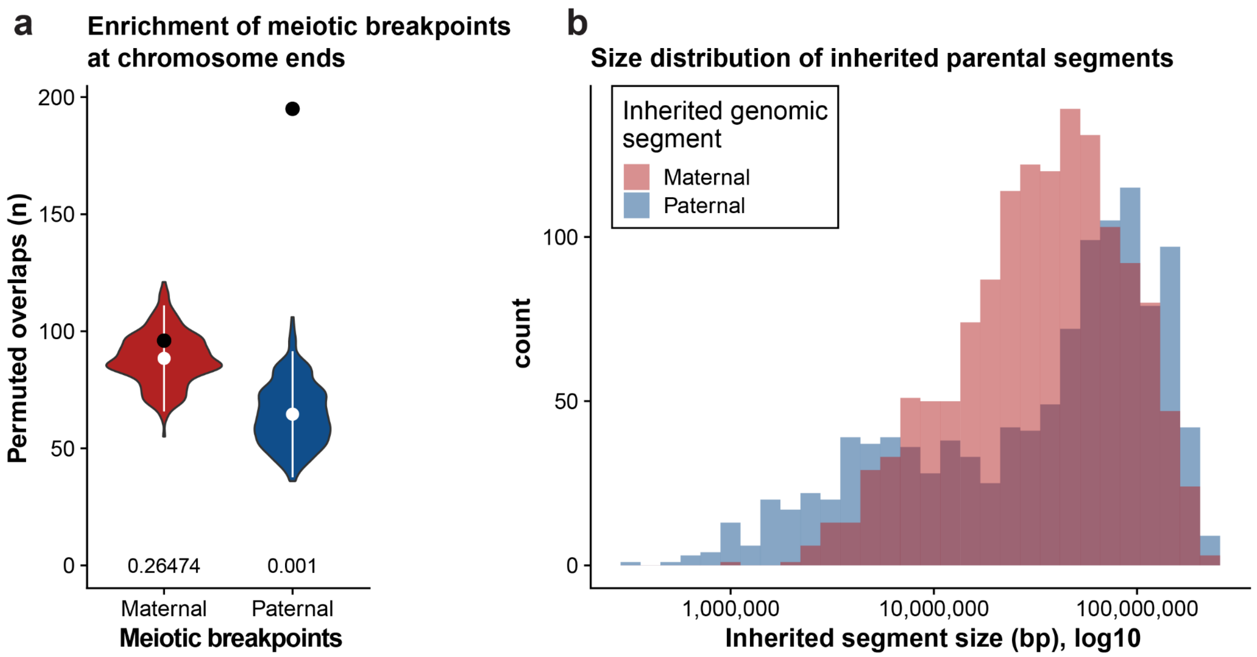

Figure S30: Biased distribution of recombination breakpoints (T2T-CHM13).

a) A violin plot showing the distribution of shuffled meiotic recombination counts (for all G2-G4 samples; n=22) at ends of each chromosome (last 5% size of each chromosome; **Methods**). The white points show the mean value of the distribution while the black point shows observed counts of the original breakpoint position at the defined chromosome ends. At the bottom of each violin plot there is a p-value (permutation test) associated with the differences between observed and rescheduled counts of recombination breakpoints at chromosome ends. **b**) A histogram showing the distribution of maternally (red) and paternally (blue) inherited DNA segments across all G2-G4 samples (n=22) (histogram bin size: 30).

Note: Segment sizes on x-axis are reported after log10 transformation.

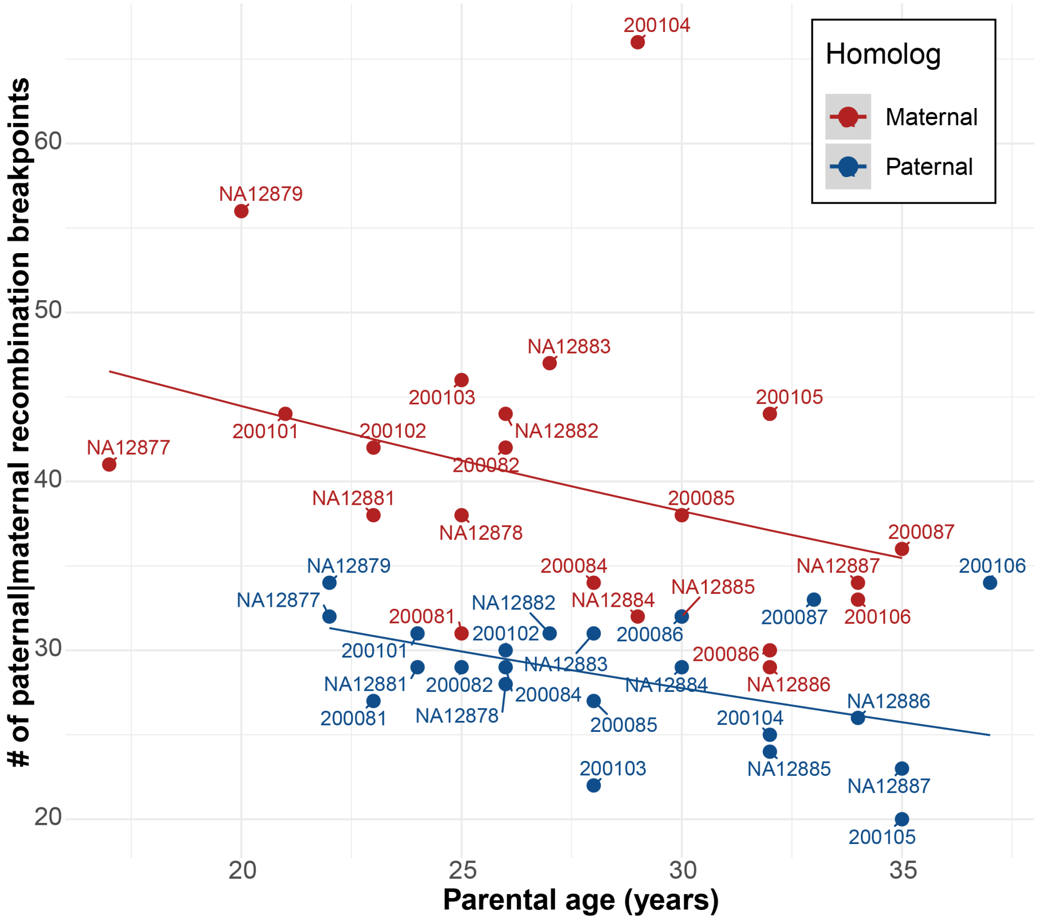

Figure S31: Recombination breakpoints and parental age.

Significant association between the number of recombination breaks (y-axis) and parental age (x-axis) shown separately for maternal (red) and paternal (blue) recombination breakpoints detected with respect to T2T-CHM13 for all G2-G4 individuals. Regression lines were fitted using Poisson generalized linear model (GLM) with a log link ($p=7.17\times10^{-3}$ and 1.22×10^{-9} for parental age and sex, respectively).

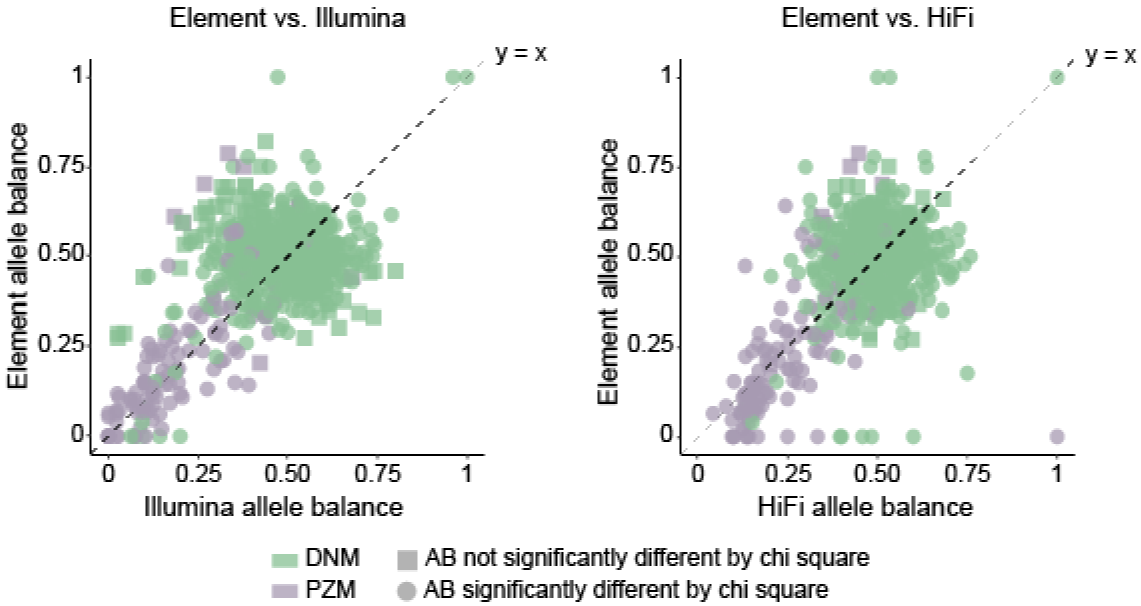

Figure S32: Evaluation of single-nucleotide DNMs using Element data.
The allele balance (AB) of each autosomal SNV call (n=626 DNMs; n=129 PZMs, including 10 false positive calls) in Illumina or HiFi plotted against Element. By chi-squared test, there is no significant difference in AB (p-value < 0.05) between Illumina and Element for 99.2% and 91.7% of DNMs and PZMs, respectively. Between HiFi and Element, there is no significant difference (p-value < 0.05) for 96.0% and 92.5% of DNMs and PZMs, respectively.

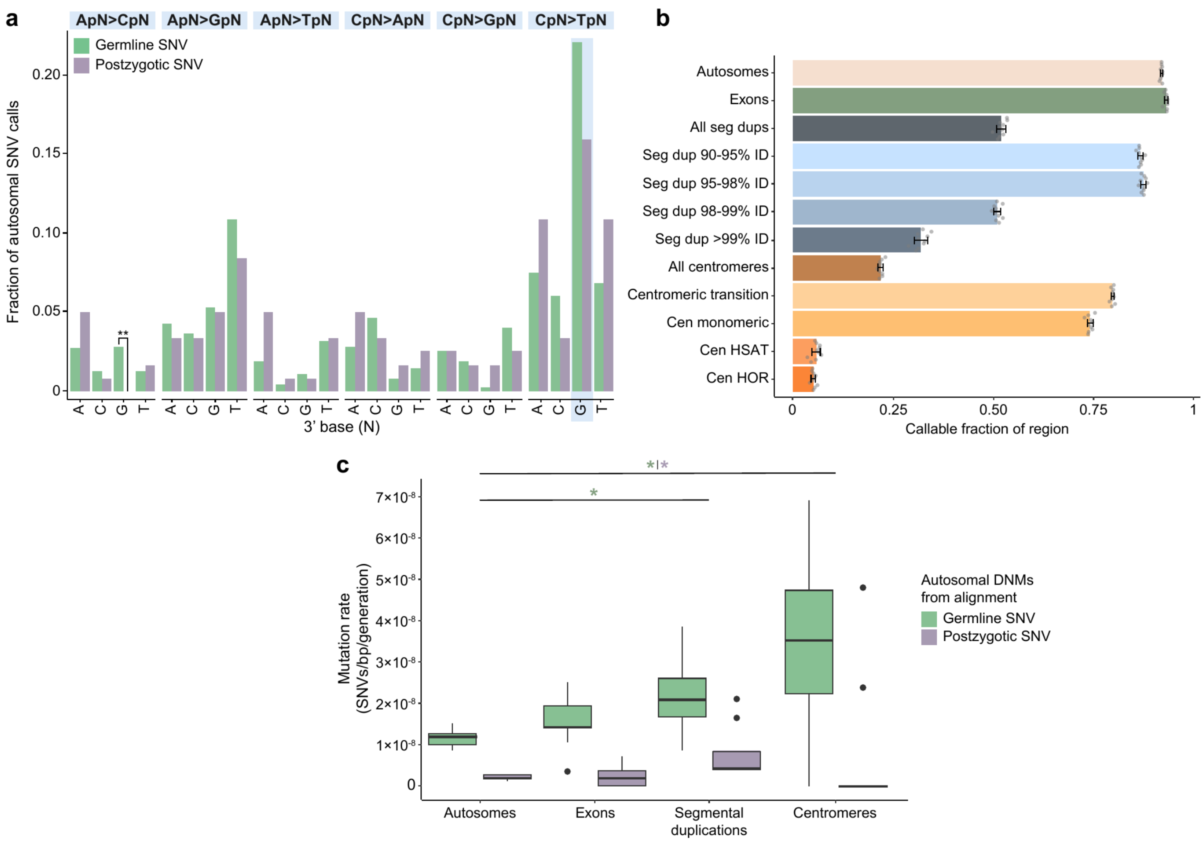

Figure S33: Germline and postzygotic mutation spectrum and rates.

a) Dinucleotide mutation spectrum of germline *de novo* SNVs (n=626) and postzygotic SNVs (n=110). No differences in single nucleotide substitution frequency rise to significance, but when accounting for dinucleotide context there is a significant depletion of postzygotic ApG>CpG substitutions (p<<10e-16) based on chi-squared tests corrected with Benjamini-Hochberg procedure. **b**) Mean amount of callable genome across samples (n=10), based on aligned HiFi reads. The black bars represent the first standard deviation. **c**) Per-sample mutation rates across different regions of the autosomes reveal significant enrichment in segmental duplications and centromeres. Boxes represent IQR including median line; whiskers extend to 25% − 1.5 × IQR and 75% + 1.5 × IQR, outliers are shown as dots. Significant difference from autosomal DNM or PZM rate determined by two-sided t-test, one asterisk (*) indicates $p < 0.05$. P-values for each comparison are: DNMs in exons: 0.10, PZMs in exons: 0.91, DNMs in segmental duplications: 0.0066, PZMs in segmental duplications: 0.049, DNMs in centromeres: 0.017, PZMs in centromeres: 0.34.

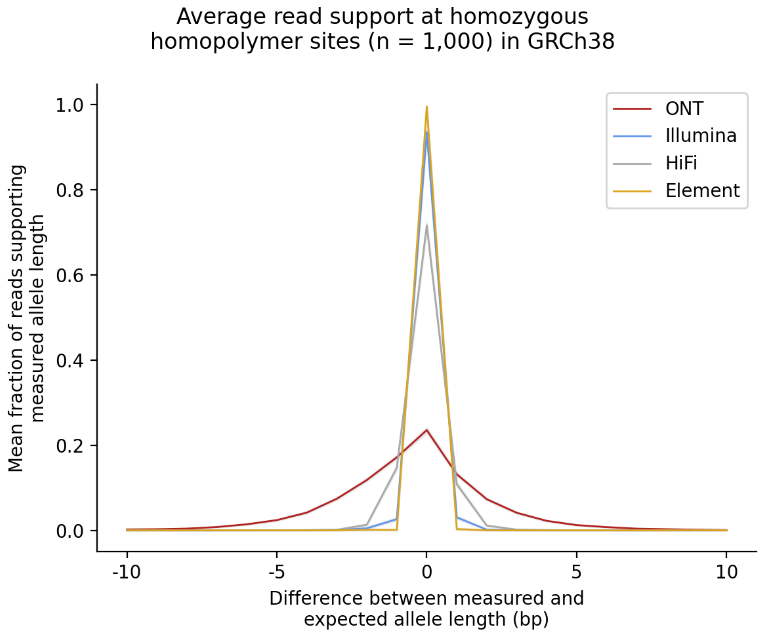

Figure S34: "Stutter" profiles at homozygous homopolymer loci using various sequencing technologies on sample G3-NA12879.

At each locus, we counted the net CIGAR operations in reads that overlapped the short tandem repeat (STR) locus; we considered the net total of CIGAR operations to be the "measured" allele length of the STR. We then compared the "measured" allele length to the "expected" allele length at each locus derived from TRGT output. An average of 99.5% (bootstrap 95% CI: 99.1 – 99.8%) of Element reads perfectly support the TRGT allele length followed by 93.5% (95% CI: 92.8 – 94.0) of Illumina reads; 71.6% (95% CI: 70.8 – 72.3) of HiFi reads; 23.5% (95% CI: 22.9 – 24.2) of ONT reads.

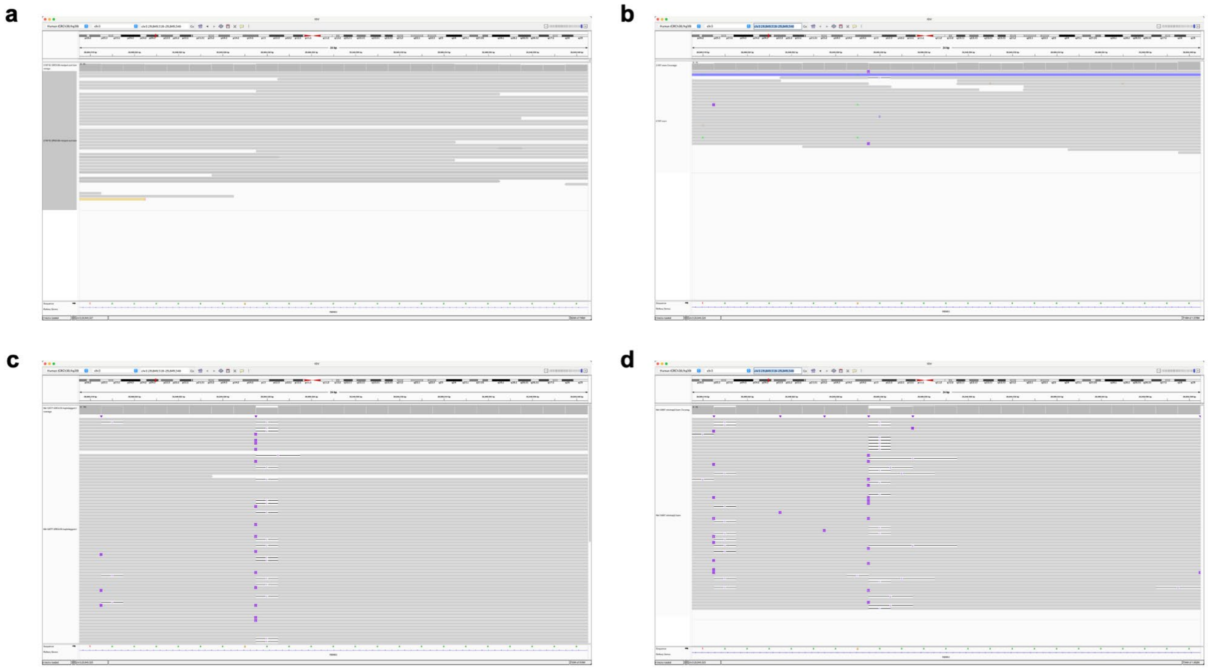

Figure S35: Read evidence from orthogonal technologies at a single homopolymer locus.

IGV screenshots of read evidence from **a**) Element AVITI, **b**) Illumina NovaSeq, **c**) PacBio HiFi, and **d**) ONT at a 22 bp $(A)_n$ homopolymer locus in GRCh38.

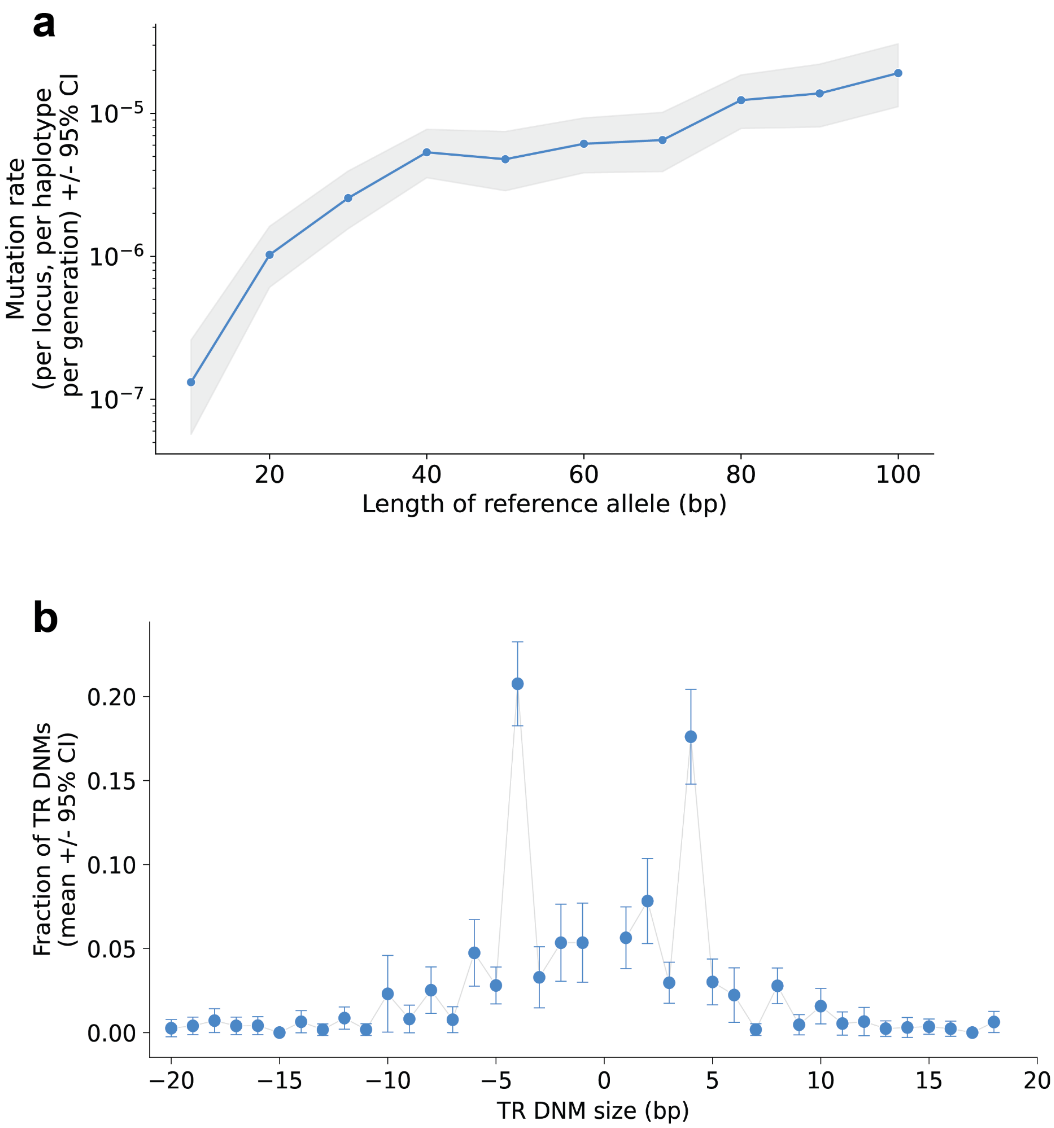

Figure S36: Patterns of TR DNMs.

a) Larger reference loci exhibit higher TR mutation rates. We binned all ~7.8 million TR loci by each locus' size (in bins of 10 bp) in the reference genome. We then binned all TR DNMs by reference allele size in the same fashion and computed TR DNM rates (expressed per bin, per haplotype, per generation) within each 10 bp bin by dividing the observed number of TR DNMs in a given bin by the total number of TR loci of the same bin size. **b**) Expansions and contractions are observed at similar frequencies. We plotted the average fraction of TR DNMs (±95% CI) of the specified size (in bp) across all G3 individuals. The plot is truncated at a maximum absolute DNM size of 25 bp.

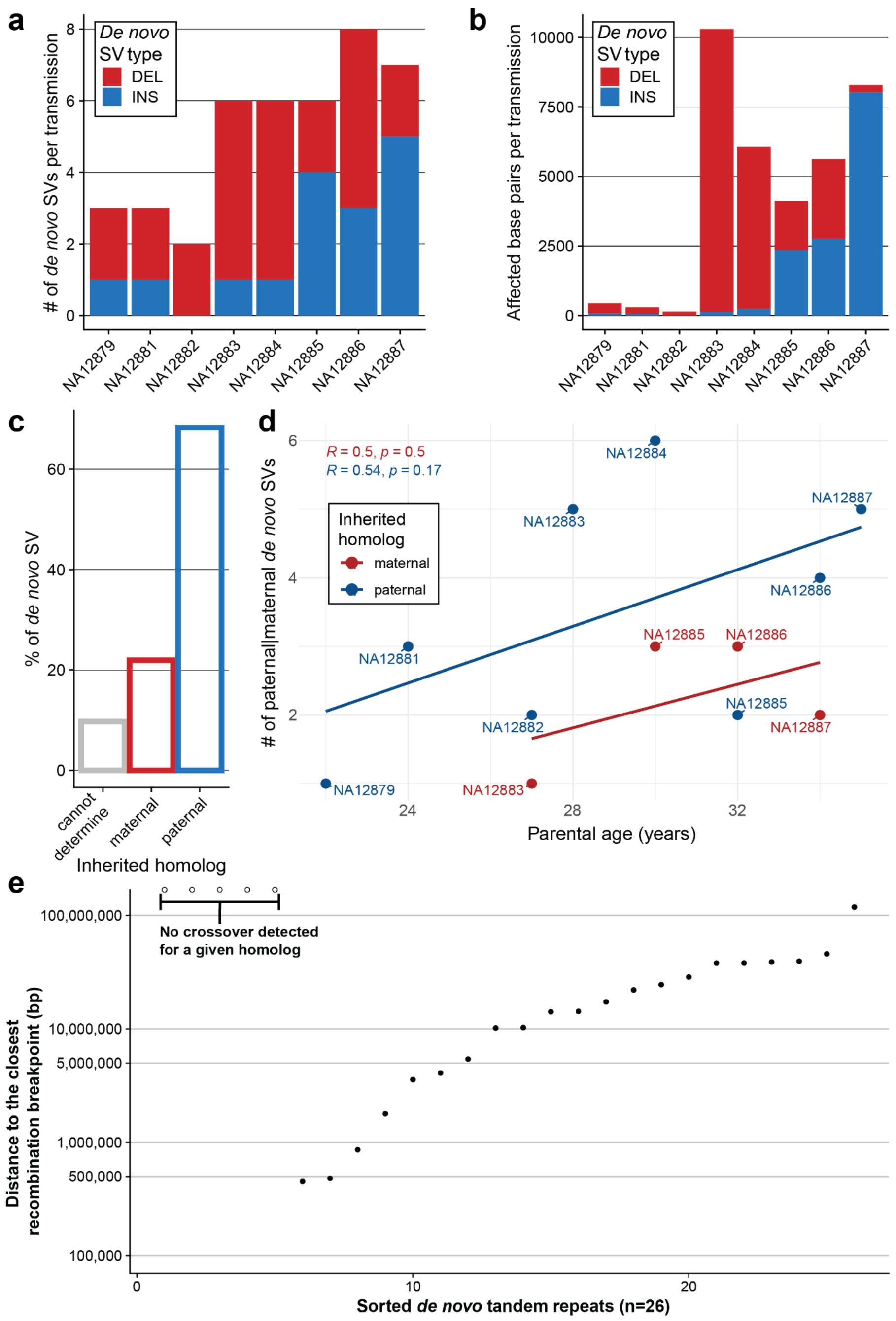

Figure S37: Summary of detected *de novo* SVs (n=41).

a) A barplot showing the total number of *de novo* SVs per G3 sample as stacked insertion (INS) and deletion (DEL) counts. **b**) A barplot showing the total number of bases affected by *de novo* SVs per G3 sample as stacked insertion (INS) and deletion (DEL) base-pair counts. **c**) A barplot showing the percentage of *de novo* SVs inherited from paternal (blue) or maternal (red) homologs. The gray bar shows *de novo* SVs where inheritance could not be reliably determined. **d**) Correlation between the number of *de novo* SVs (y-axis) and parental age (x-axis) shown separately for maternally (red) and paternally (blue) inherited homologs. **e**) Distance distribution of *de novo* TRs from detected cross-over in a given sample and chromosome. Empty points mark *de novo* TRs where no crossover was detected for a given chromosome in a given sample.

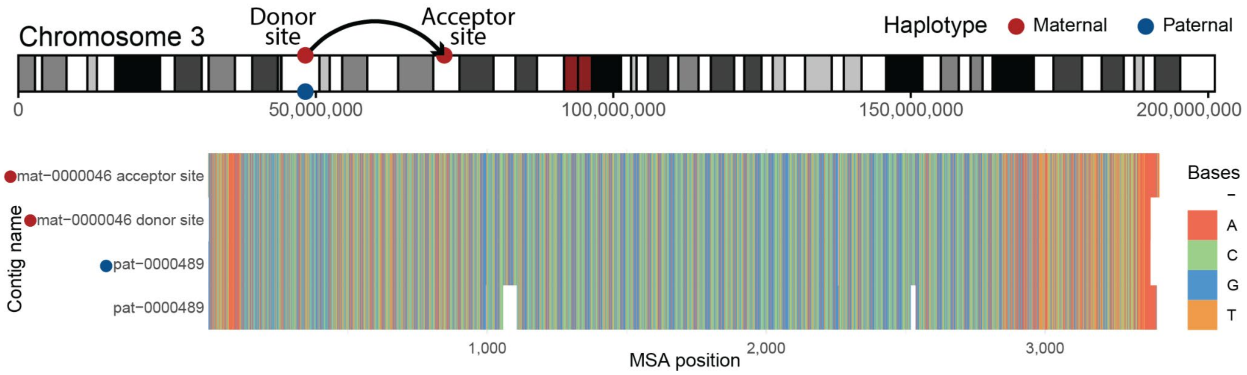

Figure S38: Predicting a donor site of *de novo* SVA insertion.

Top: Predicted donor and acceptor sites of the SVA element on the chr3 ideogram.

Bottom: An MSA between all SVA insertion positions in the *de novo* assembly of G3-NA12887. All positions of the SVA insertion were defined by mapping the inserted sequence against the *de novo* assembly of G3-NA12887 using minimap2 with the following parameters: `-x asm20 -c --eqx --secondary=yes`. Then the sequence of the SVA element was extracted from both maternal (red) and paternal (blue) assemblies and used to construct the MSA.

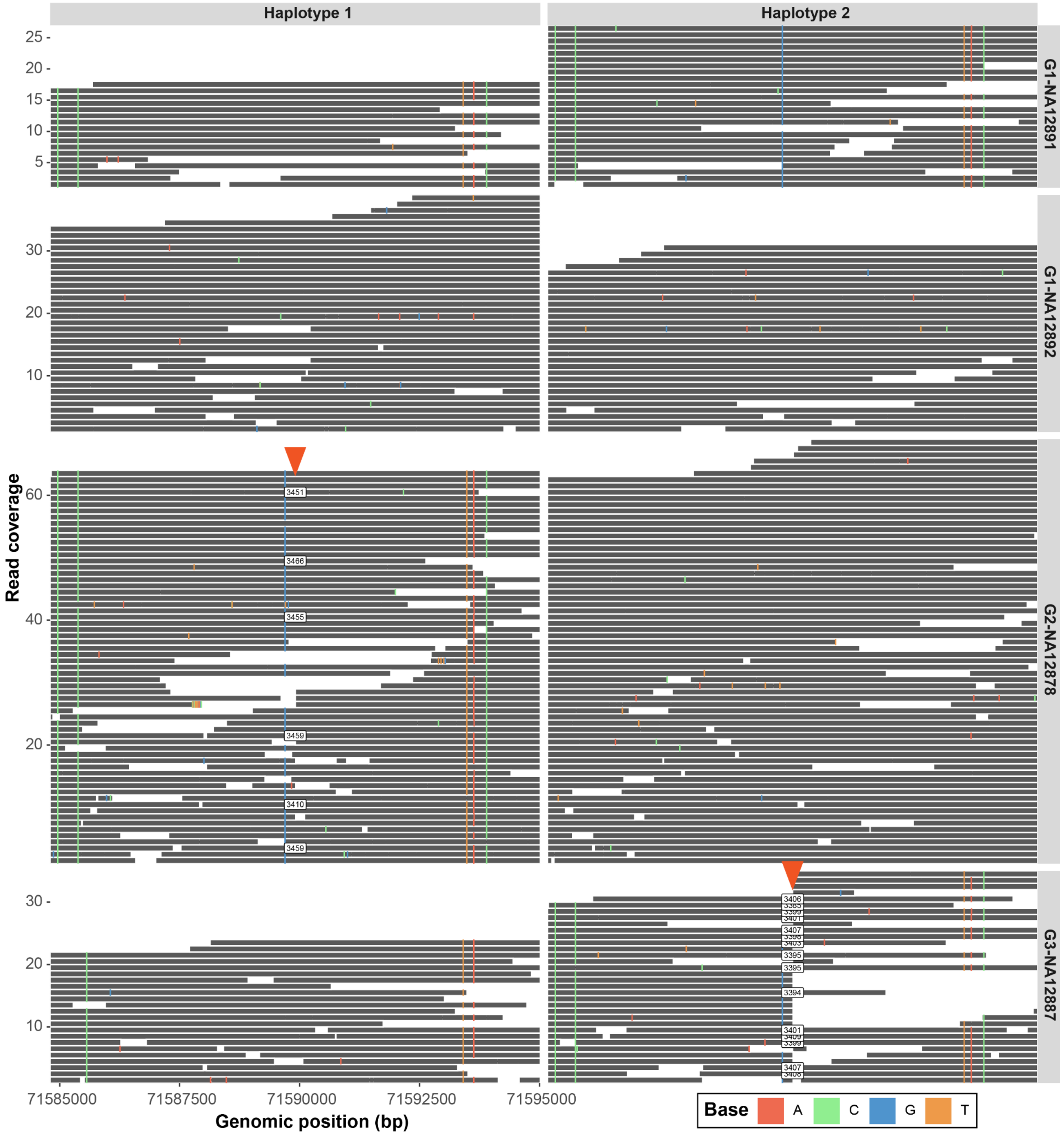

Figure S39: Predicting a donor site of *de novo* SVA insertion.

Visualization of HiFi reads aligned to the T2T-CHM13 reference for G1-G3 samples (G1 - NA12891, NA12892, G2-NA12878, and G3-NA12887) over the region (chr3:71584799-71595019) where the *de novo* SVA insertion was discovered. Each horizontal gray line represents a single HiFi read. Labels containing a number highlight the position and size of the SVA insertion (red arrowhead) in a given read. HiFi reads are stratified per sample (rows) and per haplotype (columns).

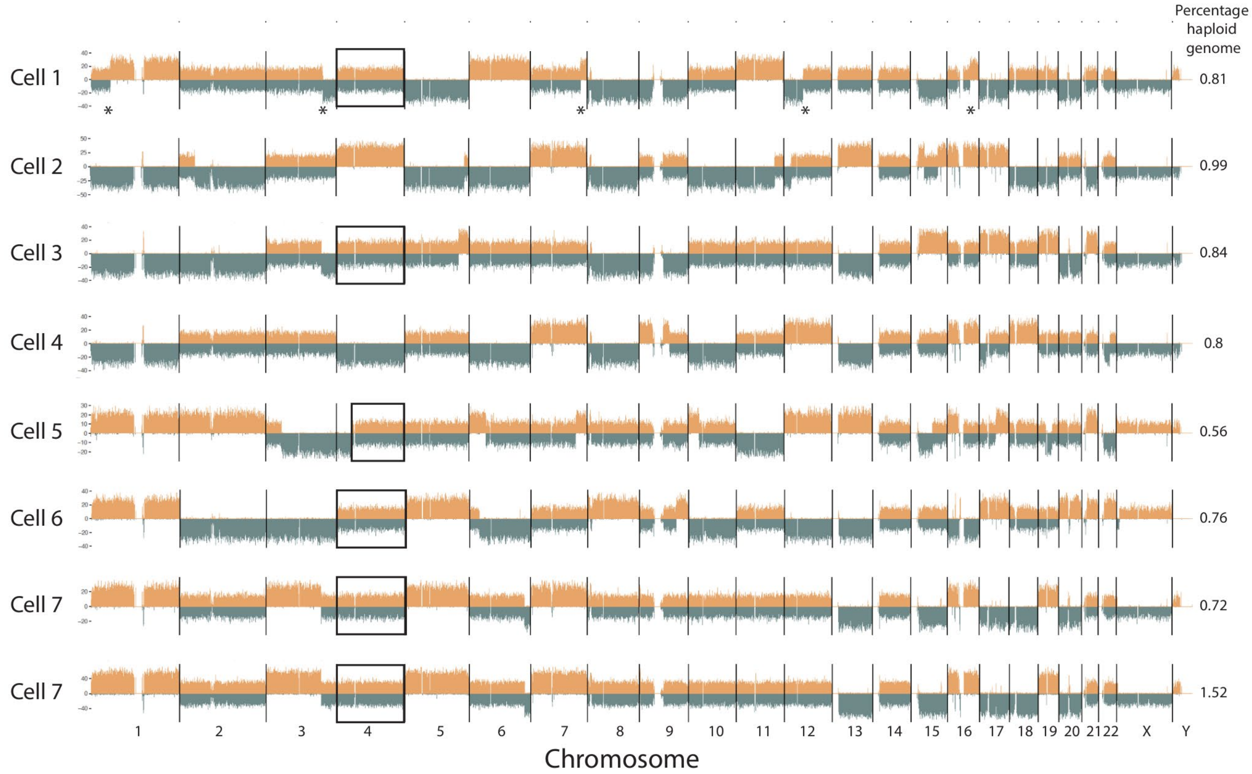

Figure S40: Example of Strand-seq libraries.

Sequence reads from seven individual cells from GM12877, plotted using BreakpointR (Porubsky et al. 2019), illustrate the power of Strand-seq libraries constructed with restriction enzymes for genome analysis. The number of reads in 0.2 Mbp bins mapping to the plus strand (Crick, top, orange) or the minus strand (Watson, bottom, teal) of the hg38 reference genome are plotted. Asterisks point to sister chromatid exchange events in cell 1. Boxes around Chromosome 4 point to cells with suitable data for generating chromosome-length haplotypes for this chromosome. Note that large inversions on Chromosomes 8 and 16 are easily recognized in a subset of the cells. For each individual, around 100 Strand-seq libraries were made, of which on average 80% passed ASHLEYS quality control criteria (Gros et al. 2021). Libraries of around 2000 cells were made in two library construction experiments, which included 14 individuals from G1-G3. One of the library pools was sequenced on the AVITI from Element Biosciences (San Diego, CA). Results obtained for the same cell on the NextSeq 550 and the AVITI are shown in the bottom two rows (results with AVITI at the bottom).

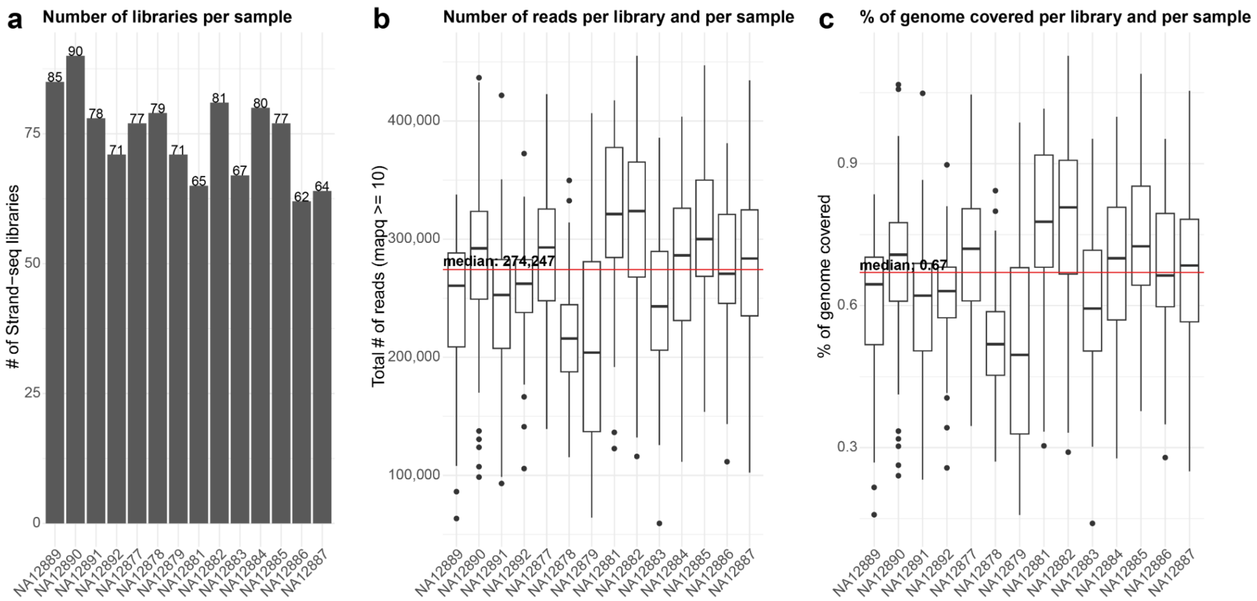

Figure S41: Strand-seq data summary for G1-G3 samples.

a) Number of selected libraries per sample. **b**) Distribution of mapped reads to the reference (T2T-CHM13) with mapping quality ≥10 per single-cell library and per sample. **c**) Distribution of percentage of reference genome (T2T-CHM13) covered by at least one read per single-cell library and per sample. Boxes in b and c represent IQR, including median line; whiskers extend to 25% − 1.5 × IQR and 75% + 1.5 × IQR, outliers are shown as dots.

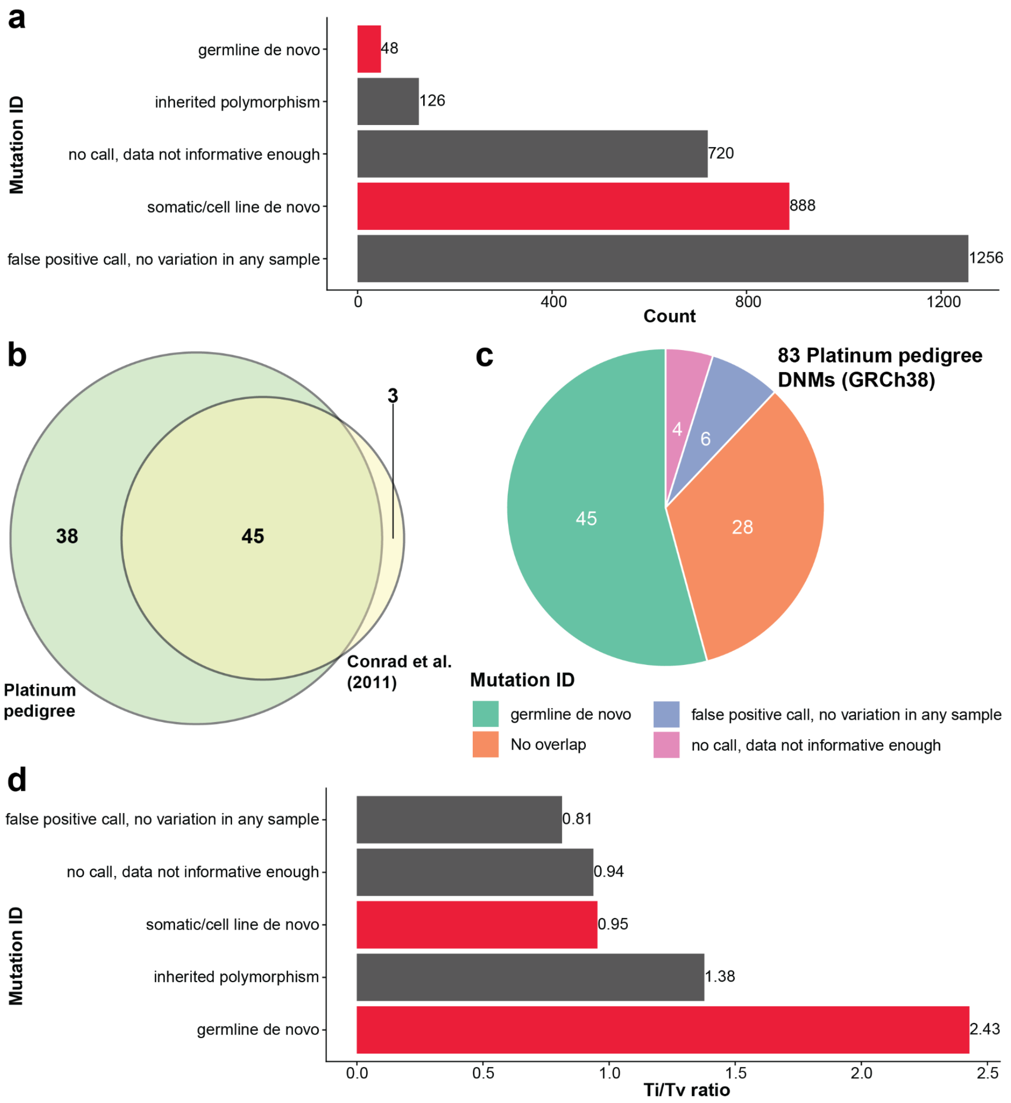

Figure S42: Evaluation of cell-line-specific artifacts.

a) Counts of various mutation classes reported by Conrad and colleagues (Conrad et al. 2011) stratified by mutation ID. A total of 3,038 sites were lifted over to GRCh38 coordinates. **b**) A Venn diagram showing overlap between the current set of single-nucleotide DNMs (Platinum pedigree; n=83) and Conrad and colleagues (n=48). **c**) Overlap of platinum pedigree DNMs with Conrad and colleagues DNMs colored by mutation ID. **d**) A barplot showing Ti/Tv ratios per defined mutation ID in Conrad and colleagues DNMs. Red bars highlight values reported for germline DNMs and somatic/cell line DNMs in comparison to other mutation classes reported by Conrad et al. (2011).

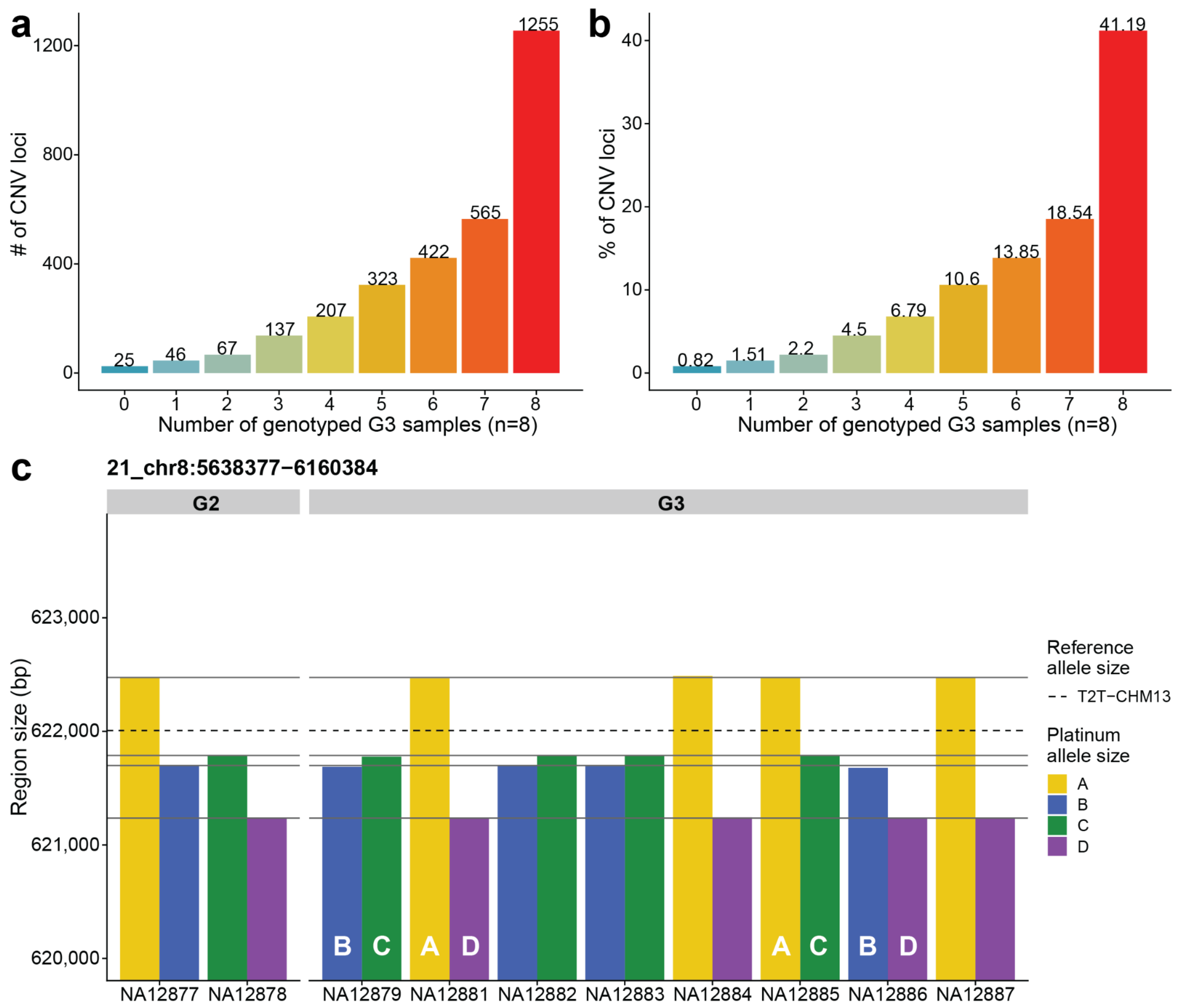

Figure S43: Assembly-based genotyping of known CNV regions.

a) A barplot showing the number of known CNV loci (y-axis) being genotyped in a given number of G3 samples (n=8; x-axis). **b**) A barplot showing the percentage of known CNV loci (y-axis) being genotyped in a given number of G3 samples (n=8; x-axis). **c**) An example of a CNV locus on Chromosome 8 (GRCh38 coordinates) genotyped in G3 samples (n=8) by measuring the size of a given region in phased genome assemblies. The size of each parental allele in G2 is marked by letters A-B and assigned a unique color.

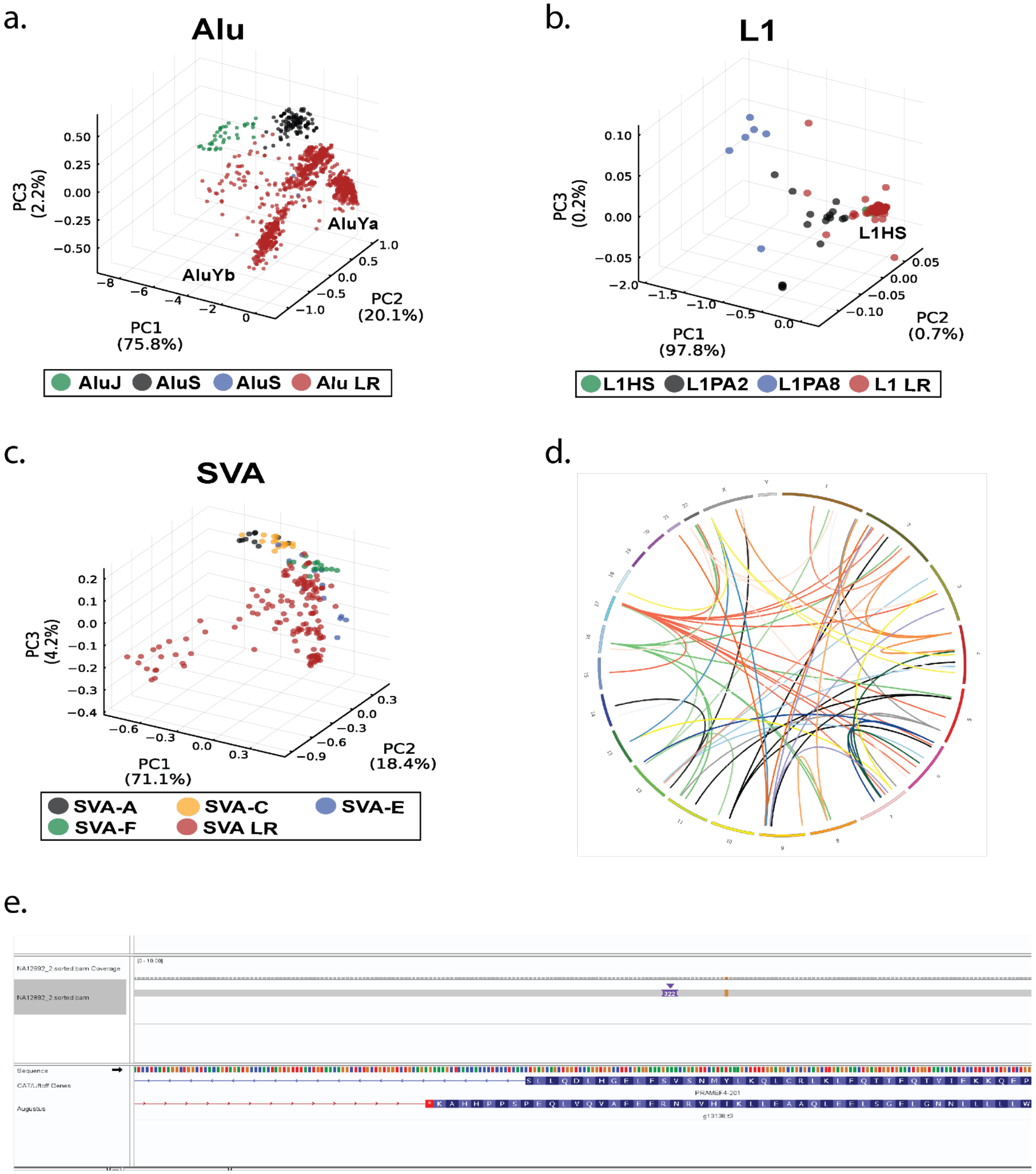

Figure S44: MEI analysis summary.

a-c) Principal component analyses of Alu, L1, and SVA insertions discovered in long-read (LR) sequencing data. For each MEI class, new (non-reference) MEI elements are shown in red. Selected MEIs from known subclasses are shown by additional colors. **a**) 2,158 new Alu insertions cluster predominantly with known AluY elements and show distinct clustering of AluYa and AluYb subfamilies, **b**) 112 new full-length L1 insertions cluster with mostly with L1HS sequences, and **c**) 149 new SVA elements show highest affinity to known SVA-E and SVA-F elements. Locations of selected subfamilies' clusters are shown on the plot for clarity. In general, the new MEIs are representative of MEI subfamilies known to be active in human lineages

(e.g., AluY, L1HS, SVA-E/F). Collectively, these MEIs represent ~1.7 Mbp of new non-reference MEI sequence (Alu: 605.4 kbp, L1: 687.1 kbp, and SVA 447.7 kbp) discovered using long-read sequencing technologies. **d**) Circos plot of the source elements responsible for two or more of the non-reference LINE-1 insertions identified in this study. The 20 loci represented in the plot are responsible for approximately 78% of the full-length non-reference LINE-1 insertions that had identifiable source elements. **e**) IGV image of a non-reference Alu insertion in an exon of *PRAMEF4* in an assembled genome.

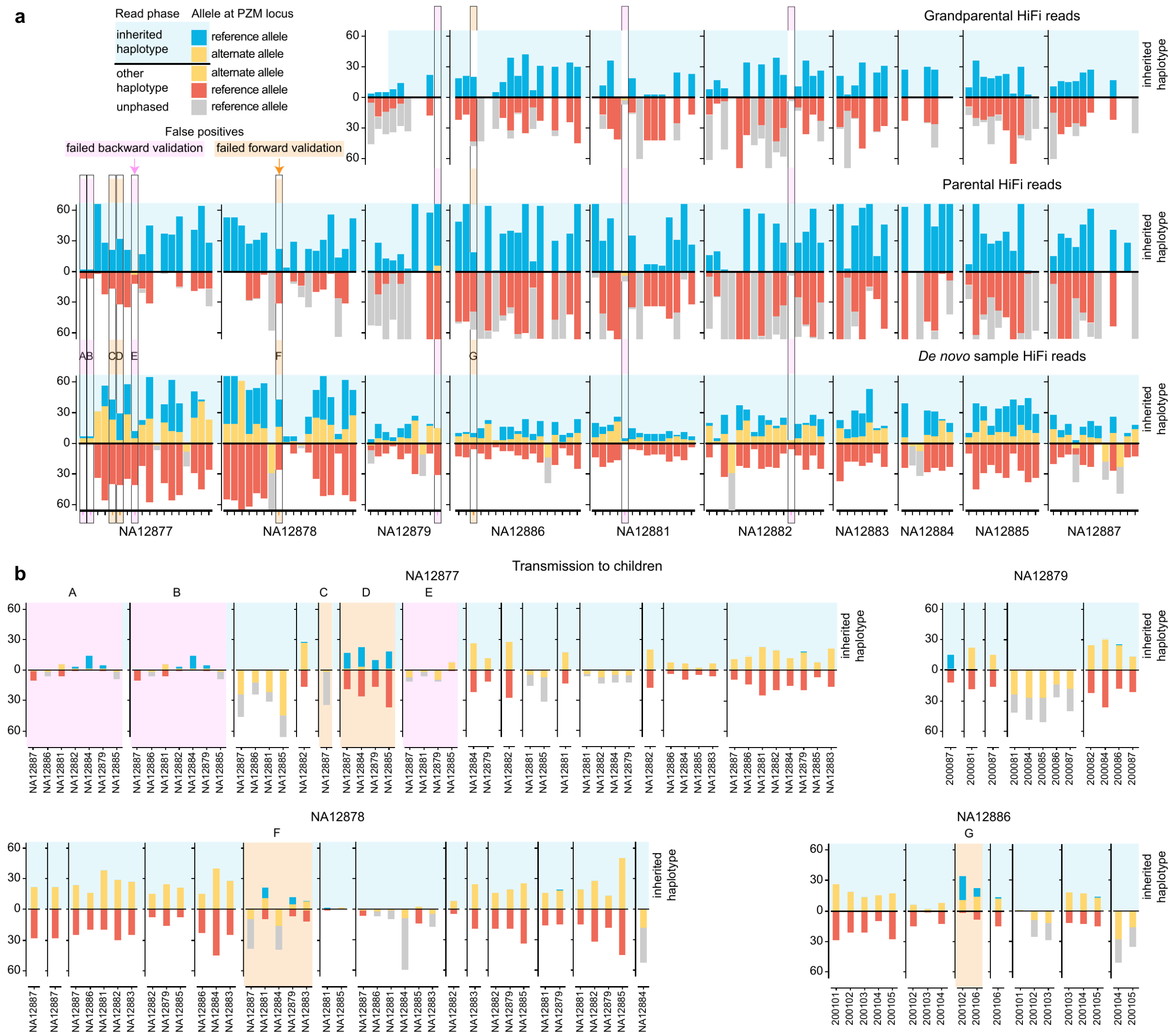

Figure S45: Phased haplotypes and allele counts.

a) Phased HiFi read counts for a *de novo* sample (bottom row), the parent from which they inherited the *de novo* haplotype (middle row), and the grandparent from which they inherited that haplotype (top row). Each column corresponds to a PZM (n=119), and missing read data indicates that a haplotype could not be uniquely assigned to a parent or grandparent. Reads assigned to the *de novo* haplotype are shown above the x-axis in blue, reads from the other haplotype are below the x-axis in red, and unphased reads are below the x-axis in grey. Reads with the alternate allele are shown in yellow. Variants in boxes are false positives - a pink highlight indicates that the variant failed backward validation by examining ancestors, and an orange highlight indicates that it failed forward validation by transmission. **b**) PZM transmissions to the next generation are shown. Transmissions are grouped by mutation (n=41), and one bar is shown for every transmission event. Lettered events correspond to the false positives shown in part (**a**).

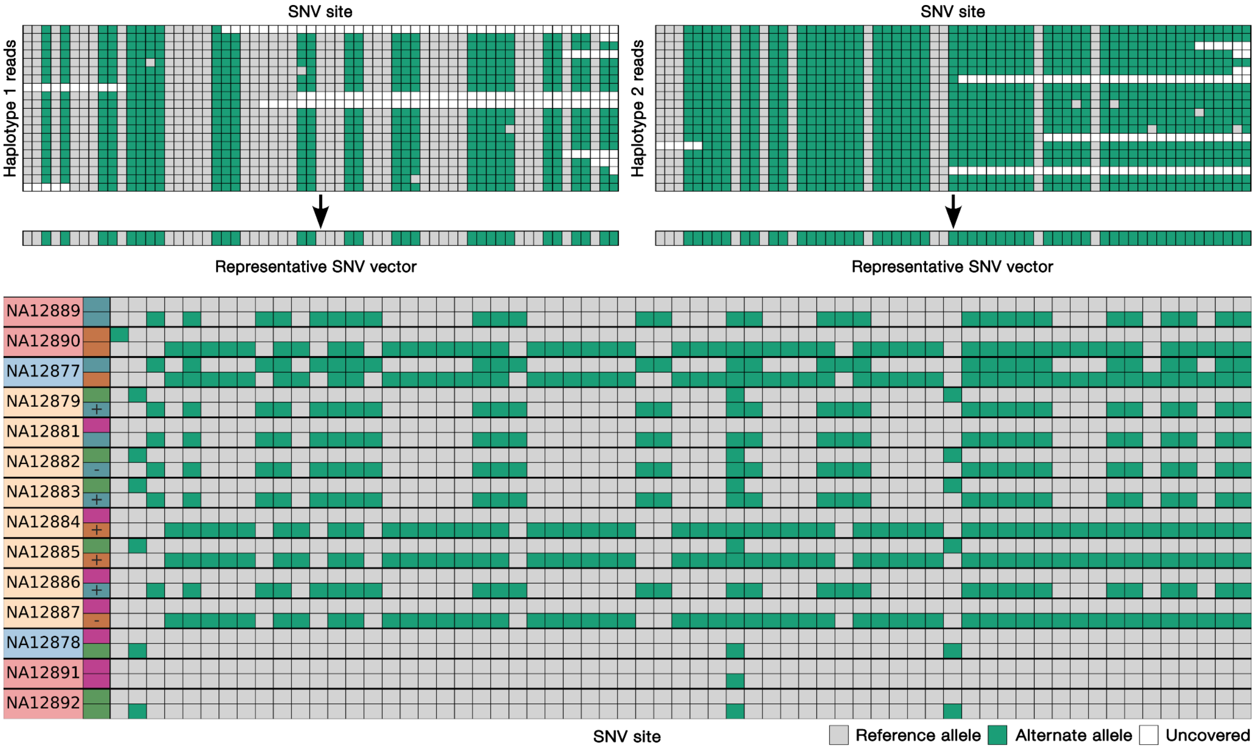

Figure S46: Transmission of flanking SNVs at recurrent TR locus.

a) Sites with informative SNVs within reads corresponding to each haplotype can be condensed into a representative SNV vector for that haplotype. **b**) The sample-haplotype stratified SNV matrix at chr8:2376919-2377075 (T2T-CHM13) across generations 1 to 3.

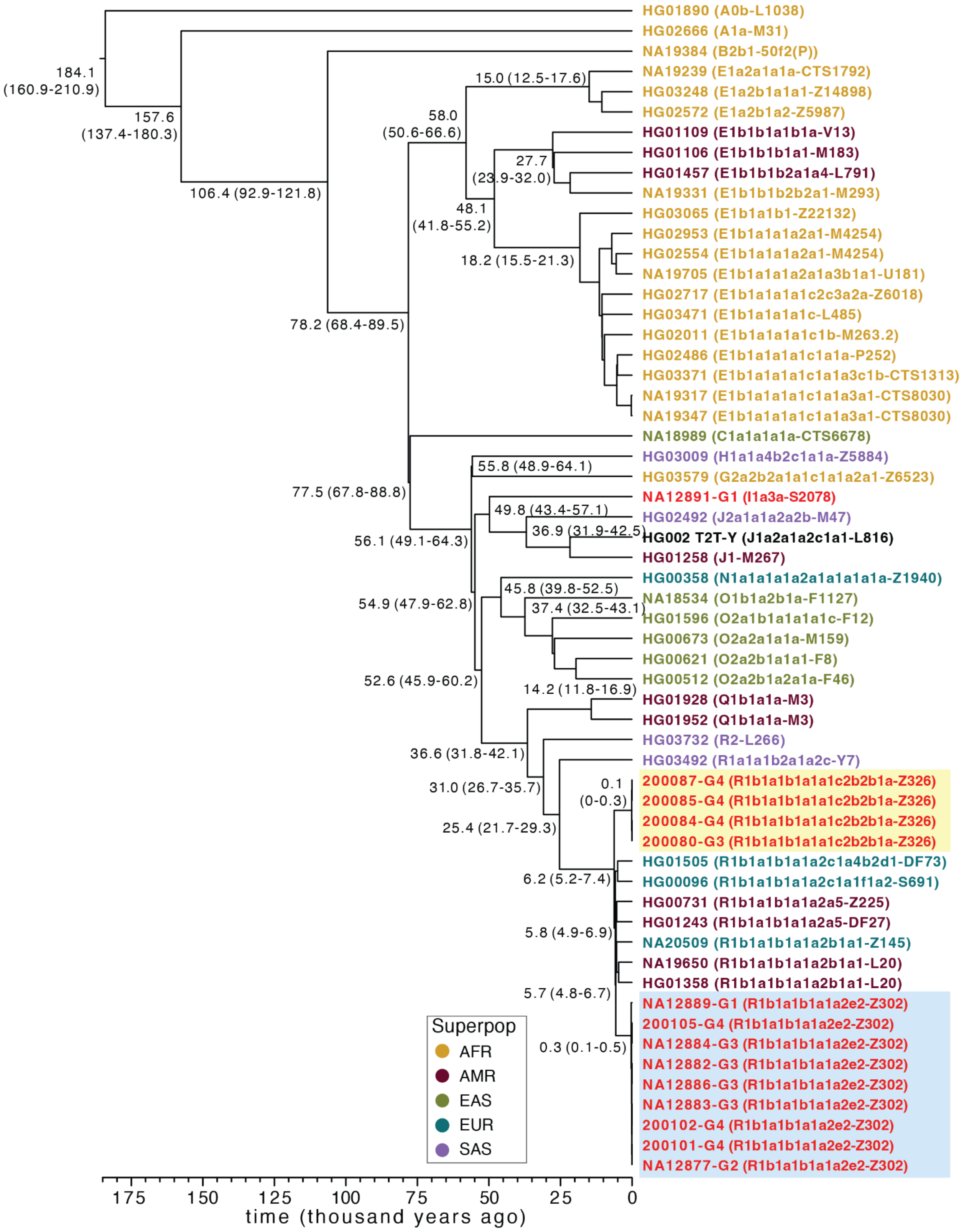
HG01890 (A0b-L1038)
HG02666 (A1a-M31)
NA19384 (B2b1-50f2(P))
NA19239 (E1a2a1a1a-CTS1792)
HG03248 (E1a2b1a1a1-Z14898)
HG02572 (E1a2b1a2-Z5987)
HG01109 (E1b1b1a1b1a-V13)
HG01106 (E1b1b1b1a1-M183)
HG01457 (E1b1b1b2a1a4-L791)
NA19331 (E1b1b1b2a1-M293)
HG03065 (E1b1a1b1-Z22132)
HG02953 (E1b1a1a1a2a1-M4254)
HG02554 (E1b1a1a1a2a1-M4254)
NA19705 (E1b1a1a1a2a1a3b1a1-U181)
HG02717 (E1b1a1a1a1c2c3a2a-Z6018)
HG03471 (E1b1a1a1a1c-L485)
HG02011 (E1b1a1a1a1c1b-M263.2)
HG02486 (E1b1a1a1a1c1a1a-P252)
HG03371 (E1b1a1a1a1c1a1a3c1b-CTS1313)
NA19317 (E1b1a1a1a1c1a1a3a1-CTS8030)
NA19347 (E1b1a1a1a1c1a1a3a1-CTS8030)
NA18989 (C1a1a1a1a-CTS6678)
HG03009 (H1a1a4b2c1a1a-Z5884)
HG03579 (G2a2b2a1a1c1a1a2a1-Z6523)
NA12891-G1 (I1a3a-S2078)
HG02492 (J2a1a1a2a2b-M47)
HG002 T2T-Y (J1a2a1a2c1a1-L816)
HG01258 (J1-M267)
HG00358 (N1a1a1a1a2a1a1a1a1a-Z1940)
NA18534 (O1b1a2b1a-F1127)
HG01596 (O2a1b1a1a1a1c-F12)
HG00673 (O2a2a1a1a-M159)
HG00621 (O2a2b1a1a1-F8)
HG00512 (O2a2b1a2a1a-F46)
HG01928 (Q1b1a1a-M3)
HG01952 (Q1b1a1a-M3)
HG03732 (R2-L266)
HG03492 (R1a1a1b2a1a2c-Y7)
200087-G4 (R1b1a1b1a1a1c2b2b1a-Z326)
200085-G4 (R1b1a1b1a1a1c2b2b1a-Z326)
200084-G4 (R1b1a1b1a1a1c2b2b1a-Z326)
200080-G3 (R1b1a1b1a1a1c2b2b1a-Z326)
HG01505 (R1b1a1b1a1a2c1a4b2d1-DF73)
HG00096 (R1b1a1b1a1a2c1a1f1a2-S691)
HG00731 (R1b1a1b1a1a2a5-Z225)
HG01243 (R1b1a1b1a1a2a5-DF27)
NA20509 (R1b1a1b1a1a2b1a1-Z145)
NA19650 (R1b1a1b1a1a2b1a1-L20)
HG01358 (R1b1a1b1a1a2b1a1-L20)
NA12889-G1 (R1b1a1b1a1a2e2-Z302)
200105-G4 (R1b1a1b1a1a2e2-Z302)
NA12884-G3 (R1b1a1b1a1a2e2-Z302)
NA12882-G3 (R1b1a1b1a1a2e2-Z302)
NA12886-G3 (R1b1a1b1a1a2e2-Z302)
NA12883-G3 (R1b1a1b1a1a2e2-Z302)
200102-G4 (R1b1a1b1a1a2e2-Z302)
200101-G4 (R1b1a1b1a1a2e2-Z302)
NA12877-G2 (R1b1a1b1a1a2e2-Z302)
184.1 (160.9-210.9)
157.6 (137.4-180.3)
106.4 (92.9-121.8)
78.2 (68.4-89.5)
58.0 (50.6-66.6)
15.0 (12.5-17.6)
27.7 (23.9-32.0)
48.1 (41.8-55.2)
18.2 (15.5-21.3)
77.5 (67.8-88.8)
55.8 (48.9-64.1)
56.1 (49.1-64.3)
49.8 (43.4-57.1)
36.9 (31.9-42.5)
54.9 (47.9-62.8)
45.8 (39.8-52.5)
37.4 (32.5-43.1)
52.6 (45.9-60.2)
14.2 (11.8-16.9)
36.6 (31.8-42.1)
31.0 (26.7-35.7)
25.4 (21.7-29.3)
0.1 (0-0.3)
6.2 (5.2-7.4)
5.8 (4.9-6.9)
5.7 (4.8-6.7)
0.3 (0.1-0.5)
Superpop
AFR
AMR
EAS
EUR
SAS
175
150
125
100
75
50
25
0
time (thousand years ago)

Figure S47: Phylogenetic relationships of long-read Y assemblies and pedigree Y chromosomes.

Y chromosomes from 14 pedigree males are combined with 44 individuals for which long-read-based Y assemblies have previously been published (Hallast et al. 2023). Split times as estimated according to the BEAST analysis are shown for major splits with 95% highest posterior density (HPD) intervals in brackets. Red text - indicates pedigree males, other colors indicate the 1000 Genomes Project continental groups. Yellow background shows the Y chromosomes of G3 spouse (200080) and his three male offspring. Blue background indicates the nine males with R1b1a-Z302 Y chromosomes analyzed in detail here (**Fig. 5a**).

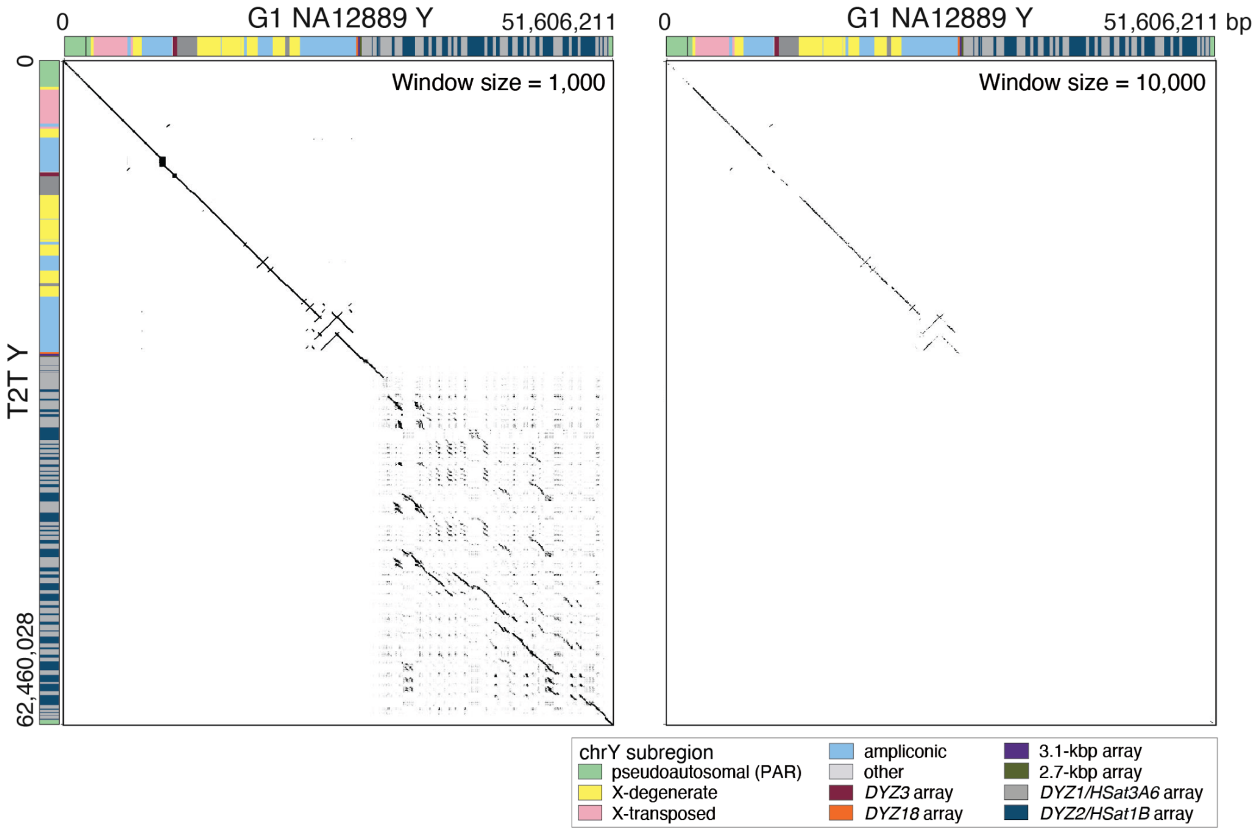

Figure S48: Comparison of G1-NA12889 and T2T-CHM13 Y chromosome sequences.

Dot plots of sequence similarity between the G1-NA12889 Y assembly and the T2T-CHM13 Y chromosome sequence (Rhie et al. 2023). The T2T Y (J1a-L816 Y haplogroup) last shared a common ancestor with the pedigree R1b1a-Z302 Y chromosome approximately 54,900 years ago (95% HPD interval = 45,900–60,200 years ago; **Supplementary Fig. 47** and differs extensively especially in the repetitive regions of the Y chromosome. Y-chromosomal sequence classes are shown as colored bars, with the assembly break in the PAR1 of NA12889 indicated by a black line. The dot plot is generated with word size of 1,000 bp shown on the left and word size of 10,000 bp on the right.

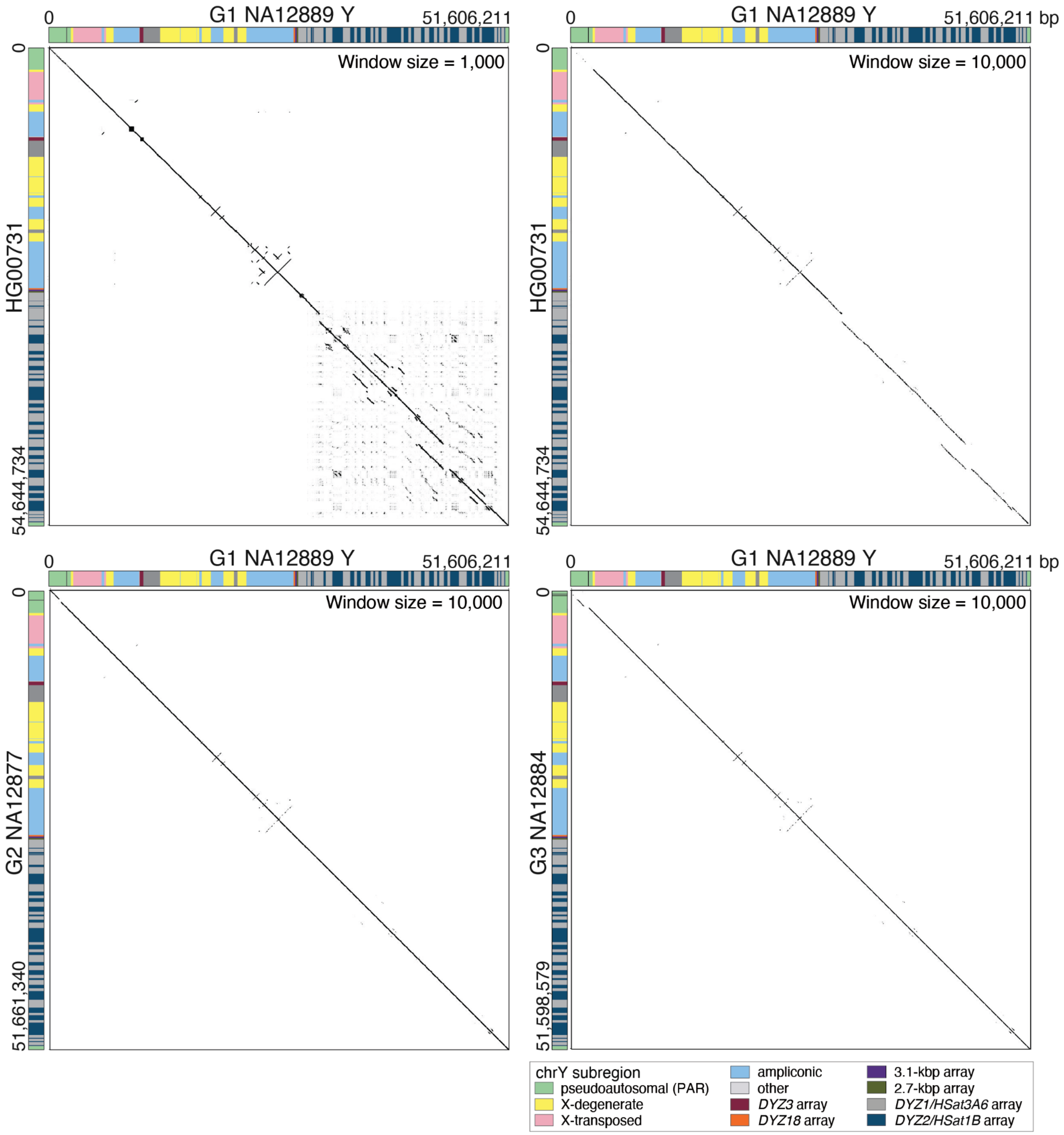

Figure S49: Comparison of chrY assemblies.

Dot plots of sequence similarity between the G1 NA12889 Y assembly, evolutionarily closely related Y chromosome from HG00731 (top), G2-NA12877 (bottom left), and G3-NA12884 (bottom right) assemblies. The HG00731 Y (R1b1a-Z225 Y haplogroup) last shared a common ancestor with the pedigree R1b1a-Z302 Y chromosome approximately 5,700 years ago, 95% HPD interval = 4,800–6,700 years ago, **Supplementary Fig. 47**. Window sizes of 1,000 and 10,000 bp are shown for HG00731, and 10,000 bp for G2 and G3 males, indicating high levels of sequence similarity between the Y assemblies. Y-chromosomal sequence classes are shown as colored bars, with assembly breaks in the PAR1 indicated by black lines.

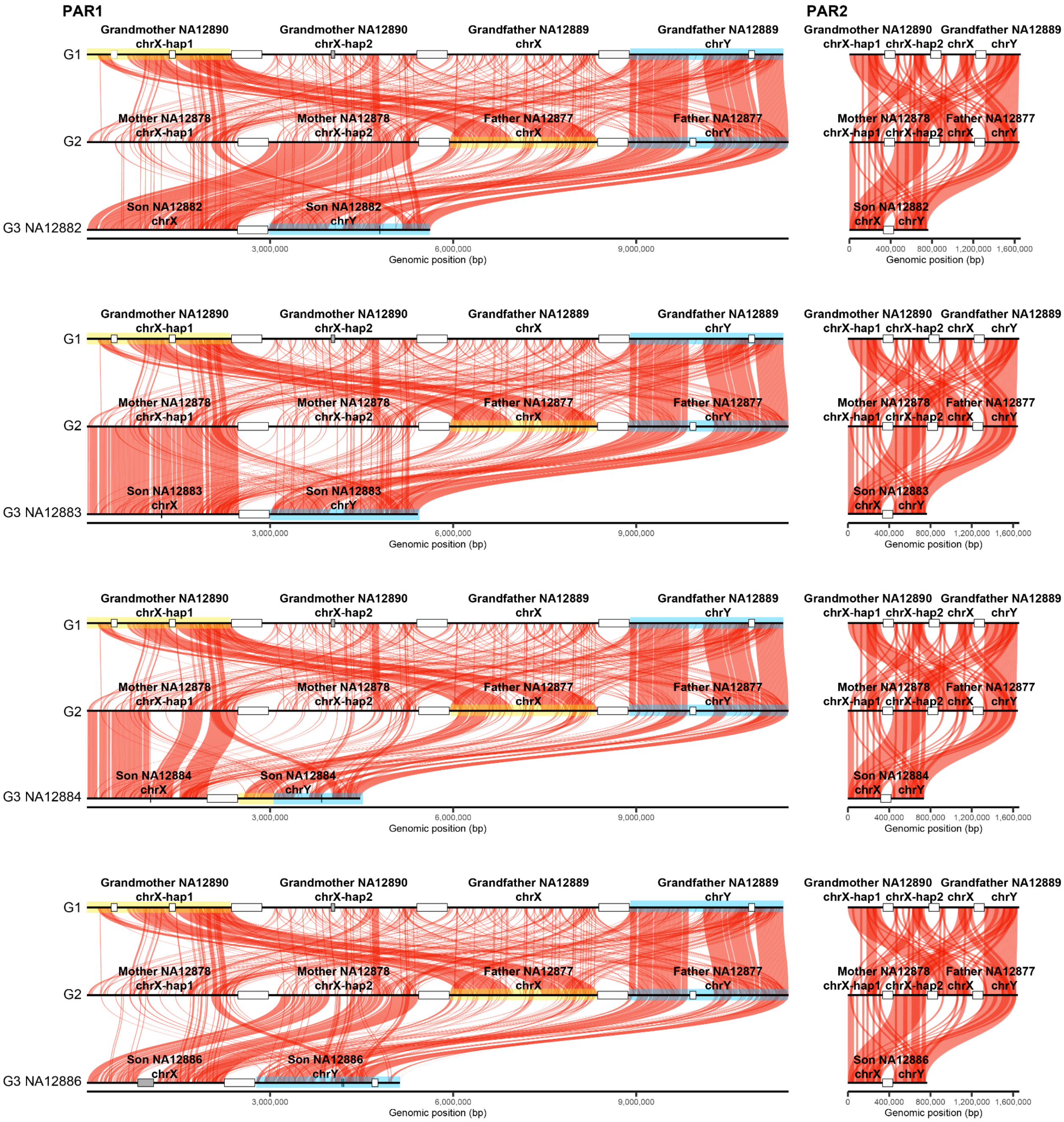

Figure S50: Assembled chrX and chrY pseudoautosomal regions (PARs) across three generations.

Binned alignments (10 kbp bin size) of ≥99.9% sequence identity are shown for PAR1 (left) and PAR2 (right) across generations. For G1 and G2, maternal sequences for chrX haplotypes (hap) 1 and 2, and paternal chrX and chrY PAR sequences are shown. For each of the G3 males, chrX and chrY PAR sequences are shown. Large white rectangles (equal in size to 500 kbp) separate the haplotypes, while 100 kbp-sized rectangles indicate where joints were made if several contigs represented the region for a specific individual. Gray rectangles indicate blocks of N's in the contigs. Yellow and blue rectangles indicate chrX and chrY PAR1 haplotypes that show evidence of recombination in G3 male NA12884. No recombination events were identified in PAR2. The following contigs were included for PAR1 (in the order as visualized from left to right): G1 NA12890 chrX haplotype 1 - haplotype1−0000079, haplotype1-0000078

and haplotype1-0000010, chrX haplotype 2 - haplotype2-0000100; G1 NA12889 chrX - haplotype1-0000018 and chrY - haplotype2-0000082 and haplotype2-0000081; G2 NA12878 chrX haplotype 1 - mat-0000002, chrX haplotype 2 - pat−0000758; G2 NA12877 chrX - mat−0000005 and chrY - pat−0000406 and pat−0000383; G3 NA12882 chrX - mat−0000046 and chrY - pat−0000587; G3 NA12883 chrX - mat−0000038 and chrY - pat−0000576; G3 NA12884 chrX - mat−0000008 and chrY - pat−0000224; G3 NA12886 chrX - mat−0000010 and chrY - pat−0000781 and pat−0001035; and for PAR2: G1 NA12890 chrX haplotype 1 - haplotype1-0000010, chrX haplotype 2 - haplotype2-0000096, G1 NA12889 chrX - haplotype1-0000018, chrY - haplotype2-0000081; G2 NA12878 chrX haplotype 1 - mat-0000002, chrX haplotype 2 - pat-0000758; G2 NA12877 chrX - mat-0000005, chrY - pat-0000383; G3 NA12882 chrX - mat-0000046 and chrY - pat-0000570; G3 NA12883 chrX - mat-0000045 and chrY - pat-0000567; G3 NA12884 chrX - mat-0000008 and chrY - pat-0000224; G3 NA12886 chrX - mat-0000010 and chrY - pat-0000749.

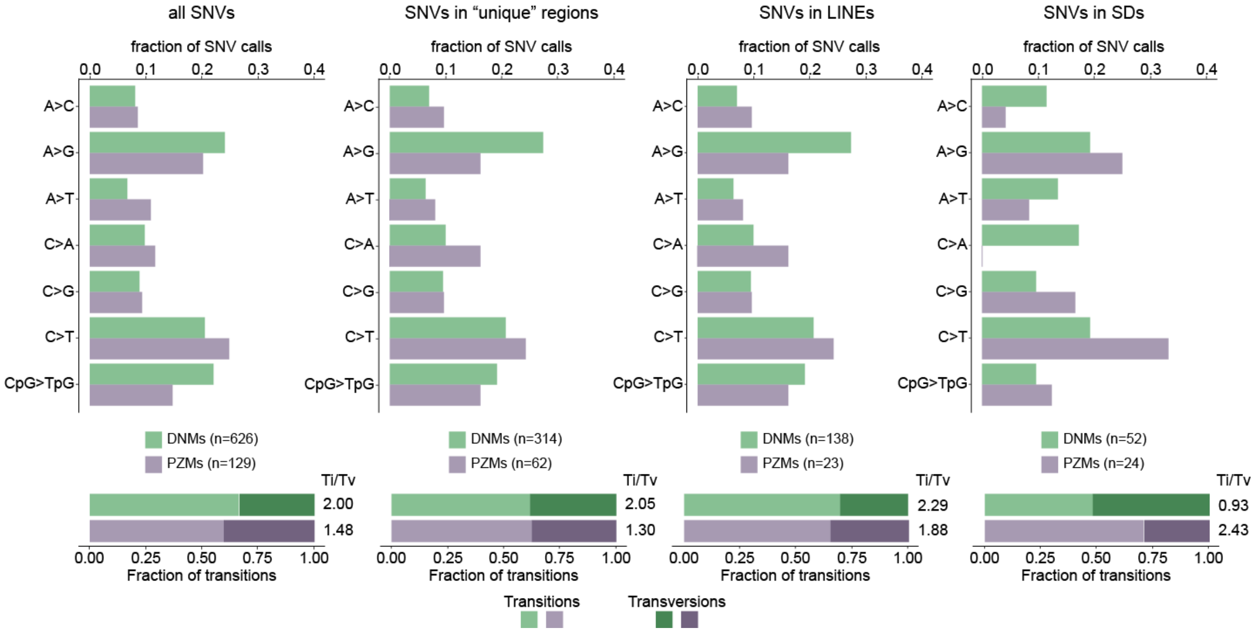

Figure S51: Mutation spectra in unique and repeated regions.
We examined all autosomal DNMs and PZMs, as well as subsets in LINEs, segmental duplications (SDs) and unique regions (autosomes without centromeres, acrocentric p-arms, SDs, transposable elements, or simple repeats) to compare the single-nucleotide substitution spectra. There were no significant enrichments of any mutational class in SDs or LINEs compared to unique sequence, but the transition/transversion (Ti/Tv) ratio is significantly lower in SDs compared to unique sequence (chi-squared test, p=0.012).

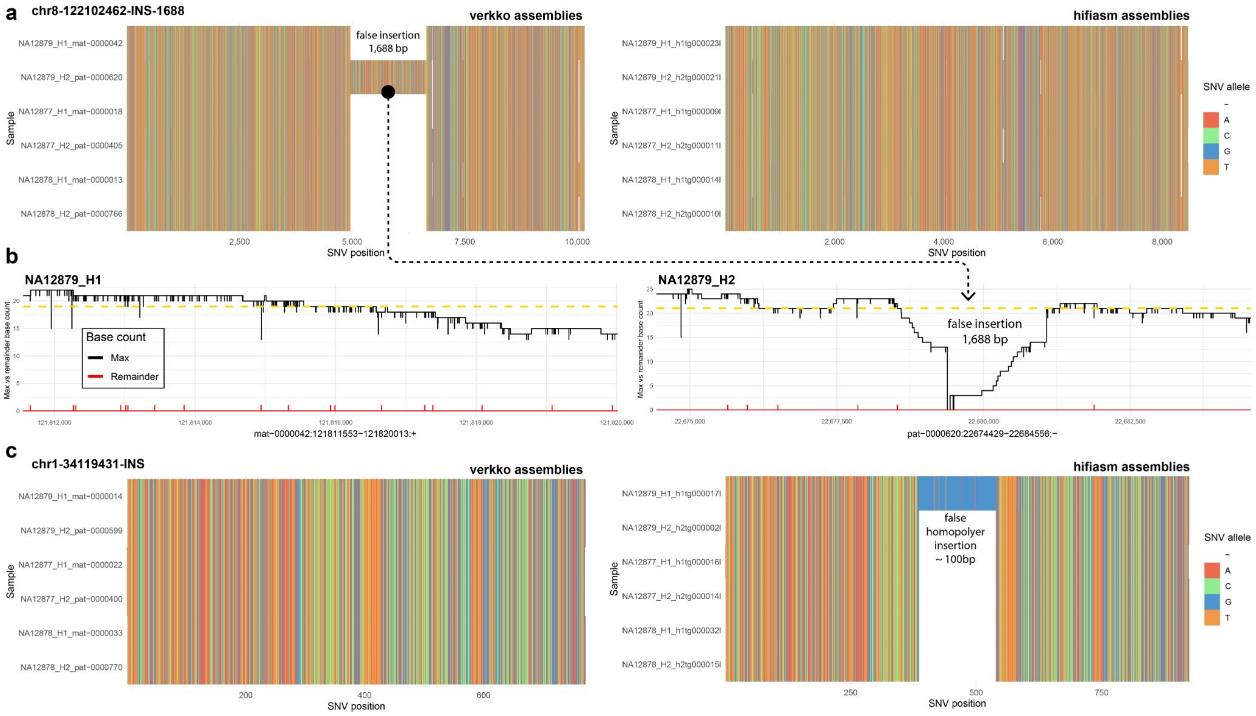

Figure S52: Examples of false *de novo* insertions in phased genome assemblies.
a) Multiple sequence alignments (MSAs) for Verkko (left) and hifiasm (right) assemblies. The top two rows represent both haplotypes for the child (G3-NA12879) while the other four rows represent G2 parental haplotypes (NA12877-father, NA12878-mother). False insertion is visible as a piece of DNA present only in the child. **b**) Long-read (PacBio HiFi) alignments to the child's (G3-NA12879) assemblies. Coverage of the most frequent base at each position is shown as a black line. Coverage of the second most frequent base is shown as the red line. False insertion in haplotype 2 of G3-NA12879 is visible as low read support visible as clear dip in the coverage of HiFi reads of the false insertion. **c**) MSAs for Verkko (left) and hifiasm (right) assemblies. The top two rows represent the child's (G3-NA12879) haplotypes while the other four rows represent G2 parental haplotypes. False insertion is visible as a piece of DNA present only in haplotype 1 of the child and being composed of only G's.

REFERENCES

Audano, Peter A., Arvis Sulovari, Tina A. Graves-Lindsay, Stuart Cantsilieris, Melanie Sorensen, Annemarie E. Welch, Max L. Dougherty, et al. 2019. “Characterizing the Major Structural Variant Alleles of the Human Genome.” *Cell* 176 (3): 663–75.e19.

Chu, Chong, Rebeca Borges-Monroy, Vinayak V. Viswanadham, Soohyun Lee, Heng Li, Eunjung Alice Lee, and Peter J. Park. 2021. “Comprehensive Identification of Transposable Element Insertions Using Multiple Sequencing Technologies.” *Nature Communications* 12 (1): 3836.

Conrad, Donald F., Jonathan E. M. Keebler, Mark A. DePristo, Sarah J. Lindsay, Yujun Zhang, Ferran Casals, Youssef Idaghdour, et al. 2011. “Variation in Genome-Wide Mutation Rates within and between Human Families.” *Nature Genetics* 43 (7): 712–14.

Cooper, Gregory M., Bradley P. Coe, Santhosh Girirajan, Jill A. Rosenfeld, Tiffany H. Vu, Carl Baker, Charles Williams, et al. 2011. “A Copy Number Variation Morbidity Map of Developmental Delay.” *Nature Genetics* 43 (9): 838–46.

Eberle, Michael A., Epameinondas Fritzilas, Peter Krusche, Morten Källberg, Benjamin L. Moore, Mitchell A. Bekritsky, Zamin Iqbal, et al. 2017. “A Reference Data Set of 5.4 Million Phased Human Variants Validated by Genetic Inheritance from Sequencing a Three-Generation 17-Member Pedigree.” *Genome Research* 27 (1): 157–64.

Edgar, Robert C. 2004. “MUSCLE: Multiple Sequence Alignment with High Accuracy and High Throughput.” *Nucleic Acids Research* 32 (5): 1792–97.

Garrison, Erik, Andrea Guarracino, Simon Heumos, Flavia Villani, Zhigui Bao, Lorenzo Tattini, Jörg Hagmann, et al. 2024. “Building Pangenome Graphs.” *Nature Methods*, October. https://doi.org/10.1038/s41592-024-02430-3.

Gros, Christina, Ashley D. Sanders, Jan O. Korbel, Tobias Marschall, and Peter Ebert. 2021. “ASHLEYS: Automated Quality Control for Single-Cell Strand-Seq Data.” *Bioinformatics* 37 (19): 3356–57.

Hallast, Pille, Peter Ebert, Mark Loftus, Feyza Yilmaz, Peter A. Audano, Glennis A. Logsdon, Marc Jan Bonder, et al. 2023. “Assembly of 43 Human Y Chromosomes Reveals Extensive Complexity and Variation.” *Nature* 621 (7978): 355–64.

Hao, Fengjie, Nan Wang, Honglian Gui, Yifan Zhang, Zhiyuan Wu, and Junqing Wang. 2022. “Pseudogene UBE2MP1 Derived Transcript Enhances in Vitro Cell Proliferation and Apoptosis Resistance of Hepatocellular Carcinoma Cells through miR-145-5p/RGS3 Axis.” *Aging* 14 (19): 7906–25.

Helgason, Agnar, Axel W. Einarsson, Valdís B. Guðmundsdóttir, Ásgeir Sigurðsson, Ellen D. Gunnarsdóttir, Anuradha Jagadeesan, S. Sunna Ebenesersdóttir, Augustine Kong, and Kári Stefánsson. 2015. “The Y-Chromosome Point Mutation Rate in Humans.” *Nature Genetics* 47 (5): 453–57.

Huang, Neng, and Heng Li. 2023. “Compleasm: A Faster and More Accurate Reimplementation of BUSCO.” *Bioinformatics* 39 (10). https://doi.org/10.1093/bioinformatics/btad595.

James Kent, W. 2002. “BLAT—The BLAST-Like Alignment Tool.” *Genome Research* 12 (4): 656–64.

Jiang, Zhaoshi, Robert Hubley, Arian Smit, and Evan E. Eichler. 2008. “DupMasker: A Tool for Annotating Primate Segmental Duplications.” *Genome Research* 18 (8): 1362–68.

Jobling, Mark A., and Chris Tyler-Smith. 2003. “The Human Y Chromosome: An Evolutionary Marker Comes of Age.” *Nature Reviews. Genetics* 4 (8): 598–612.

Katoh, Kazutaka, and Daron M. Standley. 2013. “MAFFT Multiple Sequence Alignment

Software Version 7: Improvements in Performance and Usability." *Molecular Biology and Evolution* 30 (4): 772–80.
Kent, W. James, Charles W. Sugnet, Terrence S. Furey, Krishna M. Roskin, Tom H. Pringle, Alan M. Zahler, and David Haussler. 2002. "The Human Genome Browser at UCSC." *Genome Research* 12 (6): 996–1006.
Kong, Augustine, Gudmar Thorleifsson, Daniel F. Gudbjartsson, Gisli Masson, Asgeir Sigurdsson, Aslaug Jonasdottir, G. Bragi Walters, et al. 2010. "Fine-Scale Recombination Rate Differences between Sexes, Populations and Individuals." *Nature* 467 (7319): 1099–1103.
Liao, Wen-Wei, Mobin Asri, Jana Ebler, Daniel Doerr, Marina Haukness, Glenn Hickey, Shuangjia Lu, et al. 2023. "A Draft Human Pangenome Reference." *Nature* 617 (7960): 312–24.
Li, Heng, Jonathan M. Bloom, Yossi Farjoun, Mark Fleharty, Laura Gauthier, Benjamin Neale, and Daniel MacArthur. 2018. "A Synthetic-Diploid Benchmark for Accurate Variant-Calling Evaluation." *Nature Methods* 15 (8): 595–97.
Porubsky, David, Xavi Guitart, Dongahn Yoo, Philip C. Dishuck, William T. Harvey, and Evan E. Eichler. 2024. "SVbyEye: A Visual Tool to Characterize Structural Variation among Whole-Genome Assemblies." *bioRxiv*. https://doi.org/10.1101/2024.09.11.612418.
Porubsky, David, Ashley D. Sanders, Aaron Taudt, Maria Colomé-Tatché, Peter M. Lansdorp, and Victor Guryev. 2020. "breakpointR: An R/Bioconductor Package to Localize Strand State Changes in Strand-Seq Data." *Bioinformatics* 36 (4): 1260–61.
Quinlan, Aaron R., and Ira M. Hall. 2010. "BEDTools: A Flexible Suite of Utilities for Comparing Genomic Features." *Bioinformatics* 26 (6): 841–42.
Rhie, Arang, Sergey Nurk, Monika Cechova, Savannah J. Hoyt, Dylan J. Taylor, Nicolas Altemose, Paul W. Hook, et al. 2023. "The Complete Sequence of a Human Y Chromosome." *Nature* 621 (7978): 344–54.
Sanders, Ashley D., Mark Hills, David Porubský, Victor Guryev, Ester Falconer, and Peter M. Lansdorp. 2016. "Characterizing Polymorphic Inversions in Human Genomes by Single-Cell Sequencing." *Genome Research* 26 (11): 1575–87.
Sasani, Thomas A., Brent S. Pedersen, Ziyue Gao, Lisa Baird, Molly Przeworski, Lynn B. Jorde, and Aaron R. Quinlan. 2019. "Large, Three-Generation Human Families Reveal Post-Zygotic Mosaicism and Variability in Germline Mutation Accumulation." *eLife* 8 (September). https://doi.org/10.7554/eLife.46922.
Smolka, Moritz, Luis F. Paulin, Christopher M. Grochowski, Dominic W. Horner, Medhat Mahmoud, Sairam Behera, Ester Kalef-Ezra, et al. 2024. "Detection of Mosaic and Population-Level Structural Variants with Sniffles2." *Nature Biotechnology* 42 (10): 1571–80.
Sudmant, Peter H., Tobias Rausch, Eugene J. Gardner, Robert E. Handsaker, Alexej Abyzov, John Huddleston, Yan Zhang, et al. 2015. "An Integrated Map of Structural Variation in 2,504 Human Genomes." *Nature* 526 (7571): 75–81.
Vollger, Mitchell R., Philip C. Dishuck, William T. Harvey, William S. DeWitt, Xavi Guitart, Michael E. Goldberg, Allison N. Rozanski, et al. 2023. "Increased Mutation and Gene Conversion within Human Segmental Duplications." *Nature* 617 (7960): 325–34.
Vollger, Mitchell R., Philip C. Dishuck, Melanie Sorensen, Annemarie E. Welch, Vy Dang, Max L. Dougherty, Tina A. Graves-Lindsay, Richard K. Wilson, Mark J. P. Chaisson, and Evan E. Eichler. 2019. "Long-Read Sequence and Assembly of Segmental Duplications." *Nature Methods* 16 (1): 88–94.
