# Supplementary material for: Human de novo mutation rates from a four-generation pedigree reference
Source: Nature. 2025 Apr 23;643(8071):427–36. doi: 10.1038/s41586-025-08922-2 (PMC12240836; doi:10.1038/s41586-025-08922-2)
Supplement: Supplementary file 2 — Reporting Summary [file 41586_2025_8922_MOESM2_ESM.pdf]

Corresponding author(s): Evan E Eichler
Last updated by author(s): Feb 13, 2025

Reporting Summary

Nature Portfolio wishes to improve the reproducibility of the work that we publish. This form provides structure for consistency and transparency in reporting. For further information on Nature Portfolio policies, see our Editorial Policies and the Editorial Policy Checklist.

Statistics

For all statistical analyses, confirm that the following items are present in the figure legend, table legend, main text, or Methods section.

n/a	Confirmed	
☐	☒	The exact sample size (*n*) for each experimental group/condition, given as a discrete number and unit of measurement
☐	☒	A statement on whether measurements were taken from distinct samples or whether the same sample was measured repeatedly
☐	☒	The statistical test(s) used AND whether they are one- or two-sided<br>*Only common tests should be described solely by name; describe more complex techniques in the Methods section.*
☒	☐	A description of all covariates tested
☒	☐	A description of any assumptions or corrections, such as tests of normality and adjustment for multiple comparisons
☐	☒	A full description of the statistical parameters including central tendency (e.g. means) or other basic estimates (e.g. regression coefficient) AND variation (e.g. standard deviation) or associated estimates of uncertainty (e.g. confidence intervals)
☐	☒	For null hypothesis testing, the test statistic (e.g. *F*, *t*, *r*) with confidence intervals, effect sizes, degrees of freedom and *P* value noted<br>*Give P values as exact values whenever suitable.*
☒	☐	For Bayesian analysis, information on the choice of priors and Markov chain Monte Carlo settings
☒	☐	For hierarchical and complex designs, identification of the appropriate level for tests and full reporting of outcomes
☒	☐	Estimates of effect sizes (e.g. Cohen's *d*, Pearson's *r*), indicating how they were calculated

Our web collection on statistics for biologists contains articles on many of the points above.

Software and code

Policy information about availability of computer code

Data collection	The software used to collect sequencing data are Pacific Biosciences SMRT Link (v11.0.0, 11.0.1, and 12.0) and Oxford Nanopore Technologies MinKNOW software (v21.02.17 - 23.04.5).
Data analysis	Custom code and pipelines used in this study are publicly available via the following GitHub repositories:<br>https://github.com/orgs/Platinum-Pedigree-Consortium/repositories<br><br>Publicly available software used in this study include:<br>BWA-MEM (v0.7.17-r1188), SAMtools (v1.10), sambamba (v1.0), Verkko (v1.3.1 and v1.4.1), hifiasm (v0.19.5), minimap2 (v2.21, >=v2.24) winnowmap (v2.03), Meryl (v1.0), Merqury (v1.1), TRGT (v0.7.0-493ef25), rustybam (v0.1.33), HiPhase (v1.0.0-f1bc7a8), Clair3 (v1.0.7), GATK (v4.3.0.0), DeepVariant (v1.4.0 and v1.6.0), WFMASH (v0.13.1), PGGB (v0.6.0), VCFBUB (v0.1.0), VCFWAVE (v1.0.3), pbmm2 (v1.1.0), TRF (v4.09.1), RepeatMasker (v4.1.0, v4.1.2-p1, and v4.1.6), BCFtools (v1.16 and v1.17), VCFtools (v0.1.16), ISOGG (v15.73), BEAST (v1.10.4), RAxML (v8.2.10), Tree-Annotator (v.1.10.4), FigTree (v.1.4.4), HMMER (v.3.3.2dev), Gepard (v2.0), PAV (v2.3.4), DipCall (v0.3), MAFFT (v7.508), compleasm (v0.2.4), TRGT-denovo (v0.1.3), Variation graph toolkit (vg, v.1.40.0), muscle (v3.8.31), ASHLEYS (v0.2.0), Flagger (v0.3.3), NucFreq (v0.1), compleasm (v0.2.4), SVbyEye (v0.99.0), SVPOP (v3.4.0), PBSV (v2.9.0), Sniffles (v0.12.0), Sawfish (v2.2), Integrative Genomics Viewer (IGV, v2.16.0), Guppy (v6.3.7 and v6.5.7)<br>We also used following R packages: fastseg (v1.46.0), breakpointR (v1.15.1), regioneR (v1.32.0), DECIPHER (v2.28.0), Biostrings (v2.70.2), StrandPhaseR (v0.99)

For manuscripts utilizing custom algorithms or software that are central to the research but not yet described in published literature, software must be made available to editors and reviewers. We strongly encourage code deposition in a community repository (e.g. GitHub). See the Nature Portfolio guidelines for submitting code & software for further information.

Data

Policy information about availability of data

All manuscripts must include a data availability statement. This statement should provide the following information, where applicable:

- Accession codes, unique identifiers, or web links for publicly available datasets
- A description of any restrictions on data availability
- For clinical datasets or third party data, please ensure that the statement adheres to our policy

All underlying data from 28 members of the family are available as part of AWS Open Data program or dbGaP.
Variant calls, mapped sequencing data, and assemblies for 23 family members (G1-GM12889, G1-GM12890, G1-GM12891, G1-GM12892, G2-GM12877, G2-GM12878, G3-GM12879, G3-GM12881, G3-GM12882, G3-GM12885, G3-GM12886, G3-200080-spouse, G4-200081, G4-200082, G4-200084, G4-200085, G4-200086, G4-200087, G3-200100-spouse, G4-200101, G4-200102, G4-200104, G4-200106) consented for their data to be publicly accessible similar to the 1000 Genomes Project samples to allow for development of new technologies, study of human variation, research on the biology of DNA, and study of health and disease are available via the AWS Open Data program: s3://platinum-pedigree-data/.

See https://github.com/Platinum-Pedigree-Consortium/Platinum-Pedigree-Datasets for specific details on how to access.
In addition, mapped sequencing data and assemblies for five family members (G3-NA12883, G3-NA12884, G3-NA12887, G4-200103, G4-200105) that are not consented for open access are available via dbGaP under Accession ID: phs003793.v1.p1 (Platinum Pedigree Consortium long-read sequencing). This includes also variant calls for the whole family (28 members).

The tandem repeat catalogs are available on Zenodo DOI: 10.5281/zenodo.13178746.

The Y-chromosomal assembly for a closely related R1b haplogroup sample HG00731 was downloaded from the Human Genome Structural Variation Consortium IGSR site (https://ftp.1000genomes.ebi.ac.uk/vol1/ftp/data_collections/HGSVC3/working/20230927_verkko_batch2/assemblies/HG00731/).

Research involving human participants, their data, or biological material

Policy information about studies with human participants or human data. See also policy information about sex, gender (identity/presentation), and sexual orientation and race, ethnicity and racism.

Reporting on sex and gender	Research participants self-report gender as male, female or other. In the manuscript, sex is reported based on genetic analysis.
Reporting on race, ethnicity, or other socially relevant groupings	Research participants self-report race as American Indian/Alaska Native, Asian, Black or African American, Native Hawaiian or other Pacific Islander, or White. Research participants self-report ethnicity as Hispanic/Latino or Not Hispanic/Latino. They also can select that they do not wish to provide some or all of the information.
Population characteristics	The contributing study population was selected not for disease but for families of large sibship size, living parents, and living grandparents as described by Dausset et al., 1990, Genomics. Four individuals from the first generation were enrolled at ages 75 to 83 years; two individuals from the second generation were enrolled at ages 57 and 58; seven individuals in the third generation were enrolled at ages 23 to 36, and most recently, the spouses of the third generation were enrolled at ages 58 and 71, and the fourth generation were enrolled at ages 24 to 49. Fourteen individuals are male. Fourteen individuals are female. All family members are White and Non-Hispanic/Latino.
Recruitment	Identification and recruitment of large families was through community engagement and word-of-mouth. When eligible families (4 grandparents, 2 parents, 6 or more children) were identified, family members advocated and recruited their immediate family members into the study. There was no selection based on sex or gender. Although there was no selection based on race or ethnicity, all families were ultimately Caucasian/White due to the demographics of the communities involved.
Ethics oversight	The study is approved and overseen by the Institutional Review Board of the University of Utah under IRB_00065564.

Note that full information on the approval of the study protocol must also be provided in the manuscript.

Field-specific reporting

Please select the one below that is the best fit for your research. If you are not sure, read the appropriate sections before making your selection.

☒ Life sciences ☐ Behavioural & social sciences ☐ Ecological, evolutionary & environmental sciences

For a reference copy of the document with all sections, see nature.com/documents/nr-reporting-summary-flat.pdf

Life sciences study design

All studies must disclose on these points even when the disclosure is negative.

Sample size	Sample size was defined based on the availability of consented family members of the CEPH (1463) family.

Data exclusions	We excluded from the analysis three individuals (NA12880, NA12888, and NA12893) who did not choose consent for biobanking and broad data access.
Replication	Whole-genome sequencing was conducted using five complimentary short- and long-read sequencing platforms on the same DNA samples to create a high level of confidence in the genomic data. Two hybrid genome assembly pipelines, hifiasm and Verkko, were applied to reinforce the confidence in the highly contiguous phased genome assemblies. With this rigor, further attempts at replication were not done.
Randomization	N/A: this was not an interventional trial.
Blinding	N/A: this was not an interventional trial.

Reporting for specific materials, systems and methods

We require information from authors about some types of materials, experimental systems and methods used in many studies. Here, indicate whether each material, system or method listed is relevant to your study. If you are not sure if a list item applies to your research, read the appropriate section before selecting a response.

Materials & experimental systems

n/a	Involved in the study	
☒	☐	Antibodies
☐	☒	Eukaryotic cell lines
☒	☐	Palaeontology and archaeology
☒	☐	Animals and other organisms
☒	☐	Clinical data
☒	☐	Dual use research of concern
☒	☐	Plants

Methods

n/a	Involved in the study	
☒	☐	ChIP-seq
☒	☐	Flow cytometry
☒	☐	MRI-based neuroimaging

Eukaryotic cell lines

Policy information about cell lines and Sex and Gender in Research

Cell line source(s)	Coriell Institute for Medical Research, NIGMS Human Genetic Cell Repository, CEPH collection. Cell line IDs for 14 members of the CEPH 1463 family: G1-GM12889, G1-GM12890, G1-GM12891, G1-GM12892, G2-GM12877, G2-GM12878, G3-GM12879, G3-GM12881, G3-GM12882, G3-GM12883, G3-GM12884, G3-GM12885, G3-GM12886, G3-GM12887.<br>EBV transformed lymphoblastoid cell lines were generated for G3 spouses and G4 family members (n=13): G3-200080-spouse, G4-200081, G4-200082, G4-200084, G4-200085, G4-200086, G4-200087, G3-200100-spouse, G4-200101, G4-200102, G4-200103, G4-200104, G4-200106.
Authentication	Cell lines were authenticated by whole-genome sequencing of the DNA and subsequent variant calling. Sequence results must match a) the sex of the individual, b) sequencing results from blood-derived DNA from the same individual, and c) inheritance pattern of parents and offspring.
Mycoplasma contamination	Cell lines were not tested for mycoplasma contamination.
Commonly misidentified lines (See ICLAC register)	No commonly misidentified cell lines were used in the study.

Plants

Seed stocks	N/A
Novel plant genotypes	N/A
Authentication	N/A
